# YBX1 Confers immunosuppressive bone metastatic traits in non-small cell lung cancer

**DOI:** 10.1038/s41467-026-73931-2

**Published:** 2026-06-13

**Authors:** Kai Zhang, Bin Li, Qingshui Wang, Xiuli Zhang, Cheng Cheng, Mengyao Lv, Mengyang Huang, Zihui Liang, Zijie Xie, Yao Lin, Yang Zhao, Lilin Ge, Jing Chen

**Affiliations:** 1 Nanjing University of Chinese Medicine, Nanjing, China; 2 Department of Respiratory Medicine, Nanjing First Hospital, Nanjing University of Chinese Medicine, Nanjing, China; 3 Department of Respiratory Medicine, Nanjing First Hospital, Nanjing Medical University, Nanjing, China; 4 School of Medicine, Nanjing University of Chinese Medicine, Nanjing, China; 5 Jiangsu Province Engineering Research Center of TCM Health Preservation, Nanjing University of Chinese Medicine, Nanjing, China; 6 Affiliated People’s Hospital, Fujian-Macao Science and Technology Cooperation Base of Traditional Chinese Medicine-Oriented Chronic Disease Prevention and Treatment, Fujian-Hong Kong-Macau-Taiwan Collaborative Laboratory for the Inheritance and Innovation of Traditional Chinese Medicine, Fujian University of Traditional Chinese Medicine, Fuzhou, Fujian, China; 7 College of Integrative Medicine, Academy of Integrative Medicine, Fujian University of Traditional Chinese Medicine, Fuzhou, Fujian, China; 8 Department of Oncology, the Second Hospital of Nanjing, Affiliated to Nanjing University of Chinese Medicine, Nanjing, China

**Keywords:** Non-small-cell lung cancer, Non-small-cell lung cancer

## Abstract

The spread of lung cancer to bone is a devastating complication often linked to resistance against immunotherapy, but the reasons for this connection are poorly understood. Here we show that the transcription factor YBX1 acts as a central regulator driving both bone metastasis and the formation of an immunosuppressive environment in non-small cell lung cancer (NSCLC). YBX1 achieves this by activating distinct signaling pathways (IL6 and CCL5, respectively). Mechanistically, YBX1 protein levels are controlled by glycosylation that marks it for autophagic degradation inside cells. Notably, reduced YBX1 glycosylation was observed in highly bone-metastatic NSCLC cells. Importantly, we identified a drug candidate, Icaritin, which boosts this sugar-modification, leading to YBX1 degradation. This dual action inhibits bone metastasis and re-sensitizes tumors to immune attack. Our work reveals YBX1 as a promising single target for combating bone spread and overcoming immunotherapy resistance.

## Abstract (web summary)

Bone metastases derived from lung cancer typically exhibit an immunosuppressive microenvironment. This study identifies a YBX1 glycosylation–dependent mechanism that drives both metastasis and immunosuppression, and proposes the small molecule Icaritin as a potential therapeutic strategy through YBX1 degradation.

## Introduction

Lung cancer is the leading cause of cancer-related deaths, particularly in its advanced stages, where the incidence of bone metastasis significantly increases, severely impacting patients’ quality of life and prognosis^1,2^. Bone metastasis leads to severe complications such as intense pain, pathological fractures, and spinal cord compression, marking the progression into a stage that is notoriously difficult to treat^3,4^. Although studies have shown that cancer cells migrate to and colonize bone through various pathways, the specific molecular mechanisms regulating this process, especially in lung cancer, remain largely unelucidated^5^.

The process of bone metastasis in lung cancer involves complex biological processes, including cancer cell migration, invasion, colonization, and interactions with the bone microenvironment^6,7^. The occurrence of bone metastasis in lung cancer not only depends on the intrinsic characteristics of tumor cells but is also influenced by the host immune microenvironment. Particularly, the immunosuppressive microenvironment observed during lung cancer bone metastasis has become a critical factor affecting treatment efficacy^8^. Studies indicate that the formation of an immunosuppressive microenvironment during lung cancer bone metastasis weakens the body’s antitumor immune response and diminishes the effectiveness of existing immunotherapies^9^. Therefore, exploring strategies to improve this immunosuppressive microenvironment to enhance the efficacy of immunotherapy remains a significant scientific question in current research.

Understanding the molecular mechanisms underlying the complexity and multifactorial nature of lung cancer bone metastasis is crucial for developing effective therapeutic approaches. The role of transcription factors in the progression of lung cancer has garnered considerable attention due to their key functions in regulating gene expression, controlling cell proliferation, differentiation, and displaying abnormal activity in various cancers^10^.

In this context, we identified transcription factor YBX1 is the key regulator that drives lung cancer bone metastasis and its immunosuppressive microenvironment, particularly through IL6- and CCL5-mediated pathway respectively, revealing a complex network of actions. In addition, we found the YBX1 protein stability is governed by O-GlcNAc transferase (OGT)-mediated glycosylation at T271, which promotes its degradation by mitophagy. These findings suggest that YBX1 may serve as a promising therapeutic target for inhibiting NSCLC bone metastasis and reversing immune resistance. Based on these observations, we identified a potentially therapeutic small molecule drug, ICA, which exhibits potent inhibition against YBX1. This present study provides a theoretical foundation and experimental basis for developing therapeutic strategies targeting NSCLC bone metastasis.

## Results

### Identification of YBX1 as a driver for NSCLC bone metastasis via osteolytic niche remodeling

**Fig. 1:**
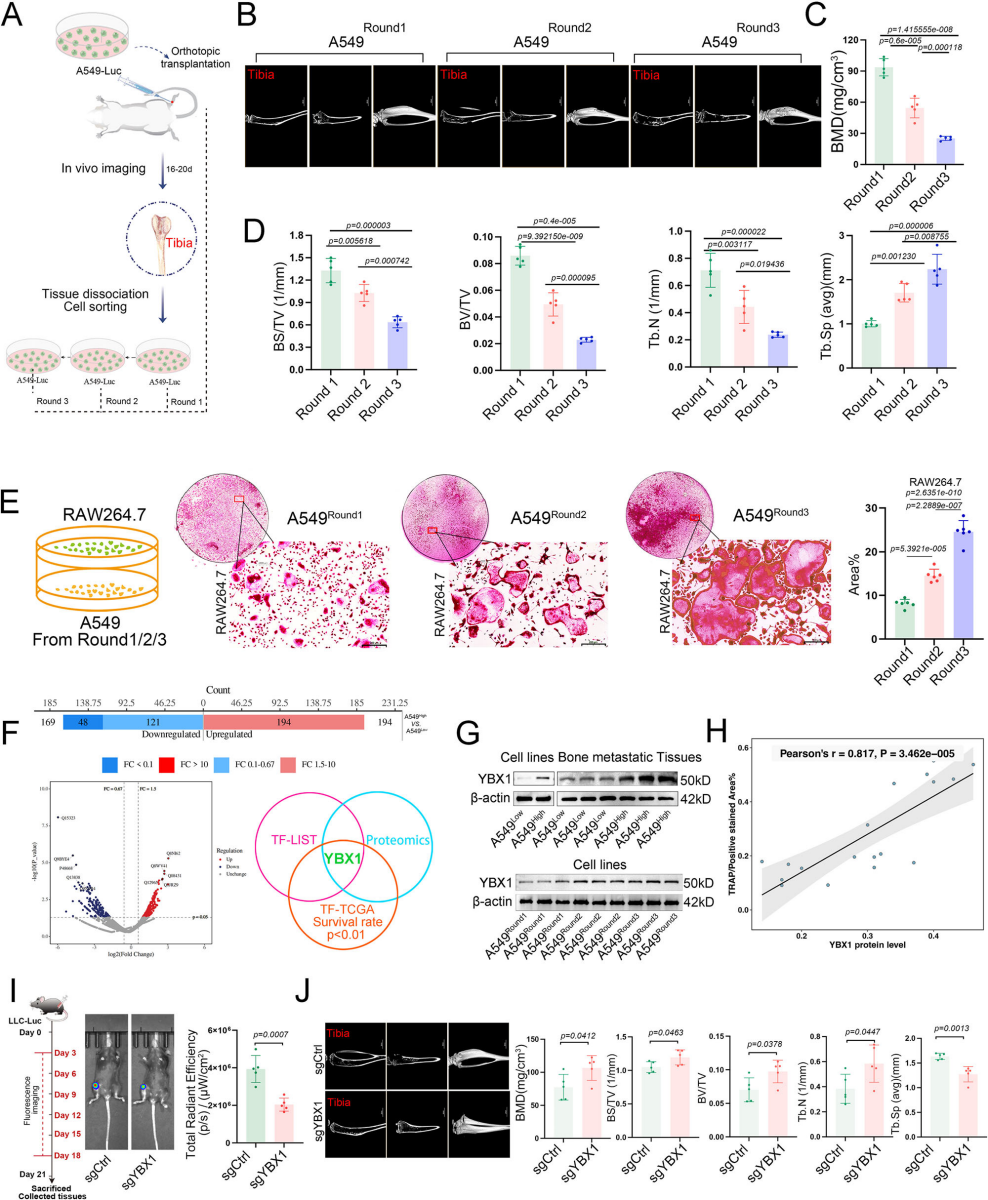
Investigation of the key role and mechanism of YBX1 in bone metastasis of non-small cell lung cancer. **A** Flowchart illustrating the establishment of an orthotopic bone metastasis animal model and a highly bone-metastatic cell line through an iterative cycle of “intra-tibial injection → isolation of bone metastatic tumors → primary culture of bone metastatic cells → re-injection”. The schematic diagram was created by the author using Adobe Illustrator. **B** Representative micro-CT images of tibiae after intra-tibial injection of different passages of A549 cells (Round1, Round2, Round3). **C** Quantitative analysis of Bone Mineral Density (BMD) corresponding to panel (**B**). *Biological replicates n* = *5, mice; One-way ANOVA (multivariate comparison: Tukey); Mean *± SD. **D** Quantitative analysis of bone histomorphometric parameters, including Bone Surface/Tissue Volume (BS/TV), Bone Volume/Tissue Volume (BV/TV), Trabecular Number (Tb.N), and Trabecular Separation (Tb.Sp). *Biological replicates n* = *5, mice, One-way ANOVA (multivariate comparison: Tukey); Mean *± SD. **E** Representative Tartrate-Resistant Acid Phosphatase (TRAP) staining images and quantitative analysis of TRAP-positive osteoclasts after co-culture of different passages of A549 cells with osteoclast precursor RAW264.7 cells (*Scale bar: 200 μm*). *Biological replicates n* = *6, independent experiments, One-way ANOVA (multivariate comparison: Tukey); Mean *± SD. The schematic diagram was created by the author using Adobe Illustrator. **F** (Left) Volcano plot of proteomic differential analysis between A549 cells with high/low bone metastatic potential; (Right) Venn diagram showing the intersection between differentially expressed proteins and prognosis-associated transcription factors from the TCGA database (https://portal.gdc.cancer.gov/), suggesting YBX1 as a key factor. **G** Representative western blot images demonstrating YBX1 protein expression in different round of A549 cells and in bone metastatic tumor tissues derived from them. *Biological replicates: n* = *3, independent experiments*. **H** Scatter plot of correlation analysis between YBX1 protein expression levels and the degree of osteoclast activation (percentage of TRAP-positive area) in the co-culture system. *Biological replicates n* = *18, independent experiments, Pearson’s correlation test (Two-tailed)*. *The shaded area indicates the 95% confidence interval for the linear fit*. **I** (Left) In vivo fluorescence imaging (representative) of tibial metastasis after constructing a bone metastasis model with YBX1-knockout Lewis lung carcinoma cells; (Right) Quantitative analysis of fluorescence signal intensity. *Biological replicates n* = *5, mice, Unpaired t test (Two-tailed); Mean *± SD. The schematic diagram was created by the author using Adobe Illustrator. **J** (Right) Representative micro-CT images of tibiae corresponding to the experimental groups in (**I**); (left) Quantitative analysis of the degree of bone destruction. *Biological replicates n* = *5, mice, Unpaired t test (Two-tailed); Mean *± SD.

To explore the mechanisms underlying bone metastasis, we established an orthotopic NSCLC bone metastasis model through iterative cycles of “Intratibial injection → Bone metastatic tumor isolation → Primary culture of bone metastatic cells → Re-injection” (Fig. 1A). This approach generated stable bone-tropic NSCLC cell lines (A549^High-M^ and H1975^High-M^) with enhanced osteolytic capacity, as validated by micro-CT quantification of bone lesions (Fig. 1B, C). By analyzing the BS/TV, BV/TV, Tb.N, Tb.Sp, we confirmed the osteolytic destruction (Fig. 1D). To assess the effect on osteoclast activity, we performed a TRAP assay following co-culture of RAW264.7 cells with different rounds of cells. The results indicated that Round 3 cells promoted osteoclast viability most significantly (Fig. 1E). Based on the above findings, we designated the Round 3 cells as lung adenocarcinoma cell lines with high bone-metastatic potential (A549^High-M^, H1975 ^High-M^) and the cells maintained under standard culture conditions without in vivo selection as low bone-metastatic potential cells (A549^Low-M^, H1975 ^Low-M^).

Transcriptional reprogramming plays a critical roles in bone metastasis. Thus, we performed Proteomics on high (A549^High-M^) and low bone-metastatic potential (A549^Low-M^) cell lines to identify transcription factors potentially involved in bone metastasis. By intersecting differentially expressed genes with prognostically significant transcription factors from TCGA datasets, we identified YBX1 as a potential regulator (Fig. 1F). Subsequent validation confirmed consistent upregulation of YBX1 in different round cells and their derived bone metastatic tissues (Fig. 1G). In addition, we analyzed the expression of the YBX1 protein in successive rounds of cells and its corresponding impact on TRAP staining of RAW264.7 cells. The results revealed that the expression level of YBX1 was proportional to the TRAP positivity rate, indicating that YBX1 expression in tumor cells participates in the activation of osteoclasts (Fig. 1H, r = *0.817, p* < *0.0001*). To investigate the role of YBX1 in NSCLC bone metastasis, we established a bone metastasis model using YBX1-knockout Lewis Lung Carcinoma (LLC) cells. In vivo fluorescence imaging shows that YBX1 knockout reduces bone metastasis formation (Fig. 1I). Micro-CT analysis of bone structure revealed that YBX1 depletion alleviated bone destruction (Fig. 1J). These results demonstrated that YBX1 depletion significantly reduced metastatic tumor formation in bone.

To further confirm the pro-metastatic effects of YBX1, YBX1 was ectopically expressed in low-metastatic potential cells (A549^Low-M^ and H1975^Low-M^) and depleted in high-metastatic cells (A549^High-M^ and H1975^High-M^) (Supplementary Fig. S1A). Transwell-migration assay revealed that YBX1 forced expression enhanced cellular migration capacity, while its knockout suppressed migration (Supplementary Fig. S1B). The hallmark of bone metastasis in lung cancer is osteoclast-mediated bone destruction, pathologically characterized by predominant osteolytic lesions. Once tumor cells colonize the bone, a self-reinforcing vicious cycle emerges through interactions among tumor cells, osteoblasts, and osteoclasts. This process activates the receptor activator of nuclear factor-κB ligand (RANKL), which binds to its receptor RANK to stimulate osteoclastogenesis. Thereby, we examined whether YBX1 regulates RANKL expression. As shown in Supplementary Fig. S1C, D, ectopic expression of YBX1 upregulated RANKL levels, whereas knockdown of YBX1 downregulated RANKL in tumor cells, suggesting that YBX1 promotes bone metastasis by activating the RANKL/RANK signaling axis. To further validate YBX1’s impact on osteoclast activation, we co-cultured high-metastatic cells with RAW264.7 pre-osteoclasts. Downregulation of osteoclast markers (integrin β3, MMP9, CTSK, and DC-STAMP) were observed in osteoclasts when co-cultured with YBX1-depleted NSCLC cells, which indicates reduced osteoclast activation upon YBX1 suppression (Supplementary Fig. S1E, F). These findings collectively demonstrate that YBX1 facilitates NSCLC bone metastasis by enhancing tumor cell invasiveness and promoting RANKL-mediated osteoclast activation.

### Clinical analysis confirmed that high YBX1 expression was linked to bone metastasis and poor prognosis

**Fig. 2:**
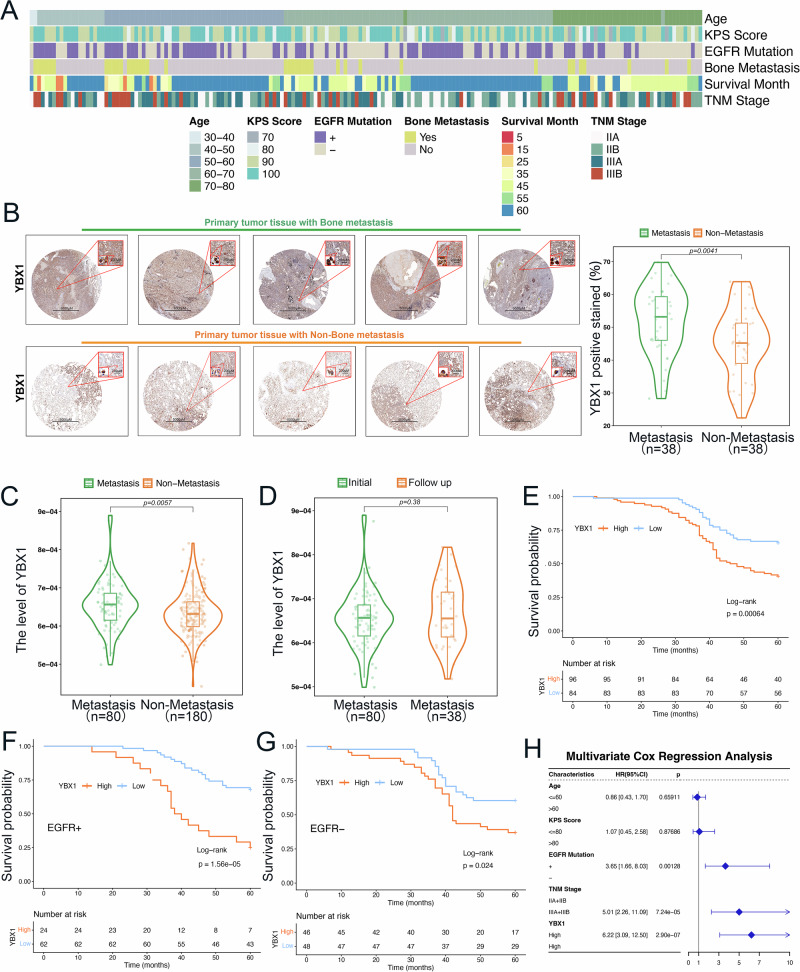
Correlation between YBX1 expression and bone metastasis, and its prognostic value in lung adenocarcinoma. **A** Heatmap of the clinical characteristics of the 180 NSCLC patient cohort included in this study, including age, KPS score, EGFR mutation status, bone metastasis status (all absent at initial diagnosis), and TNM stage. **B** (Left) Representative immunohistochemistry staining images showing YBX1 expression levels in primary tumor tissues from patients without bone metastasis (top, *n = 38, patient samples*) and with bone metastasis (bottom, *n* = *38, patient samples*); (Right) Quantitative statistical analysis of the percentage of YBX1-positive staining between the two groups. *Unpaired t test (Two-tailed). Non-metastasis: n* = *38, patient samples, minima* = *22.4, maxima* = *63.9, center* = *45.2, Q1/Lower Quartile* = *38.975, Q3/Upper Quartile* = *51.275, Lower Whisker* = *22.4, Upper Whisker* = *63.9, IQR / Interquartile Range* = *12.3; Metastasis: n* = *38, minima* = *28.3, maxima* = *69.8, center* = *53.2, Q1/Lower Quartile* = *46.05, Q3/Upper Quartile* = *59.35, Lower Whisker* = *28.3, Upper Whisker* = *69.8, IQR / Interquartile Range* = *13.3*. **C** Comparison of YBX1 expression levels in primary tumors between 80 patients with bone metastasis at initial diagnosis and 180 patients without metastasis at initial diagnosis. *Biological replicates, Unpaired t test (Two-tailed)*. *Non-metastasis: n* = *180, patient samples, minima* = *0.00044, maxima* = *0.00082, center* = *0.00063, Q1/Lower Quartile* = *6e-04, Q3/Upper Quartile* = *0.00066, Lower Whisker* = *0.00051, Upper Whisker* = *0.00075, IQR / Interquartile Range* = *6e-05; Metastasis: n* = *80, patient samples, minima* = *5e-04, maxima* = *0.00089, center* = *0.00066, Q1/Lower Quartile* = *0.0006175, Q3/Upper Quartile* = *0.00069, Lower Whisker* = *0.00050875, Upper Whisker* = *0.00079875, IQR / Interquartile Range* = *7.25e-05. The shaded area indicates the 95% confidence interval for the linear fit*. **D** Comparison of YBX1 expression levels in primary tumors between the group with bone metastasis at initial diagnosis (*n* = *80, patient samples*) and the group that developed bone metastasis during follow-up (*n* = *38, patient samples)*. *Biological replicates, Unpaired t test (Two-tailed)*. *Non-metastasis: n* = *38, patient samples, minima* = *0.00052, maxima* = *0.00082, center* = *0.000655, Q1/Lower Quartile* = *0.00061, Q3/Upper Quartile* = *0.0007175, Lower Whisker* = *0.00052, Upper Whisker* = *0.00082, IQR / Interquartile Range* = *0.0001075; Metastasis: n* = *80, patient samples, minima* = *5e-04, maxima* = *0.00089, center* = *0.00066, Q1/Lower Quartile* = *0.0006175, Q3/Upper Quartile* = *0.00069, Lower Whisker* = *0.00050875, Upper Whisker* = *0.00079875, IQR / Interquartile Range* = *7.25e-05*. **E** Kaplan Meier curves of Progression Free Survival in patients with high and low YBX1 expression for bone metastases. (*n* = *180, patient samples, p* = *0.00064*). **F** The relationship between high and low YBX1 expression and progression free survival of bone metastases in a subgroup of EGFR mutant patients. (*n* = *86, patient samples, p* = *1.56e-05*). **G** The relationship between high and low YBX1 expression and progression-free survival of bone metastases in a subgroup of EGFR wild-type patients. (*n* = *94, patient samples, p* = *0.024*). **H** Forest plot of multivariate analysis using a Cox proportional hazards regression model for bone metastasis risk, confirming that YBX1 overexpression is an independent risk factor (*Hazard Ratio HR* = *6.22, 95% CI: 3.09-12.50*). *Biological replicates n* = *180, patient samples, Multivariate Cox proportional hazards regression analysis*.

To evaluate the relevance between YBX1 expression and bone metastasis in NSCLC, we collected primary tumor tissues and matched serum samples from 180 NSCLC patients with complete follow-up records (Fig. 2A). All patients were bone metastasis-free at initial diagnosis. During the 5-year follow-up, 38 cases developed bone metastasis. From the remaining 142 metastasis-free patients, 38 controls were randomly selected. Immunohistochemistry (IHC) analysis revealed that YBX1 expression in primary tumors was significantly higher in bone metastasis-positive cases compared to metastasis-free controls (Fig. 2B, *p* = *0.0041*). In addition, we analyzed YBX1 levels in 80 primary tumors from patients with bone metastasis at initial diagnosis. These cases exhibited significantly elevated YBX1 expression relative to the 180 initially metastasis-free cases (Fig. 2C, *p* = *0.0057*). However, no significant difference in YBX1 expression was observed between the 80 initial metastasis-positive cases and the 38 follow-up metastasis cases (Fig. 2D, *p* = *0.38*). As we known, EGFR not only drives the proliferation and survival of tumor cells but also serves as a key driver of tumor invasion and metastasis. Tumor metastasis is a multi-step “invasion-metastasis cascade” process, and EGFR plays a critical role in numerous stages, including inducing epithelial-mesenchymal transition (EMT), promoting angiogenesis, resisting anoikis, facilitating extravasation, and establishing metastatic niches^11,12^. Our survival analysis revealed that high YBX1 expression correlated with poorer prognosis, independent of EGFR mutation status (Fig. 2E-G), indicating that the bone metastatic pathway driven by YBX1 may operate independently of the canonical EGFR signaling axis. Cox proportional hazards regression confirmed YBX1 overexpression as an independent risk factor for bone metastasis (hazard ratio [HR] = 6.22, 95% CI:3.09, 12.50; Fig. 2H). Therefore, targeting YBX1 could represent a potential therapeutic strategy for patients with bone metastasis who have developed resistance to EGFR-TKIs or those with wild-type EGFR.

### YBX1 transcriptionally activates IL6 and CCL5 via liquid-liquid phase separation (LLPS)

**Fig. 3:**
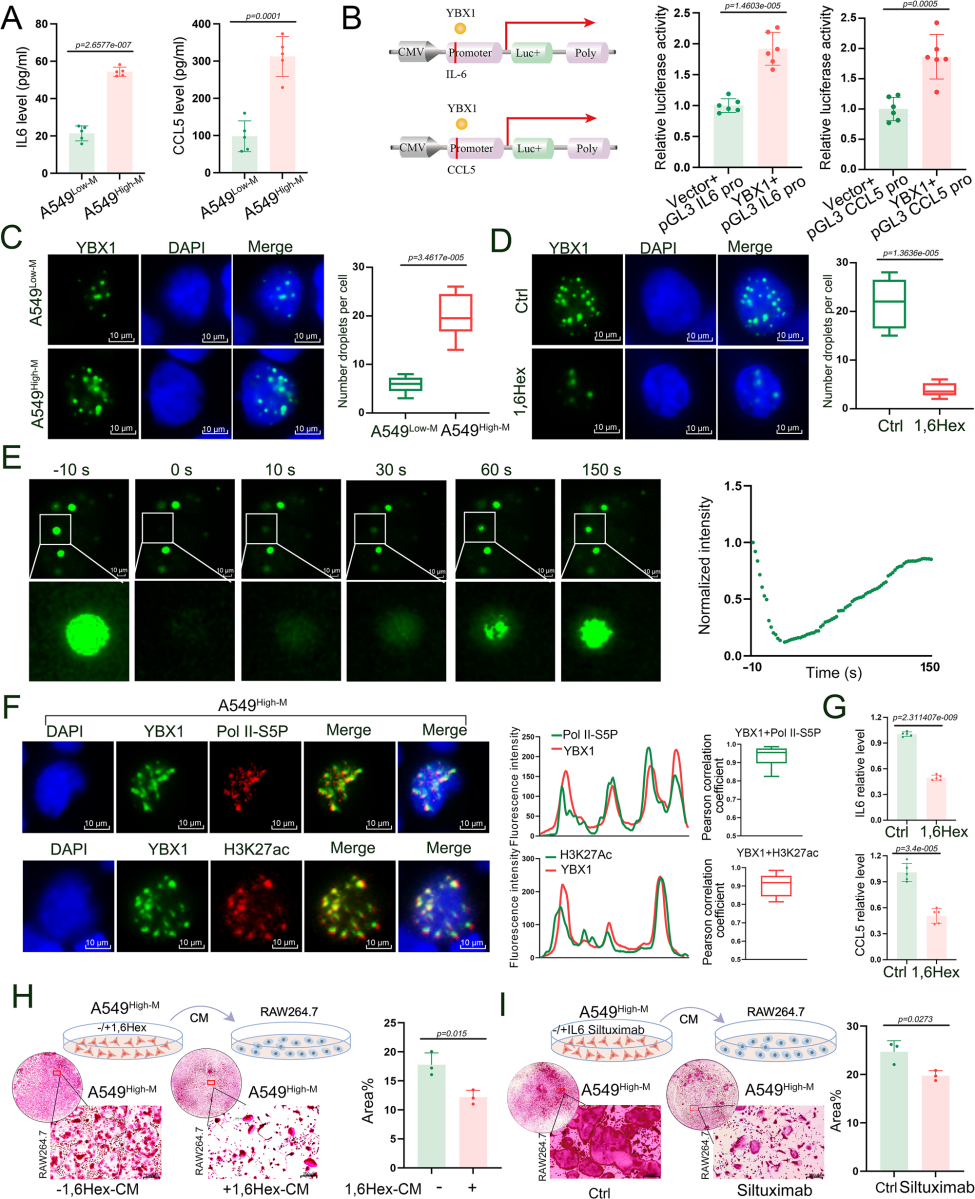
YBX1 Activates IL6/CCL5 expression and promotes osteoclast activation via liquid-liquid phase separation to form transcriptional condensates. **A** ELISA detection of IL6 and CCL5 concentrations in the serum of mice bearing A549^High-M^ tumors. *Biological replicates n* = *5, independent experiments, Unpaired t test (Two-tailed); Mean *± SD. **B** Dual-luciferase reporter assay analyzing the effect of YBX1 overexpression on the activity of the IL6 and CCL5 promoters. *Biological replicates n* = *6, independent experiments, Unpaired t test (Two-tailed); Mean *± SD. The schematic diagram was created by the author using Adobe Illustrator. **C** Representative immunofluorescence images of the number of YBX1 condensates in the nuclei of A549^High-M^ and A549^Low-M^ cells (*Scale bar: 10 μm*). *Biological replicates n* = *6, independent experiments, Unpaired t test (Two-tailed)*. *A549*^*Low-M*^*: n* = *6, minima* = *3, maxima* = *8, center* = *6, Q1/Lower Quartile* = *5.25, Q3/Upper Quartile* = *6.75, Lower Whisker* = *3, Upper Whisker* = *8, IQR / Interquartile Range* = *1.5; A549*^*High-M*^*: n* = *6, minima* = *13, maxima* = *26, center* = *19.5, Q1/Lower Quartile* = *18.25, Q3/Upper Quartile* = *23, Lower Whisker* = *13, Upper Whisker* = *26, IQR / Interquartile Range* = *4.75*. **D** Representative immunofluorescence images of the changes after treatment with 5% 1,6-Hex (*Scale bar: 10 μm*). *Biological replicates n* = *6, independent experiments, Unpaired t test (Two-tailed)*. *Ctrl: n* = *6, minima* = *15, maxima* = *28, center* = *22, Q1/Lower Quartile* = *17.5, Q3/Upper Quartile* = *25.75, Lower Whisker* = *15, Upper Whisker* = *28, IQR / Interquartile Range* = *28.25; 1,6Hex: n* = *6, minima* = *2, maxima* = *6, center* = *3.5, Q1/Lower Quartile* = *3, Q3/Upper Quartile* = *4.75, Lower Whisker* = *2, Upper Whisker* = *6, IQR / Interquartile Range* = *1.75*. **E** Representative images of the dynamics of YBX1 condensates in fluorescence bleaching recovery experiment (*Scale bar: 10 μm*). *Biological replicates n* = *6, independent experiments*. **F** Representative images of nuclear co-localization of YBX1 condensates with transcriptional activity markers p-RNA Pol II (Ser5) and H3K27ac (*Scale bar: 10 μm*). *YBX1+Pol II-S5P: n* = *20, perspective, minima* = *0.825, maxima* = *0.987, center* = *0.955, Q1/Lower Quartile* = *0.90275, Q3/Upper Quartile* = *0.97625, Lower Whisker* = *0.825, Upper Whisker* = *0.987, IQR/Interquartile Range* = *0.0735; YBX1 + H3K27ac: n* = *20, perspective, minima* = *0.814, maxima* = *0.985, center* = *0.9185, Q1/Lower Quartile* = *0.846, Q3/Upper Quartile* = *0.95175, Lower Whisker* = *0.814, Upper Whisker* = *0.985, IQR / Interquartile Range* = *0.10575*. **G** RT-qPCR analysis of IL6 and CCL5 mRNA expression levels in A549^High-M^ cells treated with 1,6-hexanediol. *Biological replicates n* = *5, independent experiments, Unpaired t test (Two-tailed); Mean *± SD. **H** Establish a conditional culture medium experiment: Representative images of TRAP assay analyzing the osteoclast differentiation capacity induced by conditioned medium of A549 cells pre-treated with 1,6-hexanediol (*n* = 3, independent experiments), *p < *0.05*) (*Scale bar: 10 μm*). *Biological replicates n* = *3, Unpaired t test (Two-tailed); Mean *± SD. The schematic diagram was created by the author using Adobe Illustrator. **I** Representative images of the activation effect of A549^High-M^ cell conditioned medium on osteoclasts under IL6 neutralizing antibody (Siltuximab) treatment (*Scale bar: 200 μm*). *Biological replicates n* = *3, independent experiments, Unpaired t test (Two-tailed)*; *Mean *± SD. The schematic diagram was created by the author using Adobe Illustrator.

In order to identify the downstream effectors that lead to bone metastasis, we performed RNA-seq profiling on A549^Low-M^ and A549^High-M^ cells. This analysis identified *IL6* and *CCL5* among the top 15 most differentially expressed genes (Supplementary Fig. S2A, B). Knockdown of YBX1 in A549^High-M^ cells significantly reduced *IL6* and *CCL5* expression (Supplementary Fig. S2C). We analyzed the expression levels of IL6 and CCL5 in clinical specimens and their correlation with YBX1 expression. The results confirmed that IL6 and CCL5 were significantly lower in serum from non-metastatic patients compared with serum from metastatic patients (Supplementary Fig. S2D, E); and positive correlated with YBX1 expression in clinical bone metastasis cases (Supplementary Fig. S2F, G). Consistent with their increase in cancer cells and clinical samples, elevated serum IL6 and CCL5 levels were also observed in mice bearing A549^High-M^ xenografts (Fig. 3A). To investigate the direct transcriptional regulation of *IL6* and *CCL5* by YBX1, we performed ChIP-seq analysis using the YBX1-specific antibody. Sequencing results revealed significant enrichment of YBX1 on DNA fragments of *IL6* and *CCL5* (Supplementary Fig. S3A–C). Furthermore, these findings were validated through luciferase reporter assays and ChIP. Consistent results demonstrated that YBX1 overexpression enhances the transcriptional activity of *IL6* and *CCL5* by directly binding to their respective promoter regions. (Fig. 3B and Supplementary Fig. S3D).

Prior studies suggest that YBX1 undergoes Liquid-Liquid Phase Separation (LLPS) in cancer cells, and its activity highly relies on liquid-like condensates^13^. To investigate whether LLPS mediates YBX1’s role in bone metastasis, we compared condensate formation between A549^Low-M^ and A549^High-M^ cells. A549^High-M^ cells exhibited significantly increased YBX1 condensates in the nucleus, which were disrupted by 5% 1,6-hexanediol that is commonly used to dissolve LLPS (Fig. 3C, D). Fluorescence recovery after photobleaching (FRAP) analysis further demonstrated rapid recovery (~seconds) of YBX1 condensates, confirming their dynamic LLPS properties (Fig. 3E). YBX1 condensates colocalized with active transcriptional markers, including phosphorylated RNA Pol II (Ser5) and H3K27ac (Fig. 3F). ChIP assays revealed H3K27ac enrichment at *IL6* and *CCL5* promoters (Supplementary Fig. S3E), suggesting active transcription within these condensates. Functional studies showed that 1,6-hexanediol (1,6Hex) pretreatment significantly reduced *IL6* and *CCL5* expression (Fig. 3G). Subsequently, we analyzed the effect of 1,6-Hex-treated A549 cells on osteoclast activation. The results showed that the conditioned medium from 1,6-Hex-treated A549 cells could weaken osteoclast activation ability (Fig. 3H). This effect was consistent with that of IL6-neutralizing antibody (Fig. 3I), indicating that the high expression of IL6 mediated by phase separation is a key factor through which A549 cells promote osteoclast activation. Inhibition of phase separation suppressed IL6 expression, thereby concurrently inhibiting the ability of A549 cells to activate osteoclasts.

### YBX1 orchestrates bone metastasis through the synergistic actions of IL6 and CCL5

**Fig. 4:**
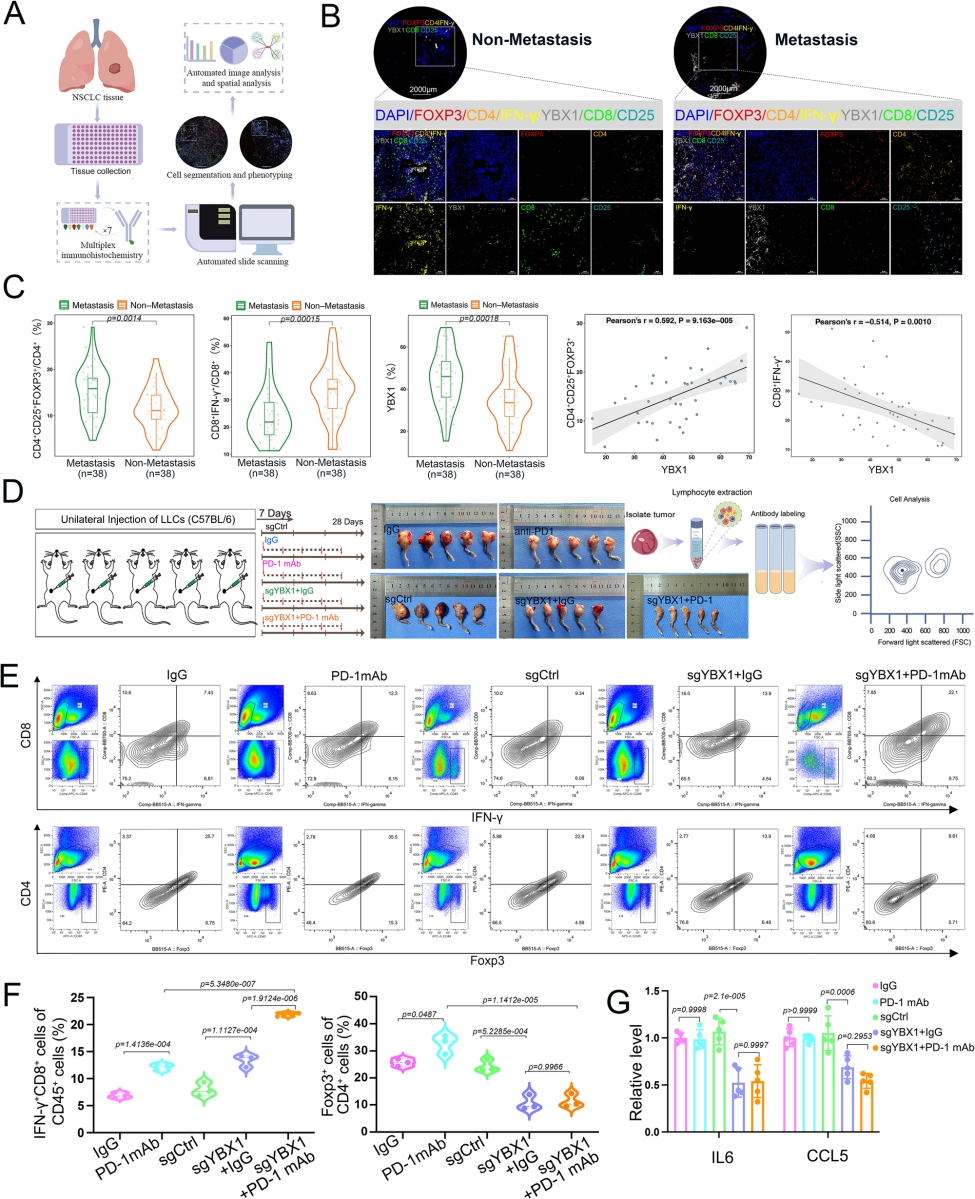
YBX1 Promotes an immunosuppressive microenvironment in non-small cell lung cancer bone metastasis by driving CCL5 Secretion. **A** Experimental flowchart: includes tissue collection, multiplex immunofluorescence staining, and automated scanning analysis. The schematic diagram was created by the author using Adobe Illustrator. **B** Representative multiplex immunofluorescence images showing the infiltration of immune cells in metastatic and non-metastatic tissues (*Scale bar: 2000 μm*). *Biological replicates n* = *38, patient samples*. **C** Statistical analysis shows the infiltration levels of Treg cells (CD4^+^CD25^+^FoxP3^+^) and IFN-γ^+^CD8^+^ T cells, as well as YBX1 expression levels, in primary tissues from patients without metastasis (*n* = *38*) and with bone metastasis (*n* = *38*). *Biological replicates, Unpaired t test (Two-tailed)*. *For Treg cells: Non-metastasis: n* = *38, patient samples, minima* = *2.6, maxima* = *22.3, center* = *11.1, Q1/Lower Quartile* = *9.225, Q3/Upper Quartile* = *14.45, Lower Whisker* = *2.6, Upper Whisker* = *22.2875, IQR/Interquartile Range* = *5.225; Metastasis: n* = *38, patient samples, minima* = *4.7, maxima* = *29.1, center* = *15.85, Q1/Lower Quartile* = *10.7, Q3/Upper Quartile* = *18.1, Lower Whisker* = *4.7, Upper Whisker* = *29.1, IQR / Interquartile Range* = *7.4; For IFN-γ*^*+*^*CD8*^*+*^ *T cells: Non-metastasis: n* = *38, patient samples, minima* = *11.8, maxima* = *56.5, center* = *34, Q1/Lower Quartile* = *26.825, Q3/Upper Quartile* = *37.575, Lower Whisker* = *11.8, Upper Whisker* = *53.7, IQR/Interquartile Range* = *10.75; Metastasis: n* = *38, patient samples, minima* = *11.3, maxima* = *51.2, center* = *21.85, Q1/Lower Quartile* = *17.225, Q3/Upper Quartile* = *28.975, Lower Whisker* = *11.3, Upper Whisker* = *46.6, IQR/Interquartile Range* = *11.75; For YBX1: Non-metastasis: n* = *38,patient samples, minima* = *10.8, maxima* = *65.2, center* = *33.5, Q1/Lower Quartile* = *26.95, Q3/Upper Quartile* = *39.75, Lower Whisker* = *10.8, Upper Whisker* = *58.95, IQR/Interquartile Range* = *12.8; Metastasis: n* = *38, patient samples, minima* = *15.3, maxima* = *69.2, center* = *45.95, Q1/Lower Quartile* = *36.15, Q3/Upper Quartile* = *53.075, Lower Whisker* = *15.3, Upper Whisker* = *69.2, IQR/Interquartile Range* = *16.925*. The correlation between YBX1 expression and Treg cell infiltration, as well as IFN-γ^+^CD8⁺ T cell infiltration level. *Biological replicates n* = *38, patient samples, Pearson’s correlation test (Two-tailed)*. **D** Schematic diagram of mouse model construction and experimental treatments (including sgRNA-mediated YBX1 knockout and immune checkpoint inhibitor therapy). Treatment: IgG/PD-1 mAb 200 µg/mouse (Intraperitoneal injection), ICA 35 mg/kg/day (Intragastric administration). The schematic diagram was created by the author using Adobe Illustrator. **E**, **F** Representative flow cytometry plots (**E**) and quantitative analysis (**F**) of the infiltration of IFN-γ^+^CD8^+^ T cells and Treg in the tumor microenvironment of bone metastatic lesions. *Biological replicates n* = *3, mice; One-way ANOVA (multivariate comparison: Tukey)*. **G** Relative levels of IL6 and CCL5 in the serum of mice from different treatment groups. (*n* = *5, mice*). *Biological replicates n* = *5, mice; One-way ANOVA (multivariate comparison: Tukey); Mean *± SD.

During clinical practice, primary tumors from NSCLC patients with bone metastasis often exhibit an immunologically “cold” state. CCL5 signals through canonical receptors (CCR5, CCR1, CCR3) and non-canonical receptors (ACKR1, GPR75), with CCR5 being its primary receptor. Prior studies indicate that tumor-derived CCL5 recruits regulatory T cells (Tregs) via CCR5 to suppress antitumor immunity. We thus hypothesized that YBX1-driven CCL5 secretion might contribute to this immunosuppressive phenotype. To explore this mechanism, we analyzed Treg infiltration (using FOXP3 as a Treg marker) and YBX1 expression in primary tumors from bone metastasis-positive versus metastasis-free patients. As anticipated, YBX1 levels were higher in metastatic tissues compared to non-metastatic tissues, accompanied by elevated Treg infiltration and reduced CD8⁺ T cell infiltration. YBX1 expression showed a positive correlation with Treg abundance and an inverse correlation with CD8⁺ T cell levels (Fig. 4A–C). Furthermore, we established bone metastasis models, combined with immune checkpoint inhibitor therapy. The results revealed that depletion of YBX1 by small guide (sg) RNA significantly increased CD8^+^ T cell and reduced Treg cell infiltration by regulating the level of CCL5/IL6 in LLC tumors at the bone metastasis site (Fig. 4D–G). These findings suggest that YBX1 overexpression promotes an immunosuppressive niche in bone-metastatic NSCLC, potentially through CCL5-CCR5-mediated Treg recruitment.

IL6, a well-established mediator of tumor bone metastasis, promotes osteolytic lesions through multiple mechanisms, including RANKL/RANK pathway activation, induction of matrix metalloproteinases (MMPs), and vascular endothelial growth factor (VEGF) upregulation. We further investigated IL6’s role in YBX1-mediated bone metastasis. In vivo, IL6 neutralizing antibody treatment markedly reduced metastatic burden in YBX1-driven bone metastasis models (Supplementary Fig. S4A–C); but show no difference in the level of CD8 (Supplementary Fig. S4D). These results indicate that YBX1 promotes bone metastasis through the coordinated actions of CCL5 (driving immunosuppression) and IL6 (activating osteoclasts and tumor-stroma crosstalk).

### O-GlcNAcylation at T271 redirects YBX1 to mitochondria, impairs its nuclear transcriptional activity in bone metastasis

**Fig. 5:**
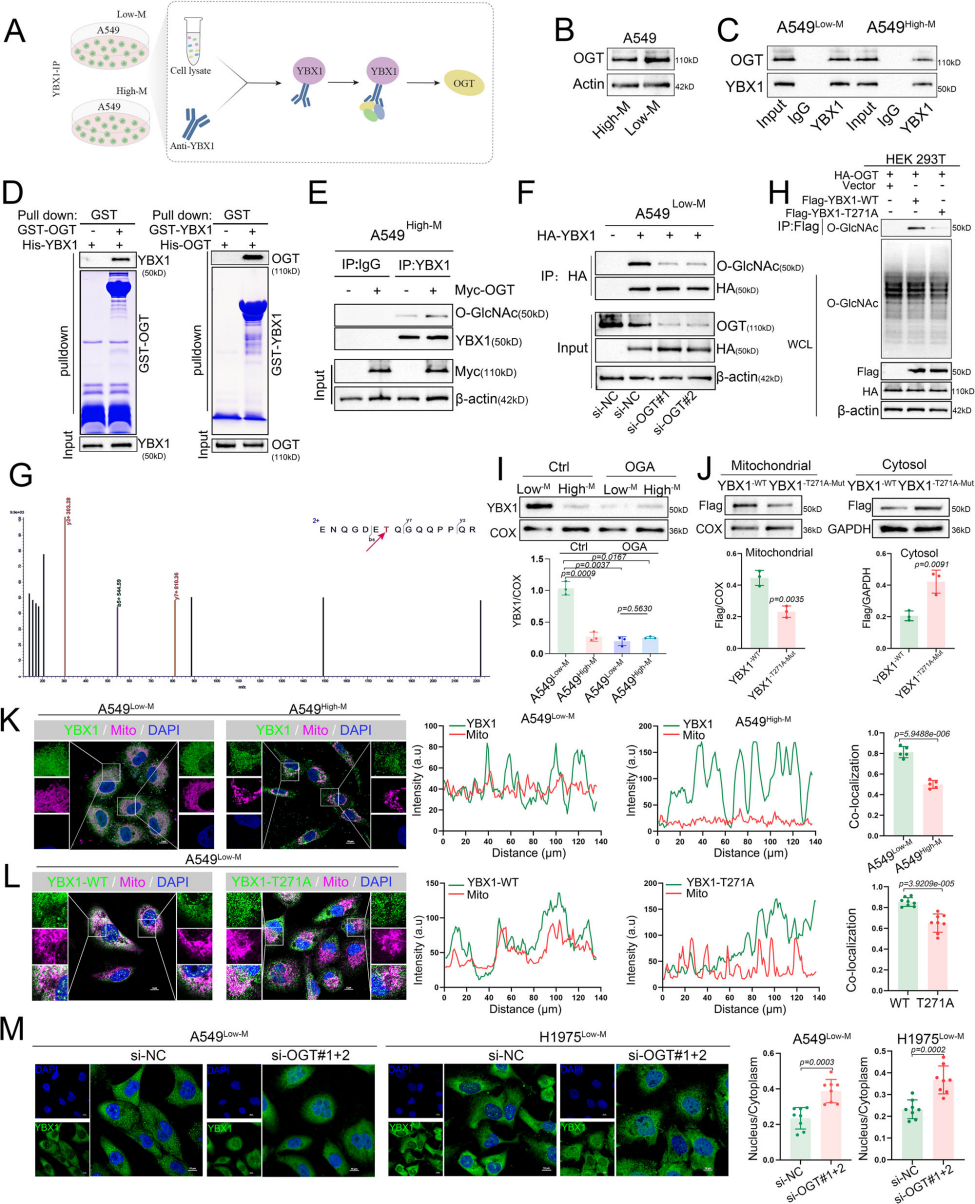
Mechanism by which O-GlcNAcylation of YBX1 regulates its subcellular localization to influence metastatic potential. **A** YBX1 was found to specifically interact with O-GlcNAc transferase (OGT) in low-metastatic potential cells (A549^Low-M^) identified by co-immunoprecipitation-mass spectrometry (IP-mass spectrometry). The schematic diagram was created by the author using Adobe Illustrator. **B** Representative western blot images demonstrating OGT protein levels in A549^Low-M^ and A549^High-M^ cells. *Biological replicates n* = *3 (independent experiments)*. **C** Representative co-immunoprecipitation (Co-IP) assay images demonstrating the interaction between OGT and YBX1 in A549^Low-M^ and A549^High-M^ cells. *Biological replicates n* = *3 (independent experiments)*. **D** Representative GST-pull-down assay images confirming a direct interaction between OGT and YBX1. *Biological replicates n* = *3 (independent experiments)*.** E**, **F** Representative western blot images demonstrating the effect of OGT overexpression (E) in A549^High-M^ cells and OGT knockdown (F) in A549^Low-M^ cells on the O-GlcNAcylation of YBX1. *Biological replicates n* = *3 (independent experiments)*. **G** Mass spectrometry analysis identifying T271 on YBX1 as a potential O-GlcNAcylation site. **H** A Flag-YBX1-T271A point mutation plasmid was constructed. Representative Co-IP assay images demonstrating the glycosylation level of YBX1 after the T271A mutation. *Biological replicates n* = *3 (independent experiments)*. **I** Mitochondrial fractionation combined with Western blot analysis of the mitochondrial/cytoplasmic YBX1 ratio in high- and low-metastatic cells and the change in this ratio after OGA treatment. *Biological replicates n* = *3 (independent experiments); Tukey’s multiple comparisons test; Mean *± SD. **J** Western blot analysis of the mitochondrial/cytoplasmic YBX1 ratio after overexpression of the YBX1^T271A-Mut^ (right). *Biological replicates n* = *3 (independent experiments); Paired t test (Two-tailed); Mean *± SD. **K**, **L** Representative images of immunofluorescence co-localization assay (DAPI for nuclei, YBX1 antibody-green signal, mitochondrial marker-red signal) analyzing the co-localization level of YBX1 with mitochondria in low- and high-metastatic cells (**K**); *Biological replicates n* = *5 (independent experiments); Unpaired t test (Two-tailed); Mean *± SD*;* and the co-localization level of YBX1 with mitochondria after the T271 mutation (**L**); *Biological replicates n* = *8 (independent experiments); Unpaired t test (Two-tailed); Mean *± SD. (*Scale bar: 10 μm*). **M** Representative images of immunofluorescence analysis of the effect of OGT knockdown in low-metastatic potential cells on the nuclear/cytoplasmic localization of YBX1. *Biological replicates n* = *8 (independent experiments); Unpaired t test (Two-tailed); Mean *± SD*;* (*Scale bar: 10 μm*).

As a transcription factor, the activity of YBX1 depends on its nuclear localization. Interestingly, we found that the nuclear-to-cytoplasmic ratio of YBX1, which governs its transcriptional regulation of CCL5 and IL6, was significantly elevated in metastatic tissues (Supplementary Fig. S5A) and high-metastatic A549^High-M^ cells (Supplementary Fig. S5B). To investigate the mechanism underlying this difference, IP-mass spectrometry was performed to identify the binding partners of YBX1, and the result revealed that O-GlcNAc transferase (OGT), a glycosylation enzyme, bound with YBX1 exclusively in A549^Low-M^ cells (Fig. 5A). Western blot assay proved that the protein level of OGT was higher in A549^Low-M^ cells than that in A549^High-M^ cells (Fig. 5B). Co-IP assays confirmed stronger OGT-YBX1 interaction in A549^Low-M^ versus A549^High-M^ cells (Fig. 5C). GST pull-down assay further confirmed direct interaction between OGT and YBX1 using purified proteins (Fig. 5D), suggesting that YBX1 may undergo O-GlcNAcylation in A549^Low-M^ cells. Furthermore, we analyzed the level of OGT in clinical tissue samples. The results showed that the expression of OGT was significantly lower in metastatic tissues compared to non-metastatic tissues (Supplementary Fig. S5C). And high OGT expression indicates a better survival prognosis (Supplementary Fig. S5D). Moreover, in both metastatic and non-metastatic tissues, OGT expression is negatively correlated with YBX1 protein level (Supplementary Fig. S5E). To test whether interaction of OGT on YBX1 results in its O-GlcNAcylation, myc-tagged OGT was overexpressed in A549^High-M^ cells, the following analysis of YBX1 O-GlcNAcylation, as shown in Fig. 5E, F, OGT overexpression significantly increased YBX1 O-GlcNAcylation, while, OGT knockdown in A549^Low-M^ cells suppressed. In addition, Mass spectrometry further identified T271 as a putative O-GlcNAcylation site (Fig. 5G). T271A mutation reduced YBX1 glycosylation (Fig. 5H), confirming T271 as the critical modification site.

Given the established link between protein glycosylation and membrane organelle localization^14^, we assessed whether T271 O-GlcNAcylation redirects YBX1 to mitochondria. Mitochondria isolated from high- and low-metastatic cells were subjected to Western blot analysis, with the inclusion of OGA. The results demonstrated that YBX1 levels in mitochondria from high-metastatic cells were significantly lower than those from low-metastatic cells (Fig. 5I); while following OGA treatment or transfected with YBX1^T271A-mut^, mitochondrial YBX1 in low-metastatic cells was also reduced, confirming that glycosylation modification promotes mitochondrial localization of YBX1 (Fig. 5J). Immunofluorescence co-localization assays also demonstrated a higher mitochondrial content of YBX1 in the low-metastatic cells compared to the high-metastatic cells, and the phenomenon in low-metastatic cells was abolished upon T271 site mutation (Fig. 5K, L and Supplementary Fig. S6A, B), demonstrating that O-GlcNAcylation at T271 drives YBX1 mitochondrial translocation. Further analysis of the nucleo-cytoplasmic ratio of YBX1 after knockdown of the glycosyltransferase OGT revealed that loss of OGT increased the nuclear-to-cytoplasmic ratio of YBX1 (Fig. 5M), indicating that removal of O-GlcNAcylation promotes YBX1 translocation into the nucleus, where it may exert its transcriptional regulatory function.

To elucidate the impact of glycosylation at the T271 site of YBX1 on bone metastasis, we established a bone metastasis animal model. We analyzed the effect of the T271 site mutation on bone metastasis, combined with the OGA inhibitor Thiamet G (OGA-i) to assess how glycosylation levels at T271 influence metastasis. The results showed that both wild-type and T271-mutant vectors could rescue the effect of YBX1 knockdown. However, the rescue effect of the wild-type vector was abolished when combined with the OGA-i, whereas the rescue effect of the mutant vector was not inhibited by the OGA-i (Supplementary Fig. S7). These findings indicate that glycosylation at the T271 site is critical for YBX1-mediated bone metastasis.

### Small-molecule compound ICA suppresses YBX1 via the autophagic degradation pathway

**Fig. 6:**
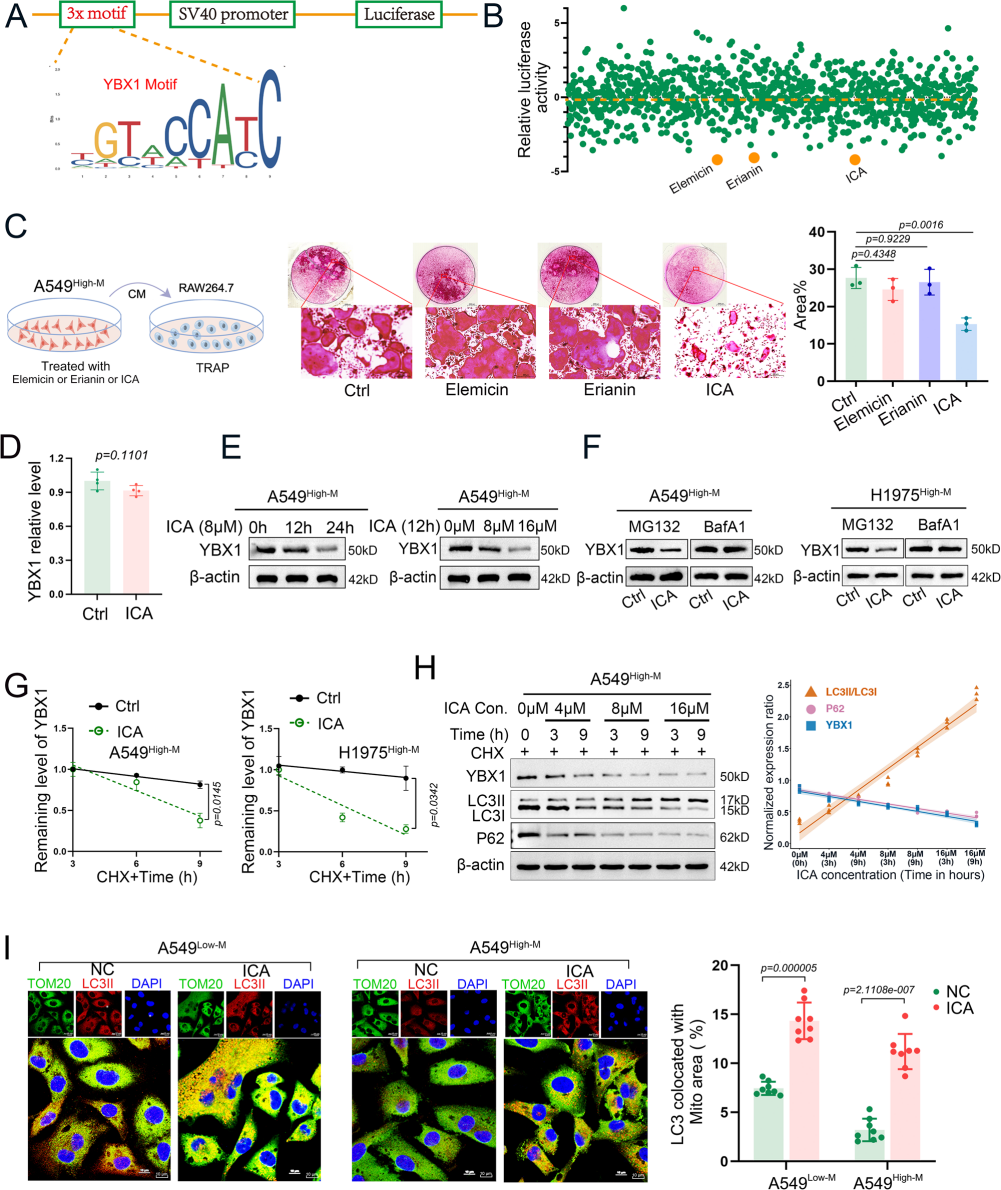
Screening of small-molecule compounds and the regulatory mechanism of ICA on YBX1. **A** Schematic diagram of the luciferase reporter system based on the YBX1 DNA-binding motif, used for screening compounds targeting YBX1. **B** Signal values after compound treatment, showing that three candidate compounds (Elemicin, Erianin, and ICA) significantly inhibited YBX1 transcriptional activity, with ICA exhibiting the most pronounced inhibitory effect. **C** Representative images of TRAP staining analysis of the effect of conditioned medium of A549^High-M^ cells treated with Elemicin, Erianin, ICA on osteoclast activation. (*Scale bar: 200 μm*). *Biological replicates n* = *3, independent experiments; One-way ANOVA (multivariate comparison: Dunnett); Mean *± SD. The schematic diagram was created by the author using Adobe Illustrator. **D** RT-qPCR analysis of the effect of ICA on YBX1 mRNA expression. *Biological replicates n* = *4; Unpaired t test (Two-tailed); Mean *± SD. **E** Representative images demonstrating the effect of ICA treatment on YBX1 protein levels in A549^High-M^ cells (different time points 0 h, 12 h, 24 h, dose 8 µM and ICA doses 0 µM, 8 µM, 16 µM). *Biological replicates n = 3 (independent experiments)*. **F** Investigation of the pathway by which ICA promotes YBX1 degradation using the proteasome inhibitor MG132 and the autophagy inhibitor BafA1. *Biological replicates n* = *3 (independent experiments)*. **G** Detect the affection of ICA on the degradation ability of YBX1 at different time points with treatment of CHX. *Biological replicates n* = *3 (independent experiments); Repeated measures Two-way ANOVA ((multivariate comparison:Bonferroni); Mean *± SD. **H** Representative western blot images demonstrating YBX1, LC3II, and P62 protein levels in ICA-treated cells at different time (0 h, 3 h, 9 h) or dose (0 µM, 4 µM, 8 µM, 16 µM). *Biological replicates n* = *3 (independent experiments). The shaded area indicates the 95% confidence interval for the linear fit*. **I** Representative images of immunofluorescence analysis of mitophagy after ICA treatment. (*Scale bar: 10 μm*). *Biological replicates n* = *8 (independent experiments); Unpaired t test (Two-tailed); Mean *± SD.

To identify small-molecule compounds targeting YBX1, we screened a chemical library using a luciferase reporter system based on the conserved YBX1 DNA-binding motif in 293 T cells. This screen identified three candidate compounds that significantly inhibited YBX1 transcriptional activity (Fig. 6A, B). Among them, the supernatant from ICA-treated tumor cells demonstrated the most significant inhibition of osteoclast activation (Fig. 6C). Then, what is the primary mechanism by which ICA affects YBX1? We analyzed the IC50 of four cell lines (A549^High-M^, A549^Low-M^, H1975^High-M^, H1975^Low-M^) to ICA (Supplementary Fig. S8A) and the effect of ICA on YBX1 mRNA and protein levels. The results showed that ICA did not affect YBX1 mRNA levels (Fig. 6D) but suppressed YBX1 protein levels in a time- and dose-dependent manner (Fig. 6E and Supplementary Fig. S8B, C), especially nucleus level (Supplementary Fig. S8D).

Protein degradation primarily involves the ubiquitin-proteasome pathway and the autolysosome pathway. To clarify the specific regulatory route of ICA on YBX1 protein, we used the proteasome inhibitor MG132 and the autolysosome formation inhibitor BafA1. The results indicated that ICA functions mainly through the autolysosome pathway (Fig. 6F). Treatment with the protein synthesis inhibitor CHX revealed that ICA promotes the degradation of YBX1 (Fig. 6G). Furthermore, we observed a negative correlation between YBX1 levels and the degree of autophagy (Fig. 6H). Subsequently, we found that mitophagy occurs in both low- and high-metastatic cells, and ICA can exacerbate mitophagy (Fig. 6I and Supplementary Fig. S8E). Moreover, we found that treatment with an OGA inhibitor mimicked the effect of ICA in promoting YBX1 degradation (Supplementary Fig. S8F). Based on this observation and our prior finding that glycosylated YBX1 localizes to mitochondria, we therefore ask: does ICA-induced glycosylation promote the mitochondrial localization of YBX1, leading to its degradation via mitophagy?

### ICA targets YBX1 O-GlcNAcylation to induce mitochondrial translocation and mitophagic degradation

**Fig. 7:**
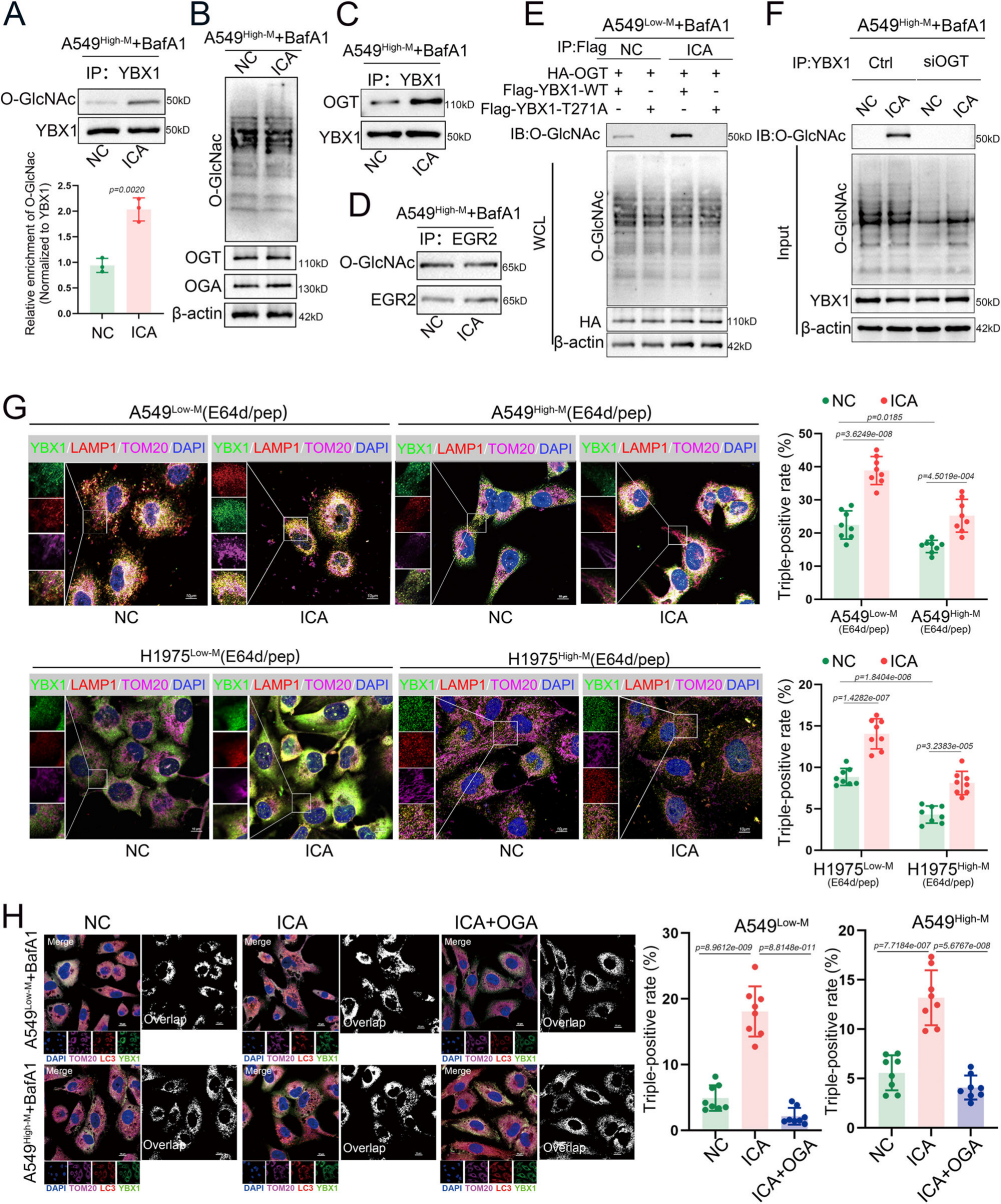
ICA promotes the O-GlcNAcylation modification of YBX1 by enhancing its interaction with OGT, thereby driving the mitophagic clearance of YBX1. **A**–**D** Co-immunoprecipitation assays analyzing the effect of ICA on the binding levels between YBX1 and O-GlcNAc as well as OGT (**A**, **C**); the effect of ICA on global O-GlcNAcylation levels, and OGT or OGA expression (**B**); the effect of ICA on the O-GlcNAcylation level of EGR2, a known O-GlcNAcylated protein (**D**). *Biological replicates n* = *3 (independent experiments); Paired t test (Two-tailed); Mean *± SD. **E** Representative Co-IP assay images demonstrating the changes in ICA-induced glycosylation of YBX1 after mutation at its T271 site. *Biological replicates n* = *3 (independent experiments)*. **F** Representative Co-IP assay images demonstrating the analysis of the effect of ICA on YBX1 O-GlcNAcylation after OGT knockdown. *Biological replicates n* = *3 (independent experiments)*. **G** Representative images of immunofluorescence analysis of the co-localization of YBX1 with mitochondria (marked by TOM20) and lysosomes (marked by LAMP1) after ICA treatment. (*Scale bar: 10 μm*). *Biological replicates n* = *8 (independent experiments); Two-way ANOVA (multivariate comparison:Bonferroni); Mean *± SD.** H** Representative images of immunofluorescence analysis of YBX1 and mitochondria recruitment into autophagosomes after ICA treatment, and the effect of OGA treatment on the recruitment of YBX1 to autophagosomes. (*Scale bar: 10 μm*). *Biological replicates n* = *8 (independent experiments); One-way ANOVA (multivariate comparison:Dunnett); Mean *± SD.

To validate our hypothesis, we employed fluorescence co-localization assays to analyze whether ICA treatment affects the glycosylation modification of YBX1. As anticipated, co-immunoprecipitation experiments confirmed that ICA promotes the glycosylation modification of YBX1 without affecting global glycosylation levels, OGT or OGA levels, but enhanced the binding affinity between YBX1 and OGT (Fig. 7A–C), while failed to enhance glycosylation of the reported target EGR2^15^(Fig. 7D). These findings suggest that ICA acts by specifically enhancing the interaction of YBX1 with OGT. As we previously demonstrated, the T271 site of YBX1 is critical for its glycosylation-mediated mitochondrial translocation. Therefore, we introduced a T271 site mutation and re-examined the effect of ICA on YBX1 modification. The results showed that mutation of the T271 site in YBX1 markedly attenuated the enhancing effect of ICA on its glycosylation (Fig. 7E), but was abolished upon OGT knockdown (Fig. 7F).

Next, to investigate whether YBX1 enters mitophagy under ICA treatment, we analyzed the effects of ICA on the co-localization of YBX1 with mitochondria (labeled by TOM20) and lysosomes (labeled by LAMP1) in both high- and low-metastatic cells. The results showed that the co-localization levels of YBX1 with mitochondria and lysosomes were higher in low-metastatic cells than in high-metastatic cells. Moreover, ICA promoted the co-localization of YBX1 with both mitochondria and lysosomes in both high- and low-metastatic cells (Fig. 7G). In addition, we examined the localization of ICA-treated YBX1 and mitochondria in autophagosomes. The results demonstrated that ICA promotes the entry of YBX1 and mitochondria into autophagosomes. Furthermore, the level of YBX1 recruitment into autophagosomes could be inhibited by OGA (Fig. 7H). This indicates that ICA drives YBX1 mitochondrial translocation, followed by mitophagic clearance.

### The T271 site of YBX1 is a critical regulatory point for ICA-mediated suppression of bone metastasis and amelioration of the immunosuppressive tumor microenvironment

**Fig. 8:**
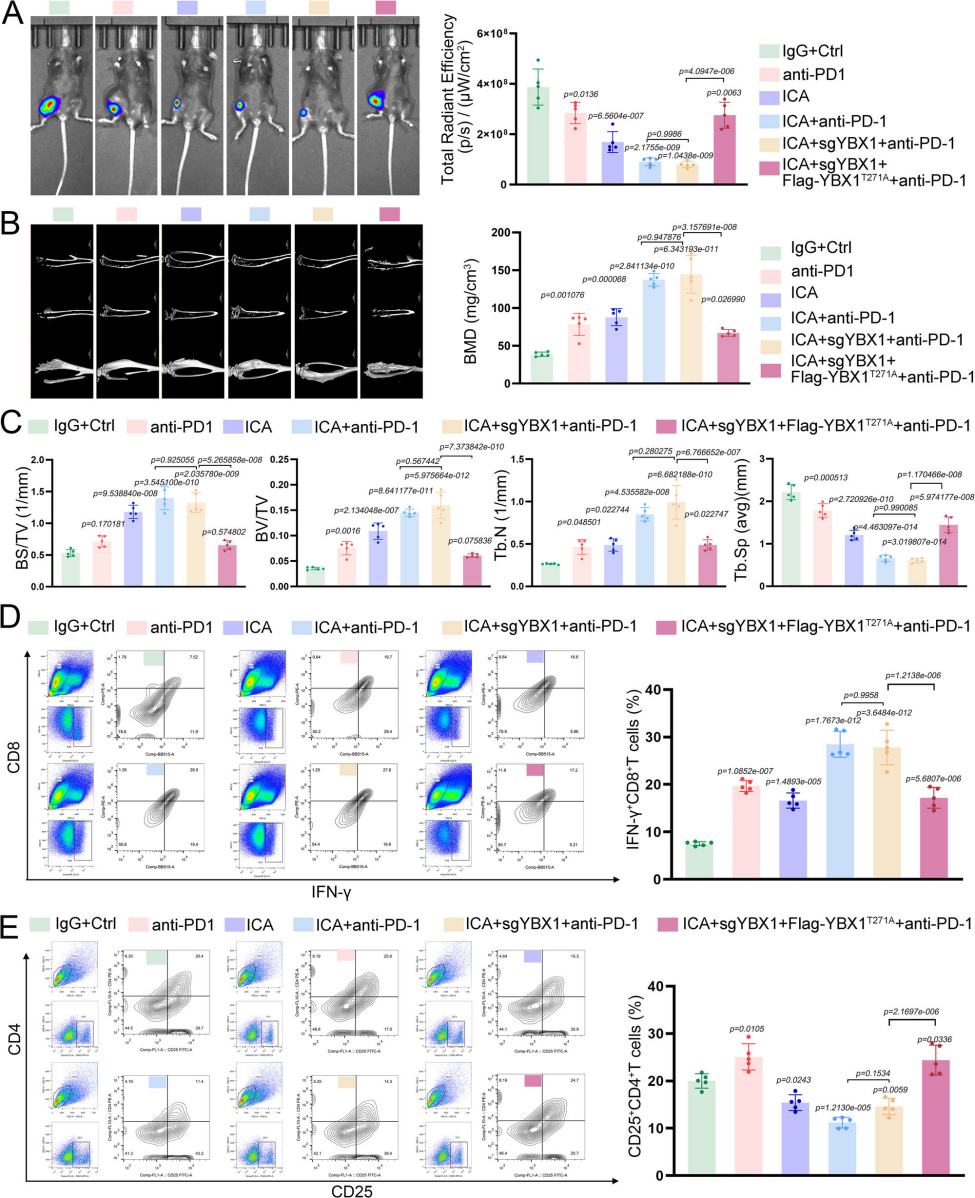
The therapeutic potential of ICA as a YBX1-targeting drug and the impact of the YBX1 T271 site mutation on the efficacy of ICA. **A** In vivo imaging of mice showing the intensity of bone metastasis radiance signals in different treatment groups (representative images). Treatment: IgG/PD-1mAb 200 µg/mouse (Intraperitoneal injection), ICA 35 mg/kg/day (Intragastric administration). *Biological replicates n* = *5, mice; One-way ANOVA (multivariate comparison:Tukey); Mean *± SD. **B** Representative microCT images of mouse bone and a histogram of quantitative analysis of bone mineral density (BMD). *Biological replicates n* = *5, mice; One-way ANOVA (multivariate comparison:Tukey); Mean *± SD. **C** Histogram comparison of bone histomorphometric parameters: Bone Surface/Tissue Volume (BS/TV), Bone Volume/Tissue Volume (BV/TV), Trabecular Number (Tb.N), and Trabecular Separation (Tb.Sp). *Biological replicates n* = *5, mice; One-way ANOVA (multivariate comparison:Tukey); Mean *± SD. **D**, **E** Flow cytometry analysis of the percentage of IFN-γ^+^CD8^+^ T cells and Treg. *Biological replicates n* = *5; One-way ANOVA (multivariate comparison:Tukey); Mean *± SD.

To evaluate the therapeutic potential of ICA as a YBX1-targeted agent and the influence of the YBX1 T271 site mutation on ICA’s efficacy, we established a bone metastasis model with the following treatment groups: ICA alone, immune checkpoint blockade (ICB) alone, their combination, with or without sgYBX1 or YBX1-T271A mutant. The results showed that either ICA or PD-1 inhibitor alone suppressed bone metastasis, while their combination exhibited enhanced efficacy, and sgYBX1 did not further potentiate this effect, validating that the efficacy of ICA is dependent on YBX1. However, introducing the YBX1-T271A mutation abolished the inhibitory effect of ICA (Fig. 8A–C). Flow cytometry analysis revealed that the combination therapy significantly increased the proportion of activated CD8^+^ T cells and decreased the proportion of Treg, but this immune-sensitizing effect of ICA was lost upon overexpression of YBX1-T271A (Fig. 8D, E). These results indicate that the immune-sensitizing effect of ICA depends on glycosylation modification at the T271 site of YBX1.

## Discussion

Bone metastasis (BM) occurs in over 50% of non-small cell lung cancer (NSCLC) patients during disease progression, leading to severe skeletal-related events (SREs) such as pathological fractures, spinal compression, and hypercalcemia, which drastically impair quality of life and prognosis. The median survival post-BM diagnosis remains poor at 6–10 months, underscoring the urgent need for effective management strategies. Despite progress, BM management faces hurdles such as treatment resistance and limited biomarkers. Research into the bone microenvironment’s role in immune evasion and metastasis is critical. In the presenting study, we delve into the mechanisms by which YBX1 influences bone metastasis in non-small cell lung cancer (NSCLC) and its impact on the immune microenvironment, revealing the unique role of O-GlcNAc glycosylation in regulating YBX1 function. These findings provide new targets and theoretical bases for precision therapy in lung cancer with bone metastasis.

By constructing high-metastatic NSCLC cell line models, we found that high expression of YBX1 is significantly associated with bone metastasis capability, and interference with YBX1 effectively inhibits the occurrence of bone metastasis. Further investigations using Western blotting and immunofluorescence experiments showed that O-GlcNAc glycosylation at the T271 site significantly reduced the nuclear localization of YBX1, thereby decreasing its transcriptional activation of IL6 and CCL5. Specifically, IL6 activates the RANKL/RANK pathway to mediate osteoclast activation, a critical feature of bone metastasis^16^, whereas CCL5 primarily affects the recruitment of immune cells^17,18^. Unlike previous studies that mainly focused on YBX1’s role as a conventional transcription factor and its effects on downstream genes^19^, our study elucidates the specific pathways by which YBX1 activates IL6 and CCL5 through liquid-liquid phase separation (LLPS), providing new insights into lung cancer bone metastasis. Previous research has shown that YBX1’s phase separation can have different functions depending on the molecular context^20^. However, unlike existing studies, our research focuses on how YBX1 specifically regulates the expression of pro-metastatic genes (such as IL6 and CCL5) via LLPS, simultaneously modulating the invasiveness and migratory properties of tumor cells and reshaping the tumor microenvironment. Although both involve YBX1’s phase separation characteristics, our study emphasizes YBX1’s pivotal role under specific pathological conditions (such as lung cancer bone metastasis), particularly in reshaping the tumor microenvironment while regulating tumor cell invasiveness and migration. Moreover, most existing studies concentrate on the invasiveness and migratory properties of tumor cells themselves^21^, while our study also highlights YBX1’s crucial role in remodeling the bone metastasis microenvironment, underscoring the importance of its dynamic regulatory process. Clinical specimen analysis confirmed the relationship between YBX1, CCL5, and IL6, as well as their adverse expression and patient prognosis. Furthermore, our cohort analysis confirmed that high YBX1 expression is an independent risk factor for bone metastasis in NSCLC, and this association remains significant regardless of EGFR mutation status. Although EGFR mutations are associated with metastatic characteristics in NSCLC, our data demonstrate that high YBX1 expression predicts poorer bone metastasis-free survival in both EGFR-mutant and wild-type patients. This suggests that the bone metastasis pathway driven by YBX1 may operate independently of the canonical EGFR signaling axis. Targeting YBX1 could therefore represent a potential therapeutic strategy for bone metastasis patients with EGFR-TKI resistance or those harboring wild-type EGFR.

YBX1 plays a significant role in regulating the tumor immune microenvironment. Our study shows that YBX1 enhances the recruitment of Treg cells by upregulating CCL5 expression, thereby suppressing anti-tumor immune responses and leading to the formation of a “cold” tumor microenvironment^22^. Flow cytometry analysis indicated that interfering with YBX1 significantly increased the proportion of CD8^+^ T cells while reducing the proportion of Treg cells, suggesting that YBX1 could be a potential target for reversing immune suppression and enhancing the sensitivity of immunotherapy. As a multifunctional protein, YBX1 has broad regulatory roles in cell biology, including significant impacts on immune regulation, cellular senescence, and disease progression^23^. It maintains immune system homeostasis by controlling the function of immune cells and the expression of immune molecules, influencing various disease pathologies, including tumors. Despite this, few studies have explored the specific mechanisms by which YBX1 regulates the immune microenvironment. Previous research has shown that transcription factors such as NF-κB and STAT3 play important roles in regulating tumor immune microenvironments^24–26^, but these studies often focus on inflammatory factors and immune checkpoint molecules. Our study reveals that YBX1 regulates Treg cell recruitment and anti-tumor immune responses through CCL5 modulation, and we found that interfering with YBX1 significantly improves the efficacy of immunotherapy. This provides theoretical support for developing YBX1-targeted immunotherapy strategies. Given YBX1’s multiple functions in the immune system, further investigation into its specific mechanisms under different pathological conditions will aid in developing innovative therapeutic strategies, offering new insights and methods for the prevention, diagnosis, and treatment of related diseases.

Regarding the regulation of YBX1, our presenting study highlights the importance of O-GlcNAc glycosylation. We discovered that O-GlcNAc glycosylation at specific amino acid sites (such as T271) guides YBX1 from the nucleus to mitochondria and promotes YBX1 degradation via mitophagy. Western blotting and immunofluorescence experiments confirmed the significant impact of T271 glycosylation on YBX1’s subcellular localization and stability. While previous studies have shown that phosphorylation and acetylation modifications play crucial roles in regulating YBX1 function^27,28^, these studies primarily focused on YBX1’s influence on cell signaling, nuclear localization, and gene transcription. In contrast, our study reveals the unique role of O-GlcNAc glycosylation in regulating YBX1 function. The small molecule compound ICA can specifically enhance YBX1’s O-GlcNAc glycosylation, consequently increase its mitochondrial localization and mitophagy-mediated degradation, providing experimental evidence for developing new therapies targeting YBX1 and demonstrating the potential of O-GlcNAc glycosylation as a potential regulatory strategy.

Moreover, we discovered that the small-molecule compound ICA specifically enhances O-GlcNAcylation of YBX1, thereby promoting its mitochondrial localization and subsequent degradation via mitophagy, which in turn regulates osteoclast activation. Our findings are consistent with and extend previous research. Zhao et al. (Anticancer Drugs, 2020^29^) demonstrated that ICA inhibits lung cancer-mediated osteoclastogenesis and induces osteoclast apoptosis through the AMPK/mTOR signaling pathway, while also downregulating IL6 and TNF-α levels. Our study, more importantly, reveals its direct target in tumor cells—YBX1. We found that ICA promotes O-GlcNAcylation and subsequent degradation of YBX1, thereby suppressing the production of pro-metastatic factors such as IL6 at the transcriptional level. This provides an upstream mechanistic explanation for the decreased IL6 levels observed by Zhao et al., extending ICA’s mechanism of action from modulation of the tumor microenvironment to fundamental reversal of the “metastatic reprogramming” of tumor cells. Furthermore, our in vivo experiments confirmed the inhibitory effect of ICA on bone metastasis and its ability to sensitize tumors to immunotherapy. Thus, our study provides experimental evidence for developing YBX1-targeted therapies and highlights the potential of O-GlcNAcylation as a new regulatory strategy.

Despite the significant findings of presenting study regarding YBX1’s role in lung cancer bone metastasis and the formation of an immunosuppressive microenvironment, and the proposed O-GlcNAc glycosylation-based therapeutic strategy, there are still some limitations. Firstly, the study primarily relies on in vitro experiments and animal models. Although clinical specimen data support the relationship between YBX1, CCL5, and IL6 and their adverse expression and patient prognosis, the translational validity of these findings requires further validation. Secondly, although we identified a unique mechanism by which YBX1 regulates its function through O-GlcNAc glycosylation, whether this mechanism applies to other cancer types remains to be explored. Future research should validate the role of YBX1 in different cancer types using more clinical samples and assess the safety and efficacy of ICA-based small molecule drugs in clinical settings. Furthermore, investigating YBX1’s function and regulatory mechanisms under different pathological conditions may open new avenues for precision therapy in lung cancer and other cancers.

In conclusion, this study not only deepens our understanding of YBX1’s role in lung cancer bone metastasis and immune microenvironment formation but also highlights the importance of O-GlcNAc glycosylation as a unique regulatory mechanism, providing new directions for future research and therapeutic strategies. These findings are expected to advance personalized medicine for lung cancer and offer new approaches for improving patient outcomes. Future work will focus on validating these mechanisms in clinical practice to bring better treatment options to lung cancer patients.

## Methods

### Ethics Approval

This project was approved by the Institution Review Board of Hospital. Informed consent was obtained from each participant before the operation. The use of clinical specimens was completely in compliance with the “Declaration of Helsinki”. All animal experimental protocols were approved by the Ethics Committee of Nanjing Clinical Medical College, Nanjing University of Chinese Medicine (No. KY20180803-05). And all the animal procedures followed the Guide for the Care and Use of Laboratory Animals.

The animal experiments were conducted in accordance with protocols approved by the Nanjing University of Chinese Medicine. Institutional Animal Care and Use Committee (IACUC) (Protocol No.KY20180803-05), which strictly adheres to international standards, including the Guide for the Care and Use of Laboratory Animals, 8th Ed. (NRC, 2011) and the ARRIVE Guidelines 2.0 (Percie du Sert et al., PLoS Bio, 2020). The approved protocol explicitly defines the maximum permissible tumor burden as a volume of 2000 mm³ or a single diameter of 20 mm. We confirm that this established limit was not exceeded for any animal during the study.

### Patient cohorts and sample collection

A total of 180 treatment-free non-small cell lung cancer (NSCLC) patients without metastasis at initial diagnosis were enrolled between August 2018 and December 2020. The follow-up duration spanned 60 months, from August 2018 to August 2024. Collected specimens included primary tumor tissues, whole blood samples, and histopathological slides. For comparison, a non-follow-up cohort was established, consisting of 80 lung adenocarcinoma patients with bone metastasis at initial diagnosis. These patients were recruited from January 2019 to December 2024, with the same specimen types collected (primary tumor tissues, whole blood samples, and histopathological slides). Tissue specimens were snap-frozen in liquid nitrogen within 30 minutes after resection. Blood samples were processed by centrifugation (3000 × *g*, 10 min, 4 °C) to separate serum, which was then stored at − 80 °C until analysis.

### Cell culture

Luci-H1975 (FuHeng: FH0183), Luci-A549 (RRID: CVCL_QZ79), Luci-Lewis, and 293 T (RRID: CVCL_0063) cells were obtained from the Cell Bank of the Chinese Academy of Sciences. Regular mycoplasma testing and STR profiling confirm the absence of contamination. The routine culture conditions for Luci-Lewis cells were DMEM medium (Gibco, C11995500BT), 10% fetal bovine serum (Gibco), and 1% penicillin-streptomycin (Gibco, 15140122) at 37 °C, 95% air, and 5% CO_2_. H1975 cells were cultured in RPMI 1640 medium (Gibco, C11875500BT), 10% fetal bovine serum, 1% Glutamax (Gibco, 35050061), 1% sodium pyruvate (Pricella, PB180422), and 1% penicillin-streptomycin. A549 cells were cultured in F-12K medium, 10% fetal bovine serum, and 1% penicillin-streptomycin at 37 °C. 293 T cells were cultured in DMEM medium, 10% fetal bovine serum, 1% Glutamax, 1% sodium pyruvate, and 1% penicillin-streptomycin.

### Establishment of high bone-metastatic potential cell lines

To generate cell lines with enhanced bone-metastatic capacity, an orthotopic implantation and in vivo selection strategy via intratibial injection of A549 cells into immunodeficient mice (BALB/c-nu) were employed. Metastatic lesions in bone were isolated, dissociated, and re-cultured after 4–6 weeks. Iterative in vivo selection: Cells derived from bone metastases were re-injected into secondary mice for 3 cycles to enrich for high-metastatic subpopulations (A549High-M/H1975High-M). Low-metastatic lines (A549Low-M/H1975Low-M) were maintained under standard culture conditions without in vivo selection. Transwell assay, bioluminescence imaging and micro-CT were employed for metastasis capacity validation.

### Tartrate-resistant acid phosphatase (TRAP) staining

Cells were harvested and fixed with cold acetone for 5 min, then air-dried at room temperature for 10 min. TRAP staining was performed using the Tartrate-Resistant Acid Phosphatase (TRAP) Staining Kit (Solarbio, G1492) according to the manufacturer’s instructions. Briefly, cell slides were fixed with 4% formaldehyde for 10 min, washed with PBS, and then incubated with TRAP staining working solution (containing naphthol AS-BI phosphate, fast red TR salt, and potassium sodium tartrate in acetate buffer, pH 5.0) at 37 °C in the dark for 30–60 min. The reaction was stopped by rinsing with distilled water, followed by hematoxylin counterstaining of nuclei for 1–2 min. After washing under running water, the slides were mounted with glycerol gelatin and observed under a light microscope. TRAP-positive cells displayed red/purple granular cytoplasmic deposits.

### Proteomics

Proteins were extracted from each sample using SDT lysis buffer (4% SDS, 100 mM Tris-HCl, pH 7.6) and quantified via the BCA assay (P0012, Beyotime). 20 µg protein was resolved by SDS-PAGE (4%-20% gradient gel, 180 V, 45 min) following Coomassie Brilliant Blue R-250 staining for visualization. Proteins were then reduced with DTT (10 mM,RT, 1 h) and alkylated with IAA (50 mM, RT, 30 min, protected from light), following trypsin digestion. After desalination, the proteins were reconstituted in 40 µL of 0.1% formic acid (FA, 06450, Fluka). iRT standard peptides were spiked into each sample prior to analysis. Peptides were separated via nanoflow chromatography (Evosep One system) and analyzed by DIA (data-independent acquisition) using a timsTOF mass spectrometer (Bruker Daltonics, Bremen, Germany). DIA Data were processed using Spectronaut 19 with default settings. Protein identification and quantification were performed using MaxQuant software (version 1.6.14).

### RNA-sequencing

After RNA extraction using TRIzol™ Reagent (Invitrogen,15596026CN), RNA integrity and total amount were assessed using the Agilent 2100 Bioanalyzer. 1 μg mRNA enriched with poly(A) tails was obtained using Oligo(dT) magnetic beads (NEB, S1419), followed by random fragmentation of the mRNA in Fragmentation Buffer using divalent cations. Fragmented mRNA was used as a template to synthesize the first cDNA strand with random oligonucleotides as primers in the presence of M-MuLV reverse transcriptase. Subsequently, the RNA strand was degraded by RNaseH, and the second cDNA strand was synthesized using dNTPs as substrates in the DNA polymerase I system (NEB, M0209). The purified double-stranded cDNA was subjected to end repair, A-tailing, and sequencing adapter ligation. The cDNA fragments of approximately 370-420 bp were selected using AMPure XP beads (Beckman Colter), amplified by PCR, purified again with AMPure XP beads, and the final library was obtained. After library construction, the library was initially quantified using a Qubit 2.0 Fluorometer (Life Technologies), diluted to 1.5 ng/μL, and the insert size of the library was checked using the Agilent 2100 Bioanalyzer. Once the insert size met the expectations, qRT-PCR was performed to accurately quantify the effective concentration of the library (library effective concentration above 2 nM). Subsequently, in the sequencing flow cell, four fluorescently labeled dNTPs, DNA polymerase, and adapter primers were added for amplification. As each complementary strand was extended in each sequencing cluster, the release of corresponding fluorescence occurred with the addition of a fluorescently labeled dNTP. The sequencer (Illumina, NovaSeq 6000) captured the fluorescence signals and converted them into sequencing peaks using computer software to obtain the sequence information of the target fragments.

### Animal experiments

For the immunocompetent bone metastasis mouse model, Luci-Lewis lung cancer cells were injected into 4-6-week-old Female C57BL/6 mice (RRID: MGI:7264769) via the tibial metaphysis, with corresponding treatment groups established (e.g., PD-1 mAb, IL6 neutralizing antibody, ICA (MCE, HY-N0678), O-GlcNAcase inhibitor (OGA-i, MCE, HY-12588, etc.). On day 21 post-cell injection, mice were euthanized, bone metastasis tissues were collected, and subsequent analyses were performed using flow cytometry or Western blot. Female mice were selected to minimize the confounding effects of aggressive behavior often observed in group-housed male mice and to ensure consistency in bone remodeling rates, which can be sexually dimorphic. Findings in this model are representative of the specific sex used.

### Flow cytometry analysis

Tumor tissues were excised and minced into pieces. The tissue was digested with an enzyme working solution containing collagenase IV (Shanghai Maokang, MX1004), hyaluronidase (Shanghai Maokang, MX1007), and DNase I (Thermo Fisher, EN0521) at 37 °C for 1-2 h to dissociate the cells. The cell suspension was then passed through a 70 µm cell strainer to remove tissue debris and centrifuged at 800 × *g* for 5 min. The cell pellet was resuspended in PBS and subjected to density gradient centrifugation using 30% Percoll (Yeasen,40501ES60). The resulting cells were then incubated with the indicated antibodies according to the manufacturer’s instructions. Flow cytometry was performed to analyze the CD8^+^ T or Treg cell positive rate.

The positive rate of CD8^+^ T cells was analyzed by gating on CD45^+^CD8^+^IFNγ^+^ cells, and the positive rate of Treg cells was analyzed by gating on CD45^+^CD4^+^FoxP3^+^ cells. CD45 Monoclonal Antibody (APC, Invitrogen, 17-0451-82), CD8a Monoclonal Antibody (PerCP-Cyanine5.5, Invitrogen, 45-0081-82), IFNγ Monoclonal Antibody (FITC, Invitrogen, 11-7311-41), CD4 Monoclonal Antibody (PE, Invitrogen, 12-0041-82), CD25 Monoclonal Antibody (FITC, Invitrogen, 11-0251-82), FOXP3 Monoclonal Antibody (FITC, Invitrogen, 11-5773-82).

### H&E Staining and Immunohistochemistry

Tissue samples were fixed in 4% formalin (MACKLIN, P804537) for 24 hours, dehydrated through a graded ethanol series, and then embedded in paraffin. The tissues were sectioned into 4-6 µm thick slices and mounted on slides. After deparaffinization and rehydration, antigen retrieval was performed using citrate buffer (Biosharp, BL604A). The sections were then incubated with the primary antibody at 37 °C for 1 hour or overnight at 4 °C. After washing, the sections were incubated with the secondary antibody at 37 °C for 1 h. Color development was performed using 3,3-diaminobenzidine (DAB) (Bioss, AR1025), followed by hematoxylin counterstaining for the nuclei. Images of the stained pathological sections were captured under a microscope, and pathological analysis was conducted using ImageJ (RRID: SCR_003070).

### Western blot

Total proteins were extracted from the cells, and 30 µg of protein from each sample was loaded for separation by SDS-PAGE. Following electrophoretic separation, the proteins were transferred onto a PVDF membrane. The membrane was then blocked with 5% non-fat milk and incubated with primary antibodies at room temperature for 1.5 h. After washing, the membrane was incubated with secondary antibodies at 37 °C for 1 h, followed by ECL detection (Proteintech, PK10002). The band intensity was quantified using ImageJ (RRID: SCR_003070) and normalized to the corresponding Actin loading control. The antibodies used and their dilutions are as follows: YBX1 (Proteintech, 20339-1-AP, RRID: AB_10665424, 1:10000), O-GlcNAc (CST, 9875, RRID: AB_10950973), OGT (Proteintech, 66823-1-Ig, RRID: AB_2882166, 1:10000), HA tag (Abclonal, AE008, RRID: AB_2770404, 1:5000), MYC tag (Abcam, ab32, 1:800, RRID: AB_303599), β-actin (CST, 4967S, RRID: AB_330288), Histone H3 (CST, 14269, RRID: AB_2756816, 1:1000), GST-tag (CST, 2622, RRID: AB_331670, 1:1000). Integrin β3 (CST, 13166, AB_2798136), MMP9 (CST, 3852, AB_2144868), CTSK(Proteintech, 11239-1-AP, AB_2245581), DC-STAMP (Millipore, MABF39-I, AB_10807703), GAPDH (Proteintech, 60004-1-Ig, AB_2107436), Flag (Abclonal, AE005, AB_2770401), COX (Proteintech, 12211-1-AP, AB_2084233), LC3 (Proteintech, 14600-1-AP, AB_2137737), P62 (Proteintech, 18420-1-AP, AB_10694431), OGA (O-GlcNAcase, Proteintech, 14711-1-AP, AB_2143063).

### RNA Extraction and RT-qPCR

Cells were collected and lysed with Trizol Reagent (Invitrogen, 15596018CN). One-fifth volume of chloroform was added, and the mixture was vortexed for 30 s, followed by centrifugation at 12,000 x *g* for 15 min. The aqueous phase was transferred to a new tube, and an equal volume of isopropanol was added to precipitate the RNA. The RNA pellet was washed with 70% ethanol to obtain purified total RNA. The RevertAid First Strand cDNA Synthesis Kit (Thermo Fisher, K1622) was used for reverse transcription of RNA into cDNA according to the manufacturer’s instructions. The Taq Pro Universal SYBR qPCR Master Mix (Vazyme, Q712) was used for quantitative real-time PCR (qPCR) to detect gene expression levels, following the kit’s protocol. *YBX1*-mmu:Forwards:5’-AGG TCA TCG CAA CGA AGG TT-3’, Reverse:5’-TCC GCA CCC TTT TCT CCT TC-3’, *YBX1*-hsa: Forwards: 5’-AGG TCA TCG CAA CGA AGG TT-3’, Reverse: 5’-AAT GGT TAC GGT CTG CTG CA-3’,*CCL5*-hsa:Forwards: 5’-AGG ATC AAG ACA GCA CGT GG-3’, Reverse: 5’-GCA GAG GGC AGT AGC AAT GA-3’, *IL6*-hsa: Forwards: 5’-AGA GGC ACT GGC AGA AAA CA-3’, Reverse: 5’-GCT CTG GCT TGT TCC TCA CT-3’, RANKL-hsa: Forwards: 5’-CCT GTA CTT TCG AGC GCA GA-3’, Reverse: 5’-CCA CAT CCA ACC ATG AGC CT-3’, *IL6*-hsa-promoter: Forwards: 5’-ATG CCC AAC AGA GGT CAC TG-3’, Reverse: 5’-AAC CAG ACC CTT GCA CAA CA-3’,*CCL5*-hsa-promoter: Forwards: 5’-AGT GGA ATA GTG GCT GGC AC-3’, Reverse: 5’-CAG TTG ATC TGA GCT GGG CA-3’.

### RNAi and Transfection

The small interfering RNAs (siRNAs) used in this study were synthesized by Shanghai GenePharma Co., Ltd. The sequences of the siRNAs are listed below. Transfection was performed using Lipofectamine™ RNAiMAX Transfection Reagent (Invitrogen, 13778075) according to the manufacturer’s instruction. After 24-48 hours of incubation, subsequent experiments were performed. si-OGT#1 (Sense, 5’-GAAGAAAGUUCGUGGCAAAUU-3’; Antisense, 5’-UUU GCC ACG AAC UUU CUU CUU-3’); si-OGT#2 (Sense, 5’-GGGAAUGUGUACAAGGAAAUU-3’; Antisense, 5’-UUU CCU UGU ACA CAU UCC CUU-3’)；si-OGT#3 (Sense:5’- GCA CAA UCC UGA UAA AUU UUU-3’; Antisense:5’-AAA UUU AUC AGG AUU GUG CUU-3’); si-NC (Sense, 5’-UUC UCC GAA CGU GUC ACG UUU-3’; Antisense, 5’-ACG UGA CAC GUU CGG AGA AUU-3’).

### Plasmids and transfection

The full-length and T271A mutant YBX1 were cloned into pcDNA 3.1(+)-flag vector. The promoter regions comprising 1000 base pairs upstream of the transcriptional start site (TSS) of *IL6* and *CCL5* were cloned into pGL3-basic (RRID: Addgene_48743). Lipofectamine™ 2000 Transfection Reagent (Invitrogen, 11668027) was used for plasmid transfection according to the manufacturer’s instructions. Briefly, plasmids and lipofectamine 2000 were mixed into Opti-MEM and added into cell culture for 4-6 h. After 48 h of incubation, the cells were collected for subsequent analysis.

### Fluorescence recovery after photobleaching (FRAP) experiment

In vitro FRAP experiments was performed using laser confocal microscopy under 100 × oil immersion conditions. A 100% 488 nm laser pulse was used to bleach a specified area in the field, and the recovery post-bleaching was recorded over time.

### Fluorescence colocalization

To delineate the subcellular localization of YBX1 relative to mitochondria, lysosomes and autophagosomes, cells were triple-labeled with anti-YBX1 (Alexa Fluor 488, Proteintech, 20339-1-AP, 1:500), anti-TOM20 (mitochondria, Alexa Fluor 594, Abclonal, A19403, 1:100), and anti-LAMP1 (lysosomes, Alexa Fluor 555, Proteintech, 21997-1-AP, 1:500) and anti-LC3 (autophagosomes, Alexa Fluor 555, Proteintech, 14600-1-AP,1:600). The information of the secondary antibodies used is as follows: Multi-rAbTM CoraLite® Plus 488-Goat Anti-Rabbit Recombinant Secondary Antibody (H + L) (Proteintech, RGAR002,1:200), Multi-rAbTM CoraLite® Plus 555-Goat Anti-Rabbit Recombinant Secondary Antibody (H + L) (Proteintech, RGAR003, 1:200), Multi-rAbTM CoraLite® Plus 594-Goat Anti-Rabbit Recombinant Secondary Antibody (H + L) (Proteintech, RGAR004, 1:200). Confocal Z-stack imaging (NIKON, C2^+^) was performed to assess YBX1-mitochondria co-localization and mitochondrial-lysosomal interactions under experimental treatments. Co-localization coefficients were calculated using Image J software (RRID: SCR_003070). To enhance visualization, the images acquired using Alexa Fluor 594 fluorescence were pseudocolored green.

### Multiplex immunohistochemistry (mIHC)

The paraffin-embedded sections with a thickness of 5 µm were mounted on glass slides, deparaffinized using a dewaxing solution (Servicebio, G1128), and dehydrated through a graded ethanol series. Subsequently, the sections were treated with antigen retrieval buffer (Servicebio, G1202) and 3% hydrogen peroxide at room temperature for 25 min protected from light. A blocking solution of 3% bovine serum albumin (Servicebio, GC305010) was applied and incubated at room temperature for 30 minutes. The sections were then incubated overnight at 4 °C with the following primary antibodies, respectively, followed by three washes with PBS buffer (pH 7.4): FOXP3 (iF546-Tyramide, Proteintech, 65089-1-Ig, 1:500), CD4 (iF594-Tyramide, Proteintech, 86300-3-RR, 1:500), IFN-γ (iF647-Tyramide, Proteintech, 29788-1-AP, 1:500), YBX1 (iF700-Tyramide, Proteintech, 20339-1-AP, 1:400), CD8 (iF488-Tyramide, Proteintech, 85977-4-RR, 1:500), and CD25 (iF440-Tyramide, CST, 39475S, 1:200). Thereafter, the corresponding species-specific secondary antibody working solutions were applied: HRP-labeled goat anti-mouse IgG (Servicebio, GB23301, 1:500) and HRP-labeled goat anti-rabbit IgG (Servicebio, GB23303, 1:500), and incubated at room temperature for 50 min. After three washes with PBS, signal amplification was performed using iF440-Tyramide (Servicebio, G1250, 1:500), iF488-Tyramide (Servicebio, G1231, 1:500), iF546-Tyramide (Servicebio, G1251, 1:500), iF594-Tyramide (Servicebio, G1242, 1:500), iF647-Tyramide (Servicebio, G1232, 1:500), and iF700-Tyramide (Servicebio, G1258, 1:500), respectively. After immunostaining, cell nuclei were counterstained with DAPI (Servicebio, G1012), and the slides were coverslipped for scanning.

### ELISA

Human serum concentrations of IL6 and CCL5 were quantified using ELISA kits (R&D Systems #D6050 and #DRN00B). Mice serum concentrations of IL6 and CCL5 were quantified using ELISA kits (R&D Systems # M6000B and MMR00). Absorbance values were normalized to standard curves, and data were expressed as pg/mL.

### Natural compound screening

To identify natural compounds capable of modulating YBX1 activity, we constructed the 3xMotif of YBX1 into pGL3-promoter to generate a YBX1-driven luciferase reporter (pGL3-YBX1-Motif). For high-throughput screening, HEK293T cells were transfected pGL3-promoter-YBX1. 12 h after transfection, cells were treated with 10 μM library for 24 h, and the luciferase activity was measured using Luciferase Assay System with Reporter Lysis Buffer (Promega, E4030). This system was employed to screen a small-molecule compound library in HEK293T cells. Compounds that significantly altered luciferase activity were selected as potential YBX1-targeting candidates.

### CRISPR-Cas9 technique to knock out YBX1

The CRISPR-Cas9 technique was used to knock out YBX1 in A549^High-M^ and H1975^High-M^ or LLC cells. The homologous gene sequences of YBX1 in humans and mice were selected, and sgRNA sequences were designed using the CHOPCHOP website (https://www.bioinformatics.org/crispr-design-tool). The sgRNA DNA Oligo sequences were synthesized by Shanghai Sangon Biotech. Specific sgRNAs were generated using the BeyoCRISPR™ One-Step sgRNA Synthesis Kit (Beyotime, D7081S). The sgRNAs were incubated with TrueCut™ Cas9 Protein v2 at room temperature for 20 min. Subsequently, Cas9 ribonucleoprotein (RNP) complexes were delivered into cells via electroporation. Three days later, the knockout efficiency of YBX1 was assessed by qPCR. sgYbx1#1, GCG ATA GGG CCG GCG TTG TTGGG；sgYbx1#2, TGC GAT AGG GCC GGC GTT GTTGG；sgYbx1#3, AAC AAC GCC GGC CCT ATC GCAGG.

### Luciferase reporter gene assay for promoter activity analysis

Cells were seeded in 24-well plates at a density of 1 × 10^5^ cells per well before plasmid transfection. pGL3-IL6-promoter (or pGL3-CCL5-promoter), OE/YBX1/NC, and pRL-TK were co-transfected into cells. After 48 h of transfection, luciferase activity was measured using the Dual-Luciferase® Reporter Assay System (Promega, E1910) according to the manufacturer’s instructions. The luminescence was detected using a multifunctional microplate reader (MD, SpectraMax Mini).

### Chromatin immunoprecipitation (ChIP) seq

The chromatin immunoprecipitation (ChIP) assay was performed using a SimpleChIP® Plus Sonication Chromatin IP Kit (CST, 56383). Briefly, cells were cross-linked with 1% formaldehyde for 20 min, and the reaction was quenched by adding glycine for 5 min. After collection, the samples were digested with micrococcal nuclease for 20 min, and the enzymatic reaction was halted with EDTA. The DNA was subsequently fragmented by sonication. Immunoprecipitation was carried out using an anti-YBX1 antibody. The immunoprecipitated DNA was treated with RNase A and proteinase K, followed by purification via phenol-chloroform extraction and ethanol precipitation. Libraries for sequencing were constructed using the NEBNext® UltraTM II DNA Library Prep Kit for Illumina (NEB, E7645L), and high-throughput sequencing was performed on an Illumina NovaSeq platform with 150 bp paired-end reads.

### ChIP-qPCR

ChIP-qPCR was performed using the SimpleChIP® Plus Sonication Chromatin IP Kit (CST, 56383). Initially, DNA and proteins were cross-linked and sonicated into small fragments. DNA fragments were then captured using an anti-YBX1 antibody or IgG as a control. The captured DNA was subsequently purified and subjected to qPCR analyses. The enrichment of YBX1 binding at the promoter regions of the IL-6 and CCL5 genes was detected by qPCR.

### Transwell assay

To assess cell invasion and migration capabilities, Transwell chambers (Corning, 3422) with and without Matrigel coating were used. Cells (5 × 10^5^) were seeded into the chambers, which were then placed in the wells of 24-well plate. After 24 h of invasion or migration, the chambers were collected and fixed with methanol. The cells were stained with crystal violet staining solution (Beyotime, C0121). After staining, the chambers were washed with water to remove non-invading/migrating cells from the interior, air-dried, and images were captured under a microscope. The number of migrating/invading cells was quantified using Image J (RRID: SCR_003070).

### Immunoprecipitation

500 μg of cell lysates in IP Lysis Buffer (20 mM Tris-HCl pH 7.5, 150 mM NaCl, 1 mM EDTA, 0.1% NP-40, 10% glycerol, 1 mM DTT, 1 × protease inhibitors were incubated with anti-YBX1 for 4–6 h at 4 °C with rotation, followed by addition with Protein A/G Magnetic Beads (MCE, HY-K0202) and incubated for further 8–12 h. After washing with IP Lysis Buffer, incubate the beads in 1 × SDS-PAGE Loading Buffer at 1200 rpm and 95 °C for 10 min. The products are then analyzed by Western Blot.

### GST Pull-Down assay

The GST pull-down assay was performed using the GST Protein Interaction Pull-Down Kit (Pierce, 21516). Purified GST-YBX1 was prepared using BL21 (DE3) E. coli strain (Sangon, B528414) and Glutathione Magnetic Beads (Thermo Fisher, 78601). The purified GST-YBX1/GST-OGT was then incubated with cell lysates overexpressing His-OGT/His-YBX1 according to the kit’s instructions. After incubation, washing, and elution steps, the target proteins were separated by SDS-PAGE gel electrophoresis and visualized using Coomassie Brilliant Blue staining.

### Statistical analysis

Statistical analyses were performed using GraphPad Prism 8.0. All quantitative data obtained from at least three independent experiments are presented as the mean ± standard deviation (SD). For comparisons between two groups, a two-tailed Student’s *t* test was applied. For comparisons among more than two groups, one-way analysis or two-way of variance (ANOVA) was employed, followed by Tukey’s or Dunnett’s post hoc test for multiple comparisons. Statistical significance was determined by setting a *p*-value criterion of less than 0.05 for all analyses. *P*- values are showed in the figure.

### Reporting summary

Further information on research design is available in the Nature Portfolio Reporting Summary linked to this article.

## Supplementary information

Supplementary Information

Reporting Summary

Transparent Peer Review file

## Source data

Source Data

## Data Availability

Source data are provided with this paper. The raw files of proteome datasets and PRM data can be obtained from the iProX database (https://www.iprox.cn/page/PSV023.html?url=1778118828235rdzg, accession code: fmkq), and the Genomics Data can be obtained from the GEO database (https://www.ncbi.nlm.nih.gov/sra/?term=PRJNA1460874, https://www.ncbi.nlm.nih.gov/sra/SRX33185140[accn]). Source data are provided in this paper.
