# Supplementary material for: YBX1 Confers immunosuppressive bone metastatic traits in non-small cell lung cancer
Source: Nat Commun. 2026 Jun 13;17:7505. doi: 10.1038/s41467-026-73931-2 (PMC13407896; doi:10.1038/s41467-026-73931-2)
Supplement: Supplementary file 1 — Supplementary Information [file 41467_2026_73931_MOESM1_ESM.pdf]

Figure S1

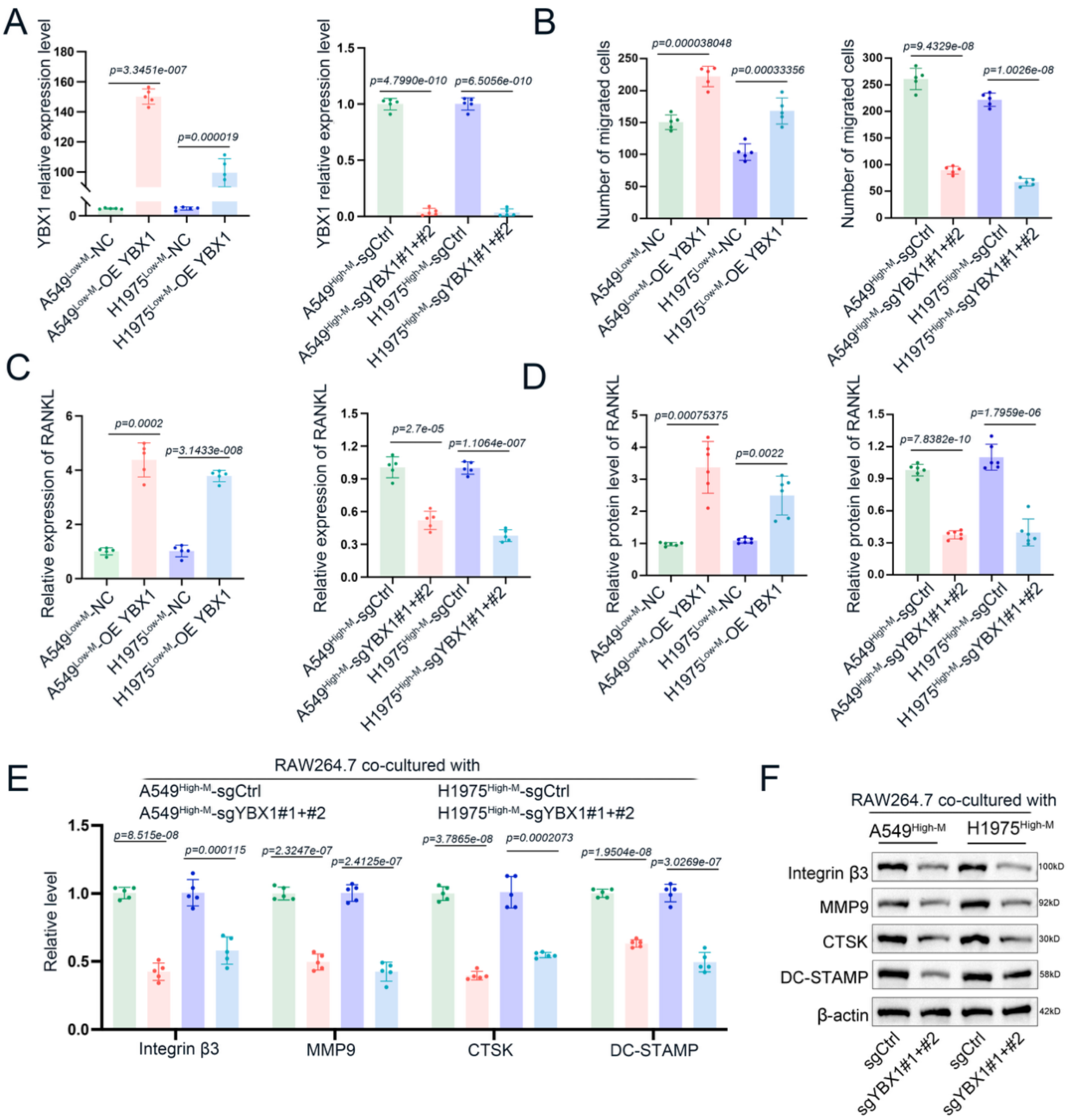

Figure S1. YBX1 Enhances the Invasiveness of Cancer Cells and Activates the RANKL Signaling Pathway to Promote Osteoclast Differentiation

(A) RT-qPCR validation of the efficiency of YBX1 overexpression in low bone-metastatic potential cells (A549^{Low-M}, H1975^{Low-M}) and YBX1 knockout in high bone-metastatic potential cells (A549^{High-M}, H1975^{High-M}). (*n=5, independent experiments*). *Unpaired t test with Welch's correction, Mean*±SD.

(B) Transwell migration assay analyzing the effect of altered YBX1 expression on the migratory ability of lung adenocarcinoma cells. (*n=5, independent experiments*). *Unpaired t test, Mean*±SD.

(C-D) Effect of altered YBX1 expression levels on RANKL mRNA (C) and protein (D) expression in cancer cells, detected by RT-qPCR (C, *n=5, independent experiments*,

Unpaired t test, Mean±SD.) and ELISA (D, *n=6, independent experiments, Unpaired t test with Welch's correction, Mean*±SD), respectively.

(E-F) Changes in the expression of key osteoclast markers (integrin β3, MMP9, CTSK, DC-STAMP) detected by RT-qPCR (E) and Western blot (F) in osteoclast precursor RAW264.7 cells after co-culture with high bone-metastatic potential cells. (*n=5, independent experiments, Unpaired t test, Mean*±SD).

Figure S2

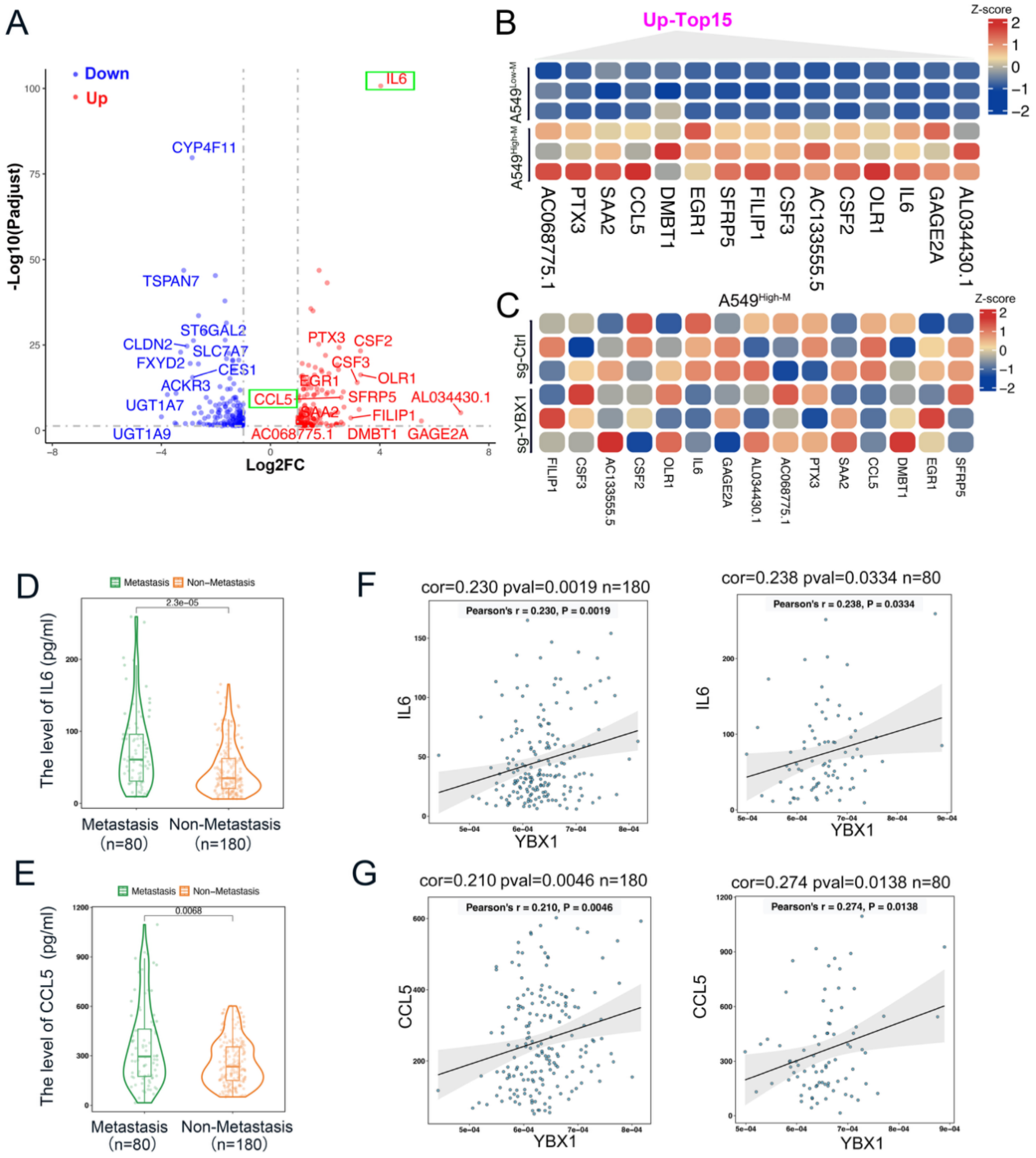

Figure S2. Identification of *IL6* and *CCL5* as Downstream Effectors of YBX1 via RNA-seq Screening

(A) Volcano plot of differentially expressed genes from RNA-seq of A549$^{High\text{-}M}$ vs. A549$^{Low\text{-}M}$ cells, indicating *IL6* and *CCL5* as significantly upregulated genes ($Log_2FC > 1$, $Padjust < 0.05$).

(B) Clustered heatmap of differentially expressed genes, showing the top 15 upregulated genes in A549$^{High\text{-}M}$ versus A549$^{Low\text{-}M}$ cells (*n=3, independent experiments*).

(C) Heatmap representation of qRT-PCR validation of *IL6* and *CCL5* mRNA expression levels after knockout YBX1 in A549^{High-M} cells.

(D–E) Box plots comparing the expression levels of IL6 (D) and CCL5 (E) in serum from patients without metastasis at initial diagnosis (*n = 180*, *independent experiments*) versus tissues from patients with bone metastasis at initial diagnosis (*n=80, patient samples)*, *(*IL6*, p=2.3e-05;* CCL5*, p=0.0068).*

(F–G) Scatter plots showing positive correlations between YBX1 expression and IL6 (F) / CCL5 (G) expression levels in primary tumor tissues from patients with (*n=180*, *patient sanples*) / with (*n=80, patient samples*) bone metastasis at initial diagnosis (*cor = 0.230, p = 0.0019; cor = 0.238, p = 0.0334; cor = 0.210, p = 0.0046; cor = 0.274, p = 0.0138, respectively*).

Figure S3

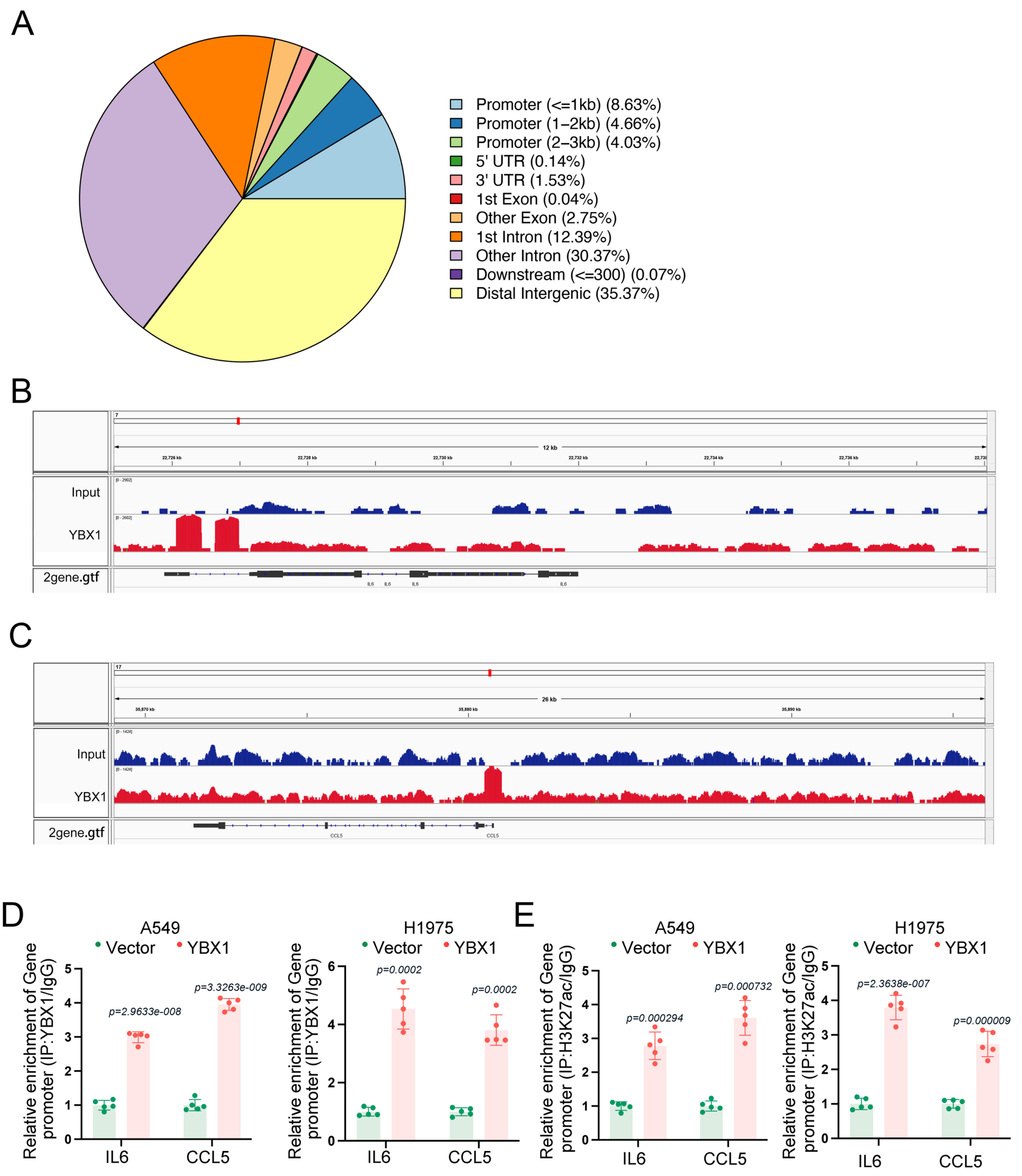

Figure S3. YBX1 Directly Binds to the *IL6* and *CCL5* Promoters and Regulates their Transcriptional Activity

(A-C) ChIP-seq results showing the global transcriptional regulation profile of YBX1 (A) and specifically extracted the peak profiles for *IL6* and *CCL5* (B-C).

(D) ChIP-qPCR validation of specific YBX1 enrichment at the *IL6* and *CCL5* promoter regions (*n=5, independent experiments*). *Unpaired t test, Mean*±SD.

(E) ChIP-qPCR detection showing enrichment of the active transcription mark H3K27ac at the *IL6* and *CCL5* promoter regions (*n=5, independent experiments*). *Unpaired t test with Welch's correction, Mean±SD.*

Figure S4

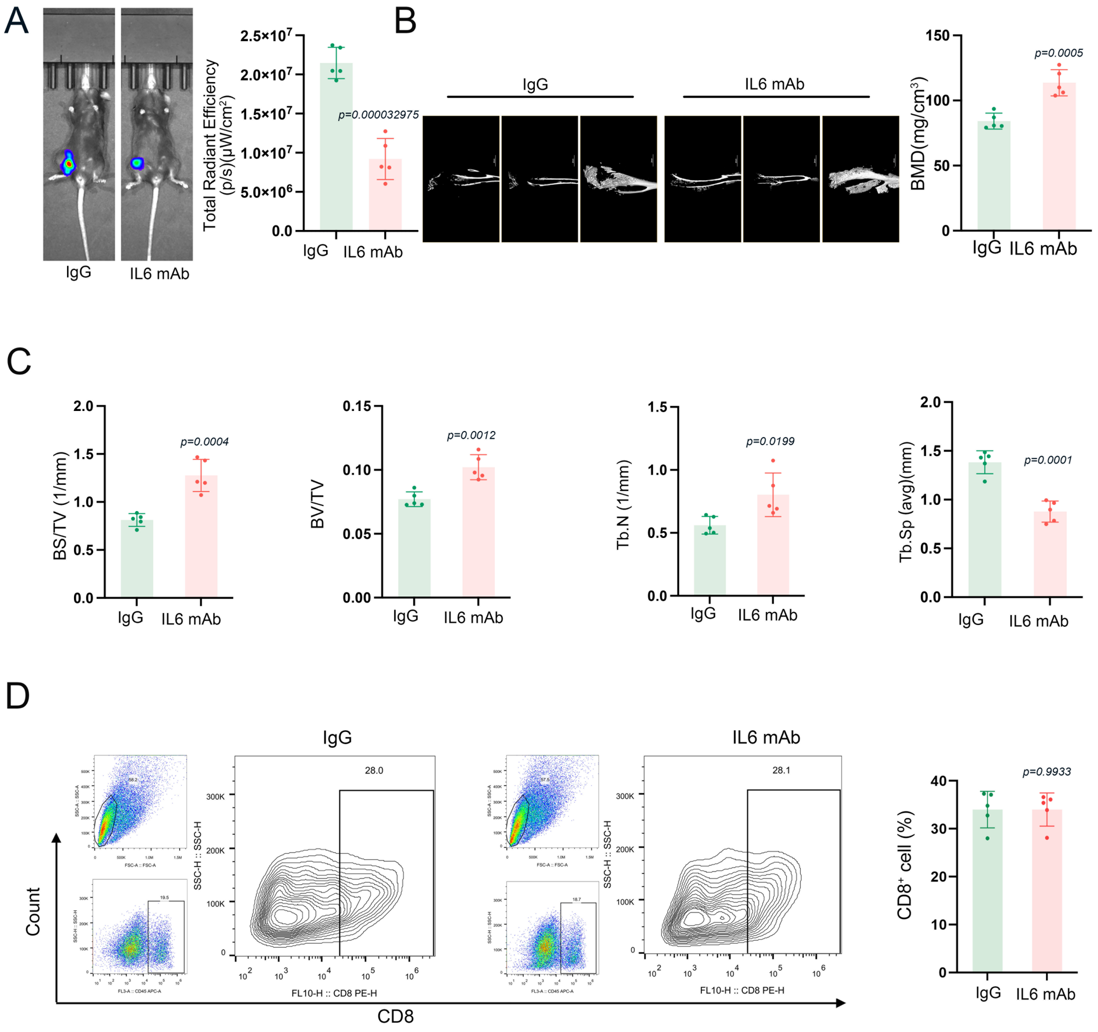

Figure S4. The Role of IL6 in YBX1-Driven Bone Metastasis

(A) Effect of IL6 neutralizing antibody treatment on tumor burden. (Left) Representative *in vivo* optical imaging of tumor-bearing mice treated with control IgG or IL6 neutralizing antibody (IL6mAb). (Right) Corresponding quantitative histogram of fluorescence imaging (*n=5, mice*). *Unpaired t test, Mean*±SD.

(B) (Left) Micro-CT scan images, (Right) Corresponding quantitative histogram of bone mineral density (BMD) (*n=5, mice*), indicating alleviated bone destruction. *Unpaired t test, Mean*±SD.

(C) Histograms show the improvement effects of the IL6 neutralizing antibody on trabecular bone surface area/tissue volume (BS/TV), bone volume/tissue volume (BV/TV), trabecular number (Tb.N), and trabecular separation (Tb.Sp) (*n=5, mice*). *Unpaired t test, Mean*±SD.

(D) Effect of IL6 neutralizing antibody treatment on $CD8^+$ T cell levels. (Left) Representative gating plots of $CD8^+$ T cells in flow cytometry analysis. (Right) Quantitative histogram of the percentage of $CD8^+$ T cells (*n=5, mice*). *Unpaired t test, Mean*±SD.

Figure S5

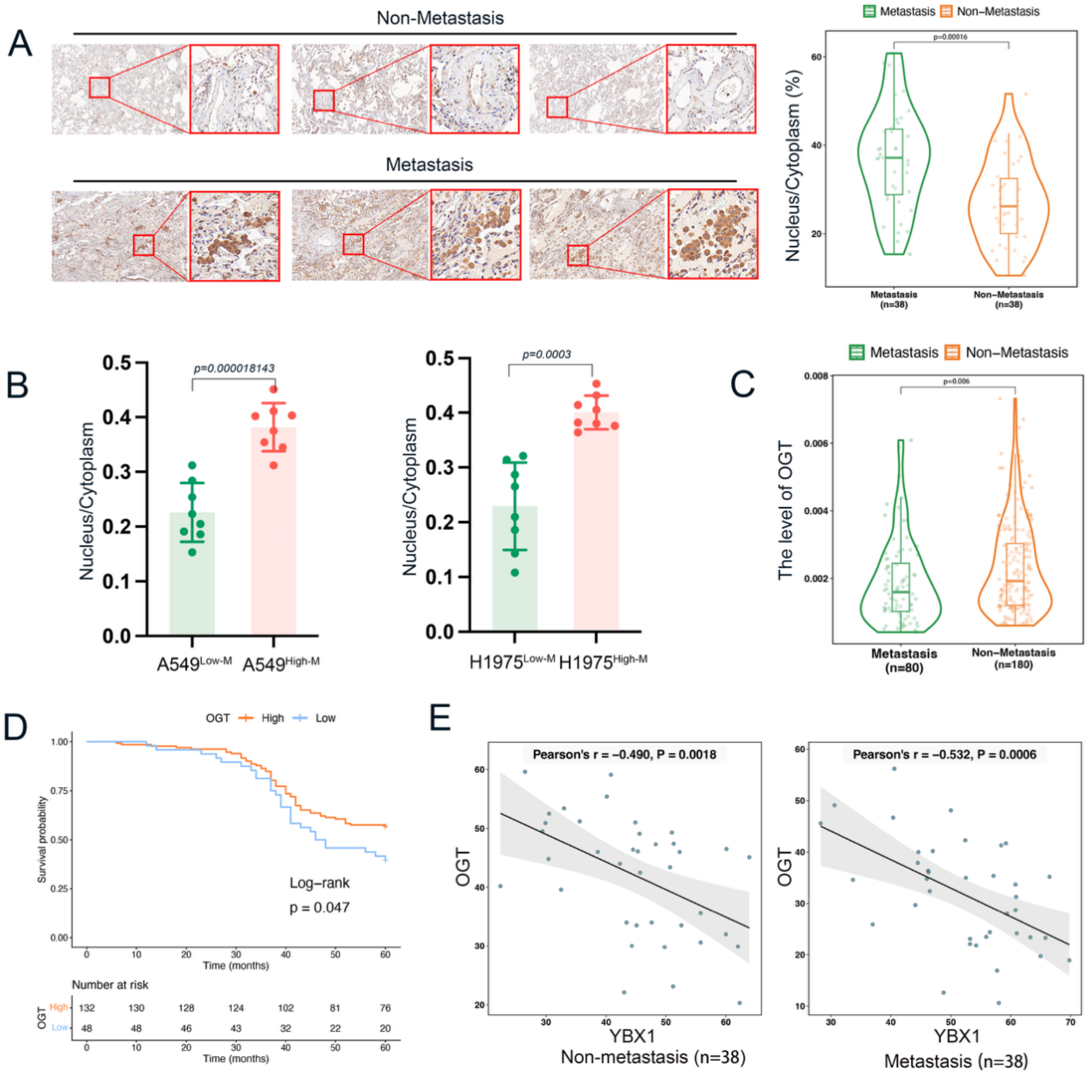

Figure S5. Analysis of YBX1 Expression and Nucleocytoplasmic Ratio in Metastatic Tissues, and Analysis of OGT Tissue Expression

(A) Immunohistochemical staining shows the nuclear/cytoplasmic localization of YBX1 in non-metastatic (*n=38, patient samples*) and metastatic (*n=38, patient samples*) tissues.

(B) Quantitative comparison of the nucleocytoplasmic ratio of YBX1 in high-metastasis and low-metastasis cells (*n=8, independent experiments*). *Left: Unpaired t test, Mean±SD; right: Unpaired t test with Welch's correction, Mean±SD.*

(C) OGT expression in primary tumor tissues from patients initially diagnosed without (n=180) or with (*n=80, patient samples*) bone metastasis (*p=0.006*).

(D) Analysis of the relationship between OGT expression and patient survival (*n=180, patient samples, p=0.047*).

(E) Correlation analysis between OGT and YBX1 expression in primary tumor tissues from patients initially diagnosed with (*n=38, patient samples*) or without (*n=38, patient samples*) bone metastasis (*r=-0.490, p=0.0018; r=-0.532, p=0.0006*).

Figure S6

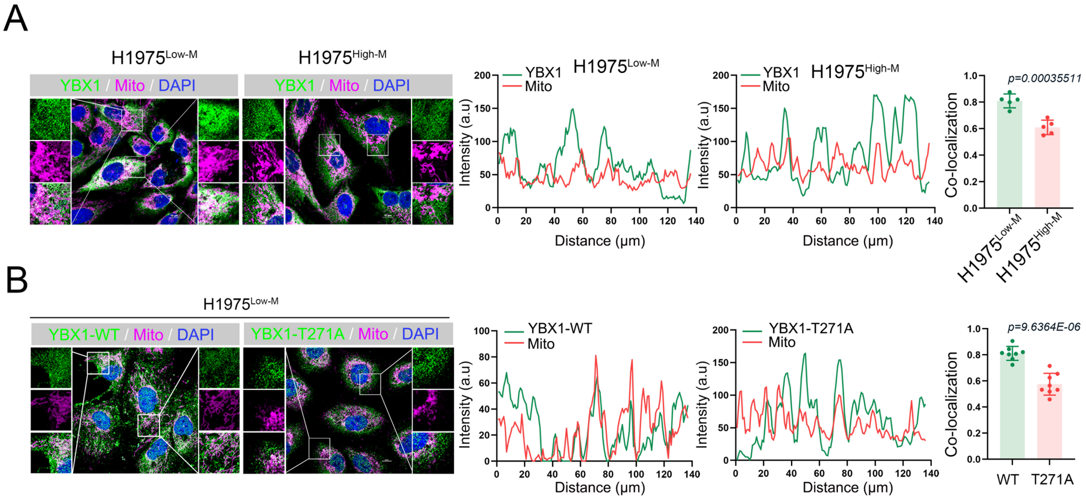

Figure S6. Validation that the mitochondrial localization of YBX1 in H1975 cells depends on the T271 site

(A-B) Immunofluorescence co-localization experiments (DAPI for nuclear staining, YBX1 antibody-green signal, mitochondrial marker-red signal) were used to analyze the co-localization level of YBX1 with mitochondria in low- and high-metastasis cells (A) (*n=5, ***p<0.001*), and the co-localization level of YBX1 with mitochondria after mutation of the T271 site (B) (*n=8, independent experiments*). *Unpaired t test, Mean±SD.*

Figure S7

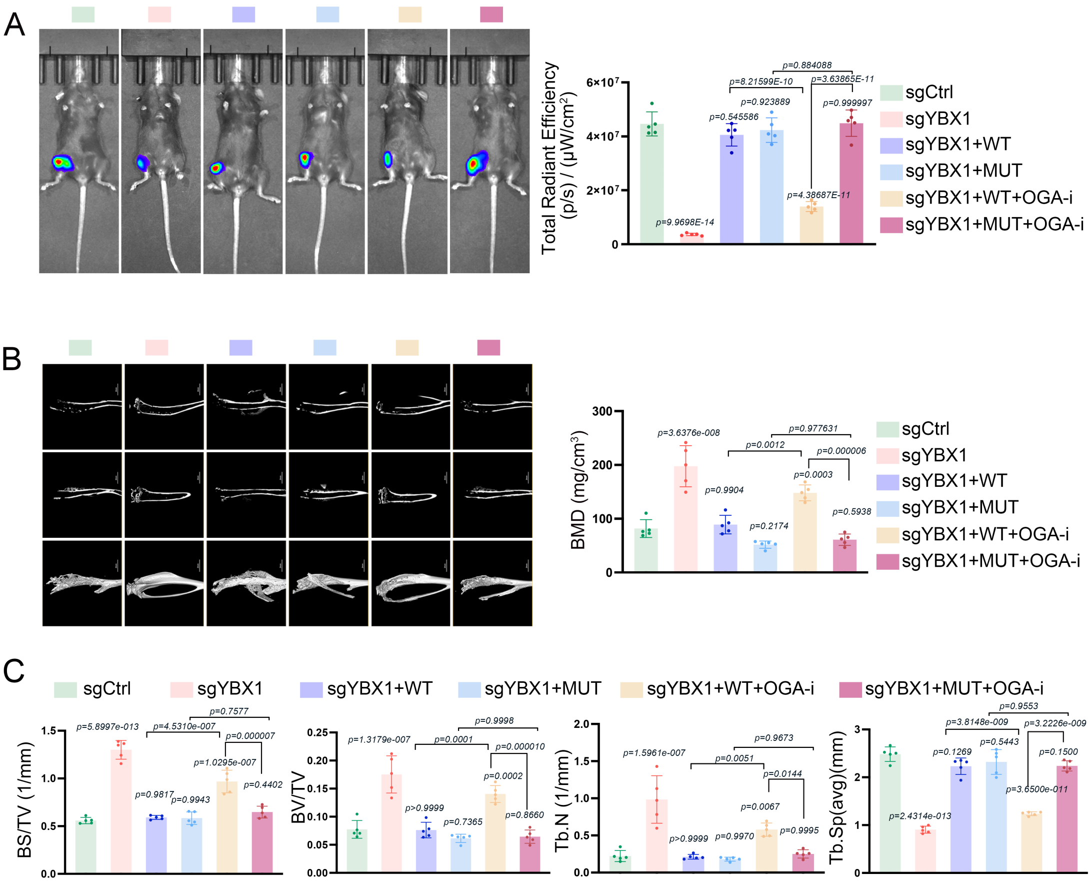

Figure S7. *In vivo* validation of the effect of YBX1 T271 site glycosylation modification on bone metastasis

(A) A bone metastasis animal model was established to analyze the effects of T271 site mutation and treatment with the OGA inhibitor Thiamet G (OGA-i) on tumor bone metastasis. Representative in vivo imaging pictures of mice from each experimental group (sgCtrl, sgYBX1, sgYBX1+WT, sgYBX1+MUT, sgYBX1+WT+OGA-i, sgYBX1+MUT+OGA-i) and the fluorescence signals indicating bone metastasis are shown. (*n=5, mice*). *Tukey's multiple comparisons test, Mean±SD.*

(B) Quantitative analysis of bone mineral density (BMD) corresponding to panel (A). (*n=5, mice*). *Tukey's multiple comparisons test, Mean±SD.*

(C) Quantitative analysis of bone histomorphometric parameters, including bone surface/tissue volume (BS/TV), bone volume/tissue volume (BV/TV), trabecular number (Tb.N), and trabecular separation (Tb.Sp). (*n=5, mice*). *Tukey's multiple comparisons test, Mean±SD.*

Figure S8

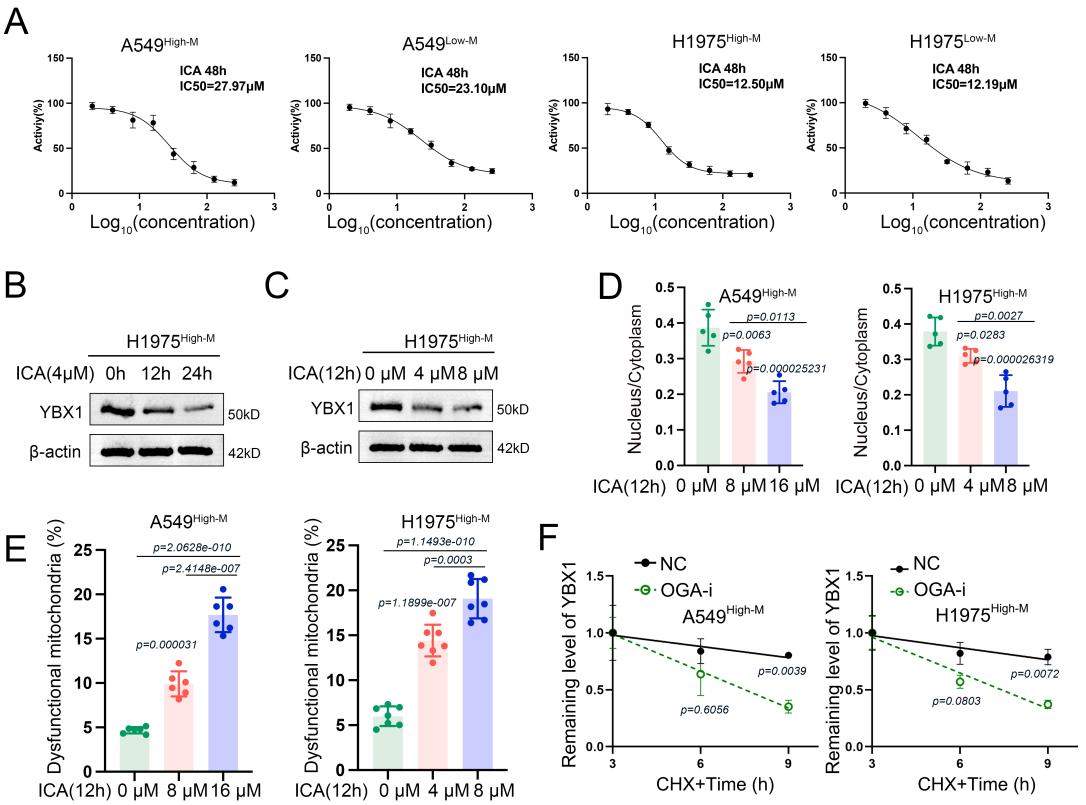

Figure S8. Detailed mechanistic study of ICA-induced YBX1 protein degradation

(A) IC50 values of ICA for four cell lines ($A549^{High-M}$, $A549^{Low-M}$, $H1975^{High-M}$, $H1975^{Low-M}$).

(B-C) Western blot analysis of YBX1 protein level changes in $H1975^{High-M}$ cells treated with ICA at different time points (0h, 12h, 24h) or concentrations (0 µM, 4 µM, 8 µM). *n=3, independent experiments*.

(D) Analysis of the effect of ICA treatment on the nucleocytoplasmic distribution of YBX1 protein. (*n=5, independent experiments*). *Tukey's multiple comparisons test, Mean±SD.*

(E) The effect of ICA on mitochondrial function. ($A549^{High-M}$ *n=6,* $H1975^{High-M}$ *n=7, independent experiments*). *Tukey's multiple comparisons test, Mean±SD.*

(F) Analysis of the effect of OGA inhibitor treatment on YBX1 degradation in $A549^{High-M}$ and $H1975^{High-M}$ cells after inhibiting protein synthesis with CHX. (*n=3, independent experiments*). *Bonferroni's multiple comparisons test, Mean±SD.*
