# Supplementary material for: YBX1 Confers immunosuppressive bone metastatic traits in non-small cell lung cancer
Source: Nat Commun. 2026 Jun 13;17:7505. doi: 10.1038/s41467-026-73931-2 (PMC13407896; doi:10.1038/s41467-026-73931-2)
Supplement: Supplementary file 2 — Reporting Summary [file 41467_2026_73931_MOESM2_ESM.pdf]

nature portfolio

Corresponding author(s): Prof. Lilin GE

Last updated by author(s): May 15, 2026

Reporting Summary

Nature Portfolio wishes to improve the reproducibility of the work that we publish. This form provides structure for consistency and transparency in reporting. For further information on Nature Portfolio policies, see our Editorial Policies and the Editorial Policy Checklist.

Statistics

For all statistical analyses, confirm that the following items are present in the figure legend, table legend, main text, or Methods section.

n/a	Confirmed	
☐	☒	The exact sample size (*n*) for each experimental group/condition, given as a discrete number and unit of measurement
☐	☒	A statement on whether measurements were taken from distinct samples or whether the same sample was measured repeatedly
☒	☐	The statistical test(s) used AND whether they are one- or two-sided<br>*Only common tests should be described solely by name; describe more complex techniques in the Methods section.*
☒	☐	A description of all covariates tested
☒	☐	A description of any assumptions or corrections, such as tests of normality and adjustment for multiple comparisons
☒	☐	A full description of the statistical parameters including central tendency (e.g. means) or other basic estimates (e.g. regression coefficient) AND variation (e.g. standard deviation) or associated estimates of uncertainty (e.g. confidence intervals)
☒	☐	For null hypothesis testing, the test statistic (e.g. *F*, *t*, *r*) with confidence intervals, effect sizes, degrees of freedom and *P* value noted<br>*Give P values as exact values whenever suitable.*
☒	☐	For Bayesian analysis, information on the choice of priors and Markov chain Monte Carlo settings
☒	☐	For hierarchical and complex designs, identification of the appropriate level for tests and full reporting of outcomes
☒	☐	Estimates of effect sizes (e.g. Cohen's *d*, Pearson's *r*), indicating how they were calculated

Our web collection on statistics for biologists contains articles on many of the points above.

Software and code

Policy information about availability of computer code

Data collection	Thermo Xcalibur 4.0.27.19 was used for collecting Mass spectrometry (MS) raw data. Spectronaut version X was used for quantifying raw data from DDA- and DIA-MS. Skyline version 3.6.0.10571 was used for quantifying quantitative data from PRM-MS.
Data analysis	Wkomics (https://omicsolution.org/wkomics/main/) analysis platform was used for the pre-processing of the proteomic data, statistical analysis, GO and KEGG analysis. Graphpad PRISM version 8 was used for drawing scatter plot, box plot, violin plot and column of data visualization. Online analysis tool String (https://cn.string-db.org/) was used for protein-protein interaction (PPI) analysis and Cytoscape 3.9.1 was used for the visualization of PPI network. Data analysis was performed in R version 4.0.2. using custom or publicly-available R package. Individual packages are explicitly cited in the manuscript and listed below. The code is available upon request and from GitHub (https://github.com/Mengchao-Hepatobiliary-Hospital/Serum-diagnostic-model).<br><br>Individual packages: mlbench v2.1-3 caret v6.0-90<br>randomForest v4.6-14 pROC v1.18.0<br>Mfuzz v2.48.0 reshape2 v1.4.4 corrplot v0.92

For manuscripts utilizing custom algorithms or software that are central to the research but not yet described in published literature, software must be made available to editors and reviewers. We strongly encourage code deposition in a community repository (e.g. GitHub). See the Nature Portfolio guidelines for submitting code & software for further information.

Data

Policy information about availability of data

All manuscripts must include a data availability statement. This statement should provide the following information, where applicable:

- Accession codes, unique identifiers, or web links for publicly available datasets
- A description of any restrictions on data availability
- For clinical datasets or third party data, please ensure that the statement adheres to our policy

The raw files of proteome datasets and PRM data can be obtained from iProX database (https://www.iprox.cn/page/PSV023.html;?url=1778118828235rdzg, accession code: fmkq), and the Genomics Data can be obtained from GEO database (https://www.ncbi.nlm.nih.gov/sra/?term=PRJNA1460874, https://www.ncbi.nlm.nih.gov/sra/SRX33185140[accn])

Research involving human participants, their data, or biological material

Policy information about studies with human participants or human data. See also policy information about sex, gender (identity/presentation), and sexual orientation and race, ethnicity and racism.

Reporting on sex and gender	Gender based analysis is performed and 76 (42.2 %) male and 104 (57.8 %) female patients were included. And in the bone metastasis cohort, 36 (45 %) male and 44 (55 %) female patients were included.
Reporting on race, ethnicity, or other socially relevant groupings	The participant patients were not grouped based on any social factors such as race or ethnicity.
Population characteristics	A total of 180 treatment-free non-small cell lung cancer (NSCLC) patients without metastasis at initial diagnosis were enrolled between August 2018 and December 2020. The follow-up duration spanned 60 months, from August 2018 to August 2024. Collected specimens included primary tumor tissues, whole blood samples, and histopathological slides. For comparison, a non-follow-up cohort was established, consisting of 80 lung adenocarcinoma patients with bone metastasis at initial diagnosis.
Recruitment	These patients were recruited from January 2018 to December 2024, with the same specimen types collected (primary tumor tissues, whole blood samples, and histopathological slides).
Ethics oversight	This project was approved by the Institution Review Board of Hospital. Informed consent was obtained from each participant before the operation. The use of clinical specimens was completely in compliance with the “Declaration of Helsinki”.

Note that full information on the approval of the study protocol must also be provided in the manuscript.

Field-specific reporting

Please select the one below that is the best fit for your research. If you are not sure, read the appropriate sections before making your selection.

☒ Life sciences ☐ Behavioural & social sciences ☐ Ecological, evolutionary & environmental sciences

For a reference copy of the document with all sections, see nature.com/documents/nr-reporting-summary-flat.pdf

Life sciences study design

All studies must disclose on these points even when the disclosure is negative.

Sample size	No prior sample size calculation was performed. The collection of samples depends on the inclusion criteria and complete clinical information in in the clinical specimen banks.
Data exclusions	No data were excluded from the analyses.
Replication	No replicated measurement of samples was performed in the study. MS data collection all used independent samples.
Randomization	In mouse studies, mice were randomly allocated to treatment groups before initiation of the experimental intervention. Within the patient cohort, cancer patients fulfilling the enrollment criteria were recruited and subsequently stratified according to the stage of lung cancer bone metastasis.
Blinding	Investigators were not blind during the analysis.

Reporting for specific materials, systems and methods

We require information from authors about some types of materials, experimental systems and methods used in many studies. Here, indicate whether each material, system or method listed is relevant to your study. If you are not sure if a list item applies to your research, read the appropriate section before selecting a response.

Materials & experimental systems

n/a	Involved in the study	
☐	☒	Antibodies
☐	☒	Eukaryotic cell lines
☒	☐	Palaeontology and archaeology
☐	☒	Animals and other organisms
☒	☐	Clinical data
☒	☐	Dual use research of concern
☒	☐	Plants

Methods

n/a	Involved in the study	
☒	☐	ChIP-seq
☐	☒	Flow cytometry
☒	☐	MRI-based neuroimaging

Antibodies

Antibodies used

YBX1 Proteintech 20339-1-AP 1:10000 AB_10665424
O-GlcNAc CST 9875 1:1000 AB_10950973
OGT Proteintech 66823-1-Ig 1:10000 AB_2882166
HA tag Abclonal AE008 1:5000 AB_2770404
MYC tag Abcam ab32 1:800 AB_303599
β-actin CST 4967S 1:1000 AB_330288
Histone H3 CST 14269 1:1000 AB_2756816
GST-tag CST 2622 1:1000 AB_331670
Integrin β3 CST 13166 1:1000 AB_2798136
MMP9 CST 3852 1:1000 AB_2144868
CTSK Proteintech 11239-1-AP 1:800 AB_2245581
DC-STAMP Millipore MABF39-I 1:250 AB_10807703
YBX1 Proteintech 20339-1-AP 1:10000 AB_10665424
O-GlcNAc CST 9875 1:1000 AB_10950973
OGT Proteintech 66823-1-Ig 1:10000 AB_2882166
HA tag Abclonal AE008 1:5000 AB_2770404
MYC tag Abcam ab32 1:800 AB_303599
β-actin CST 4967S 1:1000 AB_330288
Histone H3 CST 14269 1:1000 AB_2756816
GST-tag CST 2622 1:1000 AB_331670
Integrin β3 CST 13166 1:1000 AB_2798136
MMP9 CST 3852 1:1000 AB_2144868
CTSK Proteintech 11239-1-AP 1:800 AB_2245581
DC-STAMP Millipore MABF39-I 1:250 AB_10807703

Validation

Proteintech 20339-1-AP
CST 9875
Proteintech 66823-1-Ig
Abclonal AE008
Abcam ab32
CST 4967S
CST 14269
CST 2622
CST 13166
CST 3852
Proteintech 11239-1-AP
Millipore MABF39-I

Eukaryotic cell lines

Policy information about cell lines and Sex and Gender in Research

Cell line source(s)

DMEM medium Gibco C11995500BT
RPMI 1640 medium Gibco C11875500BT
F-12K medium Gibco 21127022
penicillin-streptomycin Gibco 15140122
Glutamax Gibco 35050061
sodium pyruvate Pricella PB180422
fetal bovine serum Gibco A5670701
Luci-H1975 FuHeng FH0183
Luci-A549 the Cell Bank of the Chinese Academy of Sciences
Luci-Lewis the Cell Bank of the Chinese Academy of Sciences

	293T the Cell Bank of the Chinese Academy of Sciences
Authentication	Gibco C11995500BT<br>Gibco C11875500BT<br>Gibco 21127022<br>Gibco 15140122<br>Gibco 35050061<br>Pricella PB180422<br>Gibco A5670701<br>FuHeng FH0183<br>the Cell Bank of the Chinese Academy of Sciences<br>the Cell Bank of the Chinese Academy of Sciences<br>the Cell Bank of the Chinese Academy of Sciences
Mycoplasma contamination	Regular mycoplasma testing were comfirmed
Commonly misidentified lines<br>(See ICLAC register)	None

Animals and other research organisms

Policy information about studies involving animals; ARRIVE guidelines recommended for reporting animal research, and Sex and Gender in Research

Laboratory animals	C57BL/6 mice<br>BALB/c-nu<br>PD-1 mAb<br>Icaritin
Wild animals	This study did not involve wild animals.
Reporting on sex	Gender showed no significant impact on the findings. Within the patient cohort, no gender differences were observed between the bone-metastatic and non-metastatic groups.
Field-collected samples	This study did not involve samples collected from the field.
Ethics oversight	All animal experimental protocols were approved by the Ethics Committee of Nanjing Clinical Medical College, Nanjing University of Chinese Medicine (No. KY20180803-05).

Note that full information on the approval of the study protocol must also be provided in the manuscript.

Plants

Seed stocks	Not Applicable
Novel plant genotypes	Not Applicable
Authentication	Not Applicable

Flow Cytometry

Plots

Confirm that:

☒ The axis labels state the marker and fluorochrome used (e.g. CD4-FITC).

☒ The axis scales are clearly visible. Include numbers along axes only for bottom left plot of group (a 'group' is an analysis of identical markers).

☒ All plots are contour plots with outliers or pseudocolor plots.

☒ A numerical value for number of cells or percentage (with statistics) is provided.

Methodology

Sample preparation	Subcutaneous tumors were excised and minced into pieces. The tissue was digested with an enzyme working solution containing collagenase IV (Shanghai Maokang, MX1004), hyaluronidase (Shanghai Maokang, MX1007), and DNase I (Thermo Fisher, EN0521) at 37°C for 1-2 hours to dissociate the cells. The cell suspension was then passed through a 70 µm cell strainer to remove tissue debris and centrifuged at 800xg for 5 minutes. The cell pellet was resuspended in PBS and subjected to density gradient centrifugation using 30% Percoll (Yeasen,40501ES60). The resulting cells were then incubated with indicated antibodies according to the manufacturer's instructions. Flow cytometry was performed to analyze the CD8+ T or Treg cell positive rate.
Instrument	Backman coulter CytoFLEX
Software	flowjo
Cell population abundance	% Target Population = (Gated Events / Total Acquired Events) × 100%
Gating strategy	Exclude debris/dead cells using FSC/SSC and viability markers;Identify parent population;Subset analysis

☒ Tick this box to confirm that a figure exemplifying the gating strategy is provided in the Supplementary Information.
