# Supplementary material for: YBX1 Confers immunosuppressive bone metastatic traits in non-small cell lung cancer
Source: Nat Commun. 2026 Jun 13;17:7505. doi: 10.1038/s41467-026-73931-2 (PMC13407896; doi:10.1038/s41467-026-73931-2)
Supplement: Supplementary file 3 — Transparent Peer Review file [file 41467_2026_73931_MOESM3_ESM.pdf]

nature portfolio

Peer Review File

Bone metastases derived from lung cancer typically exhibit an immunosuppressive microenvironment. This study identifies a YBX1 glycosylation–dependent mechanism that drives both metastasis and immunosuppression, and proposes the small molecule Icaritin as a potential therapeutic strategy through YBX1 degradation.

Corresponding Author: Professor lilin ge

This file contains all reviewer reports in order by version, followed by all author rebuttals in order by version.

Attachments originally included by the reviewers as part of their assessment can be found at the end of this file.

Version 0:

Reviewer comments:

Reviewer #1

(Remarks to the Author)
The manuscript entitled "YBX1 Confers Immunosuppressive Bone Metastatic Traits in Non-Small Cell Lung Cancer" by Kai Zhang et al. investigates the role of the transcription factor YBX1 in promoting bone metastasis and immunosuppression in non-small cell lung cancer (NSCLC). The authors demonstrate that YBX1 enhances tumor invasiveness and osteoclast activation via IL6 and CCL5 signaling, regulated by O-GlcNAc glycosylation at T271. The small molecule Icaritin (ICA) is identified as a modulator that increases YBX1 glycosylation, promotes its degradation through mitophagy, and suppresses both bone metastasis and immune evasion. The paper offers mechanistic insights and therapeutic implications for YBX1-targeted treatment in NSCLC. Despite the substantial interest and novel mechanistic insights provided by this work, the manuscript still requires additional experimental validation and major revision before it can be considered for publication.

Major Concerns:
1. The limitations of the in vivo metastasis model are as follows:
The utilization of intratibial injection to establish models of bone metastasis constitutes a significant constraint. This approach does not replicate the physiological process of metastasis; rather, it models tumor cell colonization within the bone microenvironment. A more suitable model for substantiating the study's assertions would be a true metastasis model.
2. Selection and characterization of ICA
The rationale behind selecting Icaritin (ICA) as a potential therapeutic agent remains ambiguous and has yet to be substantiated by compelling experimental validation. The present manuscript focuses exclusively on the effects of ICA on YBX1; however, it is highly probable that ICA influences multiple cellular pathways. A more extensive transcriptomic or proteomic analysis is necessary to verify specificity and exclude off-target effects.
3. Figure 1:
-the presence of osteolytic lesions is indicative of a specific mechanism.
-The presented images fail to demonstrate osteolytic lesions with adequate clarity. Quantification by histomorphometry is imperative.
-The relationship between YBX1 and osteoclast activation in the RAW264.7 co-culture remains to be fully substantiated. The assertion of a robust effect predicated on a solitary pathway and a single marker is not substantiated.
4. Figure 2:
-The patient dataset is compelling; however, the addition of bone tissue analyses from metastasis-positive patients would greatly strengthen the translational relevance of the study.
It would be interesting that the extent and severity of bone lesions be thoroughly described and quantified.
-The findings presented in panels F and G offer the preliminary indication of a potential association between the status of the epidermal growth factor receptor (EGFR) mutations and the subject's characteristics. However, this observation remains to

be thoroughly and methodically discussed within the broader context of the study.
5. Figure 3:
-The luciferase reporter assays are exclusively focused on IL6 and CCL5. A more comprehensive and objective analysis, such as ChIP-seq or CUT&RUN, would provide a more robust mechanistic understanding of YBX1's role in transcription.
-The ultimate inferences derived from this data set are of a speculative nature, placing significant emphasis on correlation rather than on the notion of functional causation (panel G-H).
6. Figure 4:
-The immunofluorescence images are of substandard quality, which complicates the evaluation of critical signals.
The statistical significance demonstrated in Panel C is unanticipated based on the low difference observed.
-In order to ensure a valid and reliable comparison, it is essential that all relevant experiments include a control group that has been treated with anti-PD1 alone.
7. Figure 5:
-panels I and J are inadequate in terms of the number of biological replicates. The quantification and reproducibility of the results must be improved.

Minor points:
The graphical abstract is not sufficiently clear, and it must be revised in order to enhance its readability and ensure that it aligns more closely with the primary findings.

Reviewer #2

(Remarks to the Author)
Zhang et al. created high- and low-metastatic, orthotopic NSCLC cell line models to study bone metastasis. By subjecting these cell lines to proteomics analyses, they identified the transcription factor YBX1 as a critical driver of bone metastasis, which was confirmed by transwell migration assays and in a syngeneic mouse model. They also found higher expression of YBX1 in NSCLC patients with bone metastases compared to metastases-free patients. The authors then investigated the molecular basis of YBX1 action: YBX1 underwent LLPS and activated transcription of IL6 and CCL5, which mediated osteoclast activation via RANKL/RANK and recruitment of immunosuppressive Tregs, respectively. Cellular localization and thus transcriptional activity of YBX1 was regulated by O-GlcNAcylation, which lead to nuclear exclusion and mitochondrial translocation. O-GlcNAcylation of YBX1 was also induced by icaritin, a natural flavonoid known for its antitumor effects in various cancers. ICA treatment also lead to autophagic degradation of YBX1.

Taken together, while YBX1 has previously been implicated in cancer biology, this study is novel in demonstrating its dual role in promoting osteolytic bone metastasis and immunosuppression in NSCLC. The authors also provide mechanistic insights suggesting that YBX1 stability is regulated via mitochondrial localization and autophagic degradation. Furthermore, the finding that icaritin enhances YBX1 glycosylation and promotes its degradation, thereby suppressing bone metastasis and modulating anti-tumor immunity, contributes significantly to the study’s translational relevance. However, the mechanistic links between YBX1 glycosylation, mitophagy, and degradation require further validation. Additionally, the clinical relevance of these mechanisms would be strengthened by validation in patient-derived models (e.g., PDXs or organoids).

Specific points:

1. The authors state in the abstract: “Mechanistically, YBX1 protein stability is regulated by glycosylation-dependent mitophagy, wherein O-GlcNAc transferase (OGT)-mediated glycosylation at T271 enhances its mitochondrial membrane localization and subsequent autophagic degradation.” This claim is debatable for two main reasons. First, it is mechanistically counterintuitive to translocate a transcription factor to the mitochondria solely for degradation - why not target it directly to the proteasome or autolysosome? Second, the data in Figure 5 demonstrate that glycosylation promotes mitochondrial localization of YBX1, but do not show that this modification leads to its degradation. Evidence for YBX1 degradation only appears after Icaritin treatment, as shown in Figure 6. Data demonstrating glycosylation-induced autophagic degradation independently of pharmacologic intervention is missing.
2. The proposed mechanism of mitophagic degradation should be corroborated by data on YBX1 half-life, mitophagy flux (LC3-II, p62 turnover) and/or mito-Keima assay.
3. The authors should challenge their conclusions: If T271 glycosylation indeed suppresses YBX1’s nuclear activity, replacing YBX1 wt with YBX1 T271A in A549Low-M cells should phenocopy A549High-M cells, including increased invasion, upregulated IL6/CCL5 expression and increased osteoclast activation.
4. ICA’s specificity for YBX1 versus other OGT substrates should be tested (e.g., glycosylation proteomics) to rule out off-target effects.
5. The authors evaluate the “therapeutic potential of ICA as a YBX1-targeted modulator” in a bone metastasis model in combination with immune check point blockade. However, to corroborate that YBX1 and YBX1 O-GlcNAcylation is critically involved in this process they need to include a YBX1-deficient and a YBX1 T271A-expressing model.
6. The authors should cite and dicuss the article by Zhao et al. (Anticancer Drugs, 2020) which showed that icaritin can inhibit lung cancer-mediated osteoclastogenesis and induce the apoptosis of osteoclasts via the AMPK/mTOR pathway (also describing an effect of ICA on IL-6 and TNF-alpha).
7. The (clinical) impact of the findings would be greatly strengthened by validation in PDX models or functional assays in

human organoid models.
8. Is there a (inverse) correlation of OGT and YBX1 expression levels in metastatic/non-metastatic NSCLC patient samples?
9. Figure 5J: lower panel shows two times YBX1-T271A mutant instead of YBX1 wt and mutant.
10. While the methods are detailed, data presentation (e.g., Western blot band quantification, description of statistical tests, sample sizes, flow cytometry gating strategy, explanation of abbreviations in figures) is not always clear.
11. No mention of raw data availability for proteomics and RNA-seq datasets.

Minor points:

Luci-Lewis cells: purchase information or description of how they were created is missing

Though the manuscript is well written, it contains countless mistakes (typos, missing/incorrect punctuation, grammatical errors, incorrect labelling…) and requires thorough proofreading. Below are some examples, but the list is by no means exhaustive.

• Line 48: (iac) is probably a typo?
• Line 79: superfluous comma after microenvironment.
• Lines 365/366: Unclear sentence "Through co-culturing different rounds of cells and RAW264.7 cells following osteoclast activity measurement."
• Line 421: incomplete and grammatically incorrect sentence
• Lines 431-433: grammatically incorrect sentence
• Lines 508 – 509: incomplete sentence
• Legend Figure 6 (lines 825-26): C. appears a second time with a caption that does not fit to the figure…
• Figure 1: "D" is missing in the figure…

Reviewer #3

(Remarks to the Author)
This manuscript, YBX1 confers immunosuppressive bone metastatic traits in NSCLC, by Kai Zhang, et al. indicates that YBX1 is highly glycosylation and increases its degradation through mitophagy, thereby decreases the expression of IL6 and CCL5, leading immunosuppressive bone metastatic traits. In addition, they also identified a specific inhibitor, ICA, which can inhibit the glycosylation of YBX1 to reverse immune activity, resulting in inhibition of cancer metastasis. Understanding the mechanisms of how to induce resistance during immunotherapy is crucial and clinical unmet now. Several suggestion listed here hope to be beneficial for this study.

Comments
1. In Fig.3, although the authors used LLPS to study the nuclear localization, this results can't support that YBX1 can specific recruitment to the promoters of IL6 and CCL5. So, it will be better to use ChIP to support it.
2. In Fig.4B, the pattern of FOXP3 and CD25 is very similar, which needs to be confirmed.
3. In Fig.4D, it will be better to show the real tumor, not show a cartoon figure.
4. In Fig.5D, the OGT input needs to be confirmed, why only found in GST-YBX1/His-OGT expression but not in His-OGT only?
5. In Fig.5E, the level of YBX1 should be decreased under Myc-OGT expression condition. However, the level of YBX1 is not decreased but slightly increase in Myc-OGT overexpression condition.
6. In Fig.5F, it will be better to also show the YBX1 input level.
7. In Fig.5H, Flag-YBX1 and its mutant should be show in the INPUT as an internal control.
8. In the Fig.5, it will be better to run the samples in the same gel, not in the different gel.
9. In Fig.5J, it will be better to compare the mitochondria colocalization in A549 high M and low M.
10. What is the inhibitory mechanism of ICA?
11. In Fig.6G, the ICA will inhibit YBX1 glycoylation, the YBX1 should be higher than NC control, but the level of YBX1 is lower than control, why?
12. In Fig.6J, so many similar colors were show here, which is difficult to review. In addition, why only used higher M cells to study this experiment? It will be better to also use low M to address this issue, thereby compare the difference between high M and low M. In addition, about the evidence to support ICA-induced mitophagy is not enough. More mitophagy-associated markers should be used to confirm this conclusion.
13. In Fig.7, the dose and therapeutic protocol should be described in the figure legend. In addition, it will be better to show anti-PD-1 treated alone.
14. Some English spell is found in the text, like iac in the abstract.
Overall, the finding is novelty. However, many results need to be performed to improve the result's quality and to support the conclusion such as mitophagy and mitochondrial colocalization. Final, it will be better to show the clinical relevance among YBX1, OGT and survival rate.

Reviewer #4

(Remarks to the Author)

Zhang, Kai et al. YBX1 Confers Immunosuppressive Bone Metastatic Traits in Non-Small Cell Lung Cancer.

This manuscript shows that the transcription factor YBX1 drives both bone metastasis and the development of an immunosuppressive microenvironment in Non-Small Cell Lung Cancer (NSCLC). YBX1 transcriptionally activates IL6 and CCL5, which promotes metastasis and immune evasion in highly bone-metastatic NSCLC cells. Furthermore, YBX1 is regulated by O-GlcNAcylation at T271, which redirects it to mitochondria for mitophagic degradation and, therefore, limits its nuclear transcriptional activity. In highly bone-metastatic cells, reduced O-GlcNAcylation allows YBX1 accumulation and enhanced IL6/CCL5 signaling. Through small-molecule screening, the authors identify Icaritin (ICA) as a compound that enhances YBX1 O-GlcNAcylation. Authors show that ICA treatment alone reduces bone metastasis, and sensitizes tumors to immunotherapy. Overall, these findings establish YBX1 as a dual-functional therapeutic target for preventing NSCLC bone metastasis and regulating immunotherapy response.
Overall, this paper provides novel mechanistic insight into YBX1 regulation and its role in NSCLC bone metastasis. However, several points require clarification to strengthen the functional connection to regulation by O-GlcNac related to bone metastasis.

Major Points:
1. The role of YBX1 O-GlcNAcylation is interesting. However, it is not well connected to role of YBX1 in bone metastasis. Authors should test whether re-expression of wildtype but not T271A mutant rescues bone metastatic effect shown in sgYBX1 cells in Fig. 1H. Also, not well connected to whether increased O-GlcNAcylation regulates YBX1 degradation (is mutant more stable?) Authors should test whether increasing O-GlcNAcylation (use OGA inhibitor) induces same effect on YBX1 levels and stability as seen with ICA..
2. Not clear what cells are being used for Fig. 4C-F (not mentioned in text or Figure Legend). Are these mouse NSCLC cells? Is this a syngeneic model?
3. Figure 5C IP should be done in one gel to compare High vs Low cells and allow authors to conclude that "OGT-YBX1 interaction is stronger in Low vs High cells". Same for comparing levels of O-GlcNAc and YBX1 O-GlcNAcylation in Fig. 6F and 6G.
4. Figure 5J staining is difficult to see. Since this is a major conclusion of manuscript, authors should provide more convincing data that O-GlcNAcylation at T271 redirects YBX1 to mitochondria (compare cytosol vs mitochondrial fractions).
5. The conclusion that "ICA as an effective inhibitor targeting YBX1-T271 O-GlcNAcylation to remodel YBX1 localization and functional activity" needs further support including functional relevance of T271 (see point 1). More likley ICA either regulates broad pathways including global O-GlcNAcylation thus authors should clearly test whether ICA regulates total O-GlcNAcylation, OGT and OGA levels.
6. Fig. 7A its difficult to see any difference between control and ICA treated mice representative luciferase image. Many references not included: Pg. 9, "Prior studies suggest that YBX1 undergoes…" – Pg. 9, "Prior studies indicate that tumor-derived…" P 14, "Given the established link between protein glycosylation and membrane organellelocalization..".
7. Figure 5G – Distinction between red and black is difficult to interpret. Highlight T271 more clearly.
8. Figure 6D-E – Statistical significance is mentioned, but no quantification is provided.

Minor Points:
1. Abstract – Update Icaritin abbreviation from iac to ICA to match the rest of the paper
2. Graphical Abstract – Bone metastasis representation should be larger.
3. Figure 1C – Clarify what bone is being shown (tibia?)
4. Authors state "Western blot assay proved that the protein level of OGT is higher in A549Low-M cells that that in A549-High-M cells" but don't reference Figure (Fig. 5B). Also, Pg. 8, "Transcriptional reprogramming plays…" – Run-on sentence; split this into two. Add period after "bone metastasis" and begin new sentence with "Thus, we performed…" for clarity.
5. Figure 2E – Some numbers in chart on the bottom of the panel are cut off or unclear.
6. Figure 2F – Clarify what channels the first merge represent.
7. Pg. 12 – Delete "3" in front of subheading "YBX1 transcriptionally activates…" to match subheading format throughout results section.
8. Did Icaritin display any off-target effects or toxicities?

Reviewer #5

(Remarks to the Author)
I co-reviewed this manuscript with one of the reviewers who provided the listed reports. This is part of the Nature Communications initiative to facilitate training in peer review and to provide appropriate recognition for Early Career Researchers who co-review manuscripts.

Version 1:

Reviewer comments:

Reviewer #1

(Remarks to the Author)
Thanks for the revision of the manuscript, the authors have fulfilled all the requests and I have no further comments.

Reviewer #2

(Remarks to the Author)
The authors have included several new experiments to support their conclusions. While some of the responses and added experiments are convincing, others are not. For example, the replacement experiment (new Figure S6A-D) wasn't done properly. Instead of replacing endogenous YBX1 with (equal levels of) the mutant, the authors vastly overexpressed the FLAG-tagged mutant. This set of experiment cannot be interpreted in a meaningful way and should be removed from the paper.
In Figure 8, the authors show new experiments addressing the therapeutic potential of ICA. They state that sgYBX1 potentiates the effect of a combined PD-1 inh./ ICA treatment (line 612). This is not the case – in none of the tested settings. Also, the YBX1 mutant, which cannot be O-GlcNAcylated and should be therefore more stable and thus more "metastasizing" as the wt, behaves exactly like YBX1 wt. Why is that? In addition, the authors need to show equal expression levels of endogenous YBX1, YBX1 wt and YBX1 mut. Otherwise the results are hard to interpret.
Moreover, the newly presented data in Figure 5 and 6 contain multiple errors (see below), raising general concerns about the overall scientific rigor within the study. So, while I do believe that the main message (role of O-GlcNAcylation for YBX1 stability and effect of ICA on YBX1 localisation and stabilty) is probably correct and interesting, the revised manuscript is unfortunately not accepable in its current form.

Mistakes in data presentation:
Figure 5I: The figure is obviously mislabelled (YBX1 T271-Mut should be WT) and the WB incorrectly quantified:

Figure 6E-F: Description in the figure legend does not fit to the shown data. Moreover, in 6E the applied ICA dose is missing in left panel, while incubation time is missing in the right panel.
Figure 6G: figure legend is wrong.

Minor:
Define abbreviation OGA

Reviewer #3

(Remarks to the Author)
All the concerns in the previous version have been improved and response in the currently revised drift. I have no suggestion.

Reviewer #4

(Remarks to the Author)

Authors have addressed mall major and minor issues and thus acceptable for publication.

Reviewer #5

(Remarks to the Author)
I co-reviewed this manuscript with one of the reviewers who provided the listed reports. This is part of the Nature Communications initiative to facilitate training in peer review and to provide appropriate recognition for Early Career Researchers who co-review manuscripts.

Version 2:

Reviewer comments:

Reviewer #2

(Remarks to the Author)
The authors have addressed my concerns and I have no objections further.

Open Access This Peer Review File is licensed under a Creative Commons Attribution 4.0 International License, which permits use, sharing, adaptation, distribution and reproduction in any medium or format, as long as you give appropriate credit to the original author(s) and the source, provide a link to the Creative Commons license, and indicate if changes were made.
In cases where reviewers are anonymous, credit should be given to 'Anonymous Referee' and the source.
The images or other third party material in this Peer Review File are included in the article's Creative Commons license, unless indicated otherwise in a credit line to the material. If material is not included in the article's Creative Commons license and your intended use is not permitted by statutory regulation or exceeds the permitted use, you will need to obtain permission directly from the copyright holder.
To view a copy of this license, visit https://creativecommons.org/licenses/by/4.0/

REVIEWER COMMENTS

Reviewer #1 (Remarks to the Author):

The manuscript entitled "YBX1 Confers Immunosuppressive Bone Metastatic Traits in Non-Small Cell Lung Cancer" by Kai Zhang et al. investigates the role of the transcription factor YBX1 in promoting bone metastasis and immunosuppression in non-small cell lung cancer (NSCLC). The authors demonstrate that YBX1 enhances tumor invasiveness and osteoclast activation via IL6 and CCL5 signaling, regulated by O-GlcNAc glycosylation at T271. The small molecule Icaritin (ICA) is identified as a modulator that increases YBX1 glycosylation, promotes its degradation through mitophagy, and suppresses both bone metastasis and immune evasion. The paper offers mechanistic insights and therapeutic implications for YBX1-targeted treatment in NSCLC. Despite the substantial interest and novel mechanistic insights provided by this work, the manuscript still requires additional experimental validation and major revision before it can be considered for publication.

Major Concerns:
1. The limitations of the in vivo metastasis model are as follows:
The utilization of intratibial injection to establish models of bone metastasis constitutes a significant constraint. This approach does not replicate the physiological process of metastasis; rather, it models tumor cell colonization within the bone microenvironment. A more suitable model for substantiating the study's assertions would be a true metastasis model.

Response: Thank you very much for your valuable suggestions. Our focus is on how the characteristics of tumor cells remodel the colonization microenvironment. The review titled "Biology, vulnerabilities and clinical applications of circulating tumor cells" (https://doi.org/10.1038/s41568-022-00536-4) published in *Nature Reviews Cancer* summarizes recent advances in the study of CTC biological characteristics. It reports that up to 1×10^6 cancer cells per gram of tumor tissue can enter the circulation, yet the metastatic efficiency of CTCs appears to be very low, approximately 0.01%. Active remodeling of the colonization microenvironment by tumor cells is a critical step for successful metastasis, with the core significance of this remodeling being the "education of the soil" for metastasis. The model we constructed is designed to analyze the role of tumor cells in remodeling the bone metastasis microenvironment in lung cancer bone metastasis. We initially attempted orthotopic lung implantation, but the mice died before bone metastasis occurred. Therefore, we used an intratibial injection model to evaluate the bone metastasis process. This model is widely used in cancer bone metastasis studies, and a 2019 study published in *Cell* employed this model to analyze how changes in the colonization microenvironment affect tumor metastasis (https://doi.org/10.1016/j.cell.2019.10.029). In our future research, we will further refine and enrich our study models.

2. Selection and characterization of ICA
The rationale behind selecting Icaritin (ICA) as a potential therapeutic agent remains ambiguous and has yet to be substantiated by compelling experimental validation. The present manuscript focuses exclusively on the effects of ICA on YBX1; however, it is highly probable that ICA

influences multiple cellular pathways. A more extensive transcriptomic or proteomic analysis is necessary to verify specificity and exclude off-target effects.

Response: Thank you for your suggestion. We selected ICA based on the following rationale: First, we screened a compound library using a reporter system designed to evaluate the transcriptional activity of YBX1. From this screen, we identified three compounds-Elemicin, Erianin, and ICA-that most significantly inhibited YBX1-Motif activity, with ICA showing the strongest effect. Second, we treated A549^{High-M} cells with these three compounds and collected the conditioned medium to culture RAW264.7 cells. TRAP staining was performed to assess the effect of the tumor cell-conditioned medium on osteoclast activity. The results demonstrated that the supernatant from ICA-treated cells most potently suppressed osteoclast viability.

To further substantiate our findings, we performed transcriptomic analysis on ICA-treated cells. This revealed significant downregulation of CCL5 and IL6 among the differentially expressed genes, indicating that ICA and YBX1 knockdown modulate overlapping molecular pathways. This consistency strongly supports the rationale for selecting ICA as a YBX1-targeting agent.

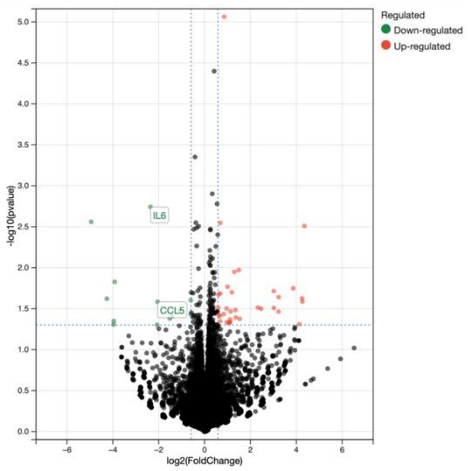

3. Figure 1:
-the presence of osteolytic lesions is indicative of a specific mechanism.
-The presented images fail to demonstrate osteolytic lesions with adequate clarity. Quantification by histomorphometry is imperative.
-The relationship between YBX1 and osteoclast activation in the RAW264.7 co-culture remains to be fully substantiated. The assertion of a robust effect predicated on a solitary pathway and a single marker is not substantiated.

Response: Thank you for your suggestions. The quantitative results for BV/TV (bone volume fraction), BS/TV (bone surface area per tissue volume), Tb.N (trabecular number), and Tb.Sp (trabecular separation) are presented in **Figure 1D**. During the establishment of our model, BV/TV, BS/TV, and Tb.N gradually decreased, while Tb.Sp increased. Following YBX1 knockdown, BV/TV, BS/TV, and Tb.N increased, whereas Tb.Sp decreased, suggesting a reduction in osteolytic destruction (**Figure 1J**).

Furthermore, we analyzed the correlation between YBX1 expression and osteoclast activity, which showed a positive correlation: higher YBX1 expression in A549 cells was associated with greater activation of osteoclasts (**Figure 1H)**. Additionally, we examined key molecules associated with bone metastasis, such as RANKL, Integrin β3, MMP9, CTSK, and DC-STAMP. The expression of these factors was suppressed after YBX1 interference, supporting our conclusions. These findings are presented in **Figure S1C–F**.

4. Figure 2:
-The patient dataset is compelling; however, the addition of bone tissue analyses from metastasis-positive patients would greatly strengthen the translational relevance of the study.
It would be interesting that the extent and severity of bone lesions be thoroughly described and quantified.
-The findings presented in panels F and G offer the preliminary indication of a potential association between the status of the epidermal growth factor receptor (EGFR) mutations and the subject's characteristics. However, this observation remains to be thoroughly and methodically discussed within the broader context of the study.

Response: Thank you for your valuable feedback. As this is a retrospective study and considering the ethical and practical challenges associated with routine biopsy of bone metastases in clinical practice, we were unable to obtain bone tissue samples from the patients. While direct analysis of bone tissue was not feasible, we fully agree with your perspective. Therefore, we re-reviewed the imaging data of all patients with bone metastases and compiled the results from their bone scans. The image below shows a representative bone scan selected from our cohort, while the complete set of 118 bone scan results, along with their corresponding metastatic sites, is provided in **Supplementary File 1**.

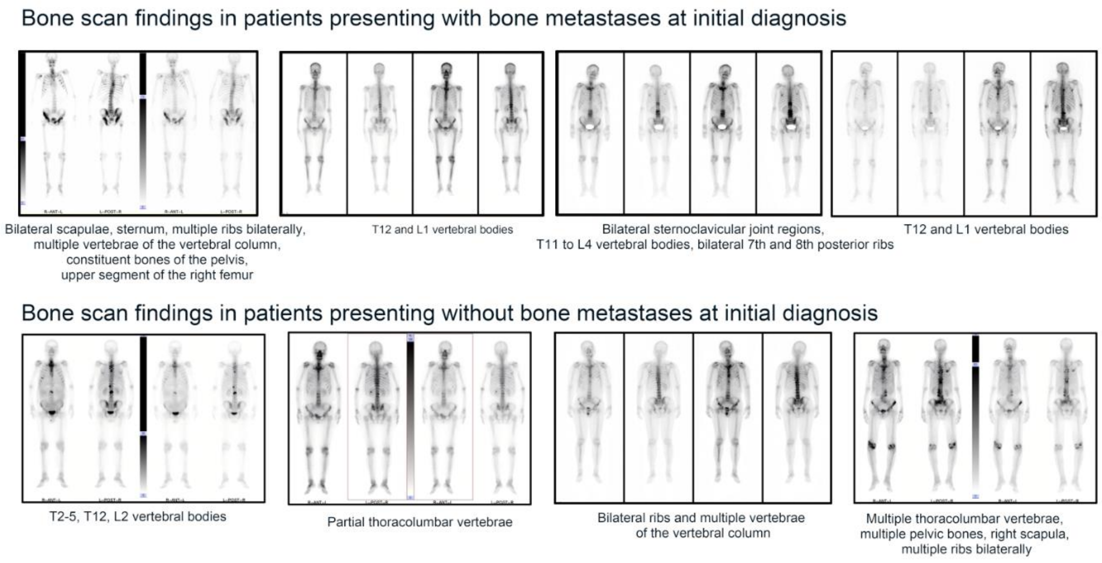

Furthermore, regarding EGFR, studies have shown that it not only drives tumor cell proliferation and survival but also acts as a key orchestrator of tumor invasion and metastasis. Its hyperactivation

initiates complex molecular programs that enable cancer cells to migrate, invade, evade anoids, and seed distant organs. The metastatic cascade is a multi-step process, and EGFR plays critical roles at various stages, including inducing epithelial-mesenchymal transition (EMT), promoting angiogenesis, resisting anoikis, facilitating extravasation, and establishing metastatic niches (PMID: 29462253, PMID: 24419416). We have addressed these aspects in the corresponding sections of the results.

5. Figure 3:

-The luciferase reporter assays are exclusively focused on IL6 and CCL5. A more comprehensive and objective analysis, such as ChIP-seq or CUT&RUN, would provide a more robust mechanistic understanding of YBX1's role in transcription.

-The ultimate inferences derived from this data set are of a speculative nature, placing significant emphasis on correlation rather than on the notion of functional causation (panel G-H).

Response: Thank you for your valuable suggestions. We performed ChIP-seq assays to analyze the global transcriptional regulation profile of YBX1 and specifically extracted the peak profiles for IL6 and CCL5. The related results are shown in the below (**Figure S3A-C**).

Regarding panels G-H, we supplemented the study with experiments treating with an IL-6 neutralizing antibody to assess its effect on osteoclasts. The results showed that after inhibiting IL-6, the activating effect of A549$^{High\text{-}M}$ cells on osteoclasts was reduced, indicating that IL-6 can promote osteoclast activation. These results are presented in **Figure 3I**.

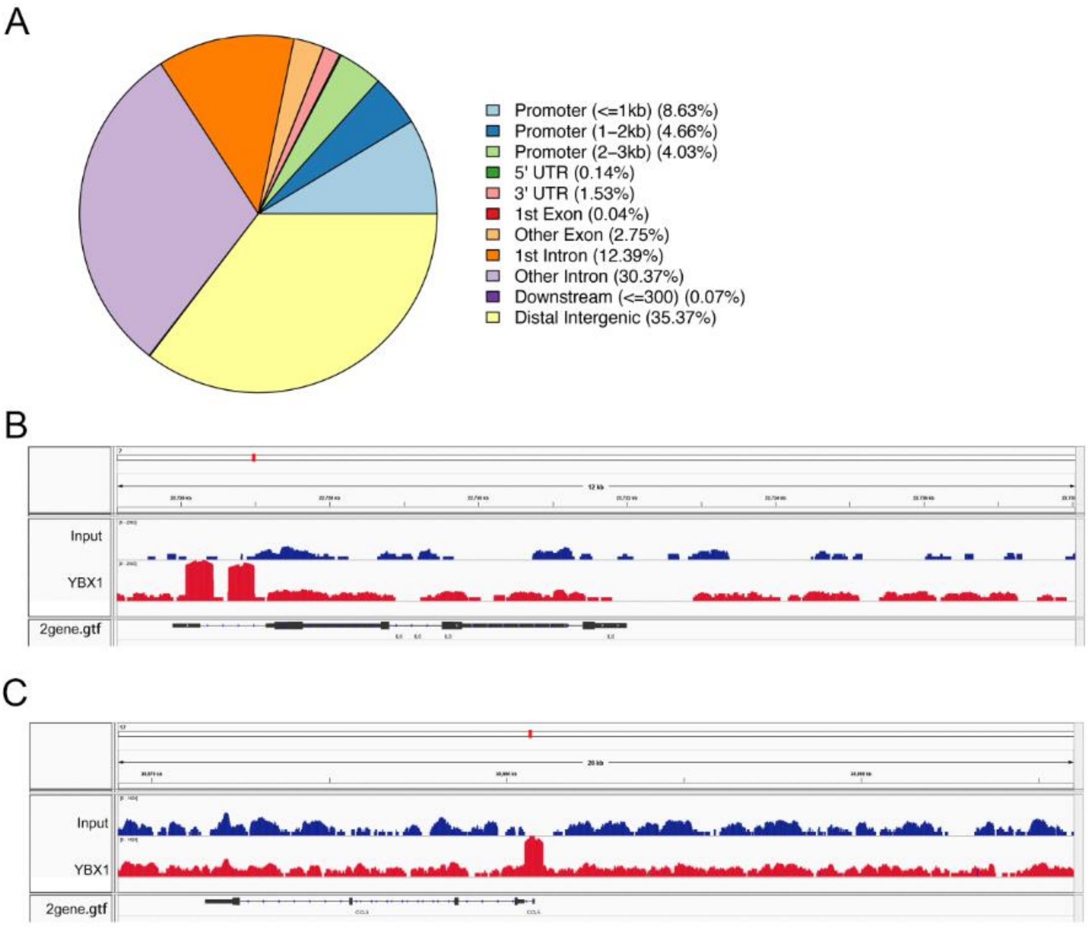

6. Figure 4:

-The immunofluorescence images are of substandard quality, which complicates the evaluation of critical signals.

The statistical significance demonstrated in Panel C is unanticipated based on the low difference observed.

-In order to ensure a valid and reliable comparison, it is essential that all relevant experiments include a control group that has been treated with anti-PD1 alone.

Response: We are grateful for your valuable recommendations. In accordance with your suggestions, the corresponding result panels have been updated to feature high-resolution representative images, and the quantitative analysis for **Figure 4B-C** has been performed again. Accordingly, the animal experiment was also repeated, this time including supplementary groups for IgG control and PD-1 mAb. The results from these additional investigations are provided in **Figure 4D-G**.

7. Figure 5:

-panels I and J are inadequate in terms of the number of biological replicates. The quantification and reproducibility of the results must be improved.

Response: Thank you for your valuable suggestion. We have conducted multiple independent

replicate experiments. Due to the addition of new data, the figure order has been adjusted. The corresponding statistical results for the relevant data have been updated and are now provided in **Figure 5L-M**.

Minor points:

The graphical abstract is not sufficiently clear, and it must be revised in order to enhance its readability and ensure that it aligns more closely with the primary findings.

Response: We appreciate your valuable suggestion. In accordance with your comment, we have revised the graphical abstract, which is presented below.

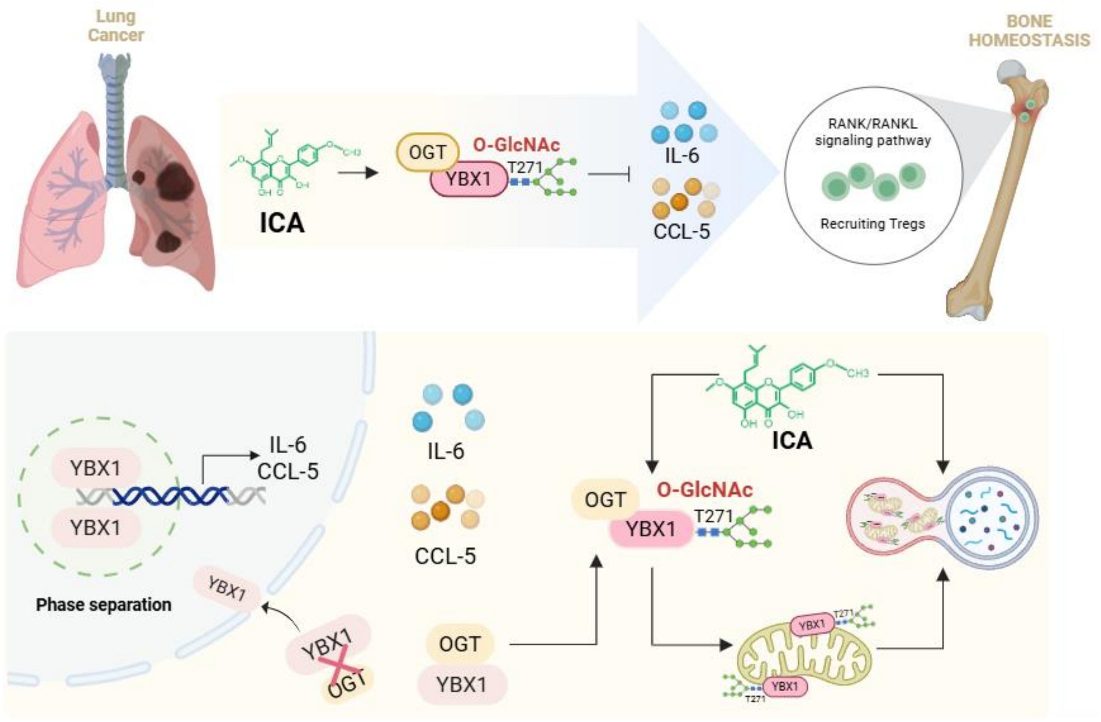

Reviewer #2 (Remarks to the Author):

Zhang et al. created high- and low-metastatic, orthotopic NSCLC cell line models to study bone metastasis. By subjecting these cell lines to proteomics analyses, they identified the transcription factor YBX1 as a critical driver of bone metastasis, which was confirmed by transwell migration assays and in a syngeneic mouse model. They also found higher expression of YBX1 in NSCLC patients with bone metastases compared to metastases-free patients. The authors then investigated the molecular basis of YBX1 action: YBX1 underwent LLPS and activated transcription of IL6 and CCL5, which mediated osteoclast activation via RANKL/RANK and recruitment of immunosuppressive Tregs, respectively. Cellular localization and thus transcriptional activity of YBX1 was regulated by O-GlcNAcylation, which lead to nuclear exclusion and mitochondrial translocation. O-GlcNAcylation of YBX1 was also induced by icaritin, a natural flavonoid known for its antitumor effects in various cancers. ICA treatment also lead to autophagic degradation of YBX1.

Taken together, while YBX1 has previously been implicated in cancer biology, this study is novel in demonstrating its dual role in promoting osteolytic bone metastasis and immunosuppression in NSCLC. The authors also provide mechanistic insights suggesting that YBX1 stability is regulated via mitochondrial localization and autophagic degradation. Furthermore, the finding that icaritin enhances YBX1 glycosylation and promotes its degradation, thereby suppressing bone metastasis and modulating anti-tumor immunity, contributes significantly to the study's translational relevance. However, the mechanistic links between YBX1 glycosylation, mitophagy, and degradation require further validation. Additionally, the clinical relevance of these mechanisms would be strengthened by validation in patient-derived models (e.g., PDXs or organoids).

Response: We appreciate your valuable comments. They have been carefully incorporated into our revised manuscript.

Specific points:

1. The authors state in the abstract: "Mechanistically, YBX1 protein stability is regulated by glycosylation-dependent mitophagy, wherein O-GlcNAc transferase (OGT)-mediated glycosylation at T271 enhances its mitochondrial membrane localization and subsequent autophagic degradation." This claim is debatable for two main reasons. First, it is mechanistically counterintuitive to translocate a transcription factor to the mitochondria solely for degradation - why not target it directly to the proteasome or autolysosome? Second, the data in Figure 5 demonstrate that glycosylation promotes mitochondrial localization of YBX1, but do not show that this modification leads to its degradation. Evidence for YBX1 degradation only appears after Icaritin treatment, as shown in Figure 6. Data demonstrating glycosylation-induced autophagic degradation independently of pharmacologic intervention is missing.

Response: Thank you for your valuable suggestions. Previous studies have confirmed that YBX1 localizes to mitochondria, and this localization is associated with its role in regulating glycolysis (https://doi.org/10.1093/lifemeta/load038). In our study, we found that its mitochondrial localization is related to glycosylation modification. We believe that glycosylated YBX1 enters the

mitochondria to perform certain regulatory functions, which is a dynamic physiological process. Once its function is fulfilled, it is degraded along with mitophagy. ICA, on the other hand, accelerates the degradation of YBX1 by enhancing mitophagy. To provide more compelling evidence, we conducted the following additional experiments during the revision process: (1) We isolated mitochondria from high- and low-metastatic cells and performed Western blot validation, along with the addition of deglycosylation enzyme (OGA). The results showed that the YBX1 level in mitochondria of high-metastatic cells was significantly lower than in low-metastatic cells. After adding OGA, the YBX1 level in mitochondria of low-metastatic cells also decreased, confirming that glycosylation modification promotes mitochondrial localization (**Figure 5I**). (2) During the experiments, we also observed that mitophagy occurs in both high- and low-metastatic cells. After treatment with ICA, the level of mitophagy significantly increased (**Figure 6I**).

2. The proposed mechanism of mitophagic degradation should be corroborated by data on YBX1 half-life, mitophagy flux (LC3-II, p62 turnover) and/or mito-Keima assay.

Response: Thank you for your valuable suggestions. The half-life of YBX1 is provided in **Figure 6G**. Furthermore, we conducted additional experiments and demonstrated that the level of YBX1 gradually decreases with the increase of LC3-II (or the reduction of p62). These findings are presented in **Figure 6H**.

3. The authors should challenge their conclusions: If T271 glycosylation indeed suppresses YBX1's nuclear activity, replacing YBX1 wt with YBX1 T271A in A549Low-M cells should phenocopy A549High-M cells, including increased invasion, upregulated IL6/CCL5 expression and increased osteoclast activation.

Response: Thank you for your valuable suggestions. Based on your advice, we conducted additional experiments. The results confirmed that the T271 site mutation can mimic the function of $A549^{High\text{-}M}$ cells, meaning it exhibits stronger invasion and osteoclast activation capabilities, and can promote the expression of IL6 and CCL5. The related results are presented in **Figure S6A-D**.

4. ICA's specificity for YBX1 versus other OGT substrates should be tested (e.g., glycosylation proteomics) to rule out off-target effects.

Response: Thank you for your valuable suggestions. Our experiments revealed that ICA does not increase the global O-GlcNAcylation level, nor does it alter the levels of OGT or OGA (**Figure 7B**). However, it promotes the binding between OGT and YBX1 (**Figure 7C**), suggesting that ICA likely enhances the O-GlcNAcylation of YBX1 by facilitating their interaction, indicating a certain degree of specificity. Furthermore, following the latest research trends, we selected a protein (EGR2) already confirmed to undergo OGT-mediated O-GlcNAcylation for validation. (A research article titled "EGR2 O-GlcNAcylation Orchestrates the Development of Protumoral Macrophages to Limit $CD8^+$ T Cell Antitumor Responses", published in June 2025 in the journal *Cell Chemical Biology*,

confirmed that EGR2 can be O-GlcNAcylated by OGT, PMID: 40499546). Our analysis showed that ICA does not affect the O-GlcNAcylation of this EGR2 protein by OGT, further supporting the specificity of ICA's effect on YBX1 (**Figure 7D**). However, we cannot rule out the possibility of other functions, and we will continue to refine this research in the future.

5. The authors evaluate the "therapeutic potential of ICA as a YBX1-targeted modulator" in a bone metastasis model in combination with immune check point blockade. However, to corroborate that YBX1 and YBX1 O-GlcNAcylation is critically involved in this process they need to include a YBX1-deficient and a YBX1 T271A-expressing model.

Response: Thank you for your valuable suggestions. Based on your advice, we have supplemented the corresponding modeling results, which are now presented in **Figure 8**. With the refined grouping, it further confirms that the YBX1 T271 site is critical for ICA's mechanism of action.

6. The authors should cite and dicuss the article by Zhao et al. (Anticancer Drugs, 2020) which showed that icaritin can inhibit lung cancer-mediated osteoclastogenesis and induce the apoptosis of osteoclasts via the AMPK/mTOR pathway (also describing an effect of ICA on IL-6 and TNF-alpha).

Response: Thank you for your valuable suggestion. As you recommended, we analyzed the study by Zhao et al. (Anticancer Drugs, 2020). Their research demonstrated that ICA can inhibit lung cancer-mediated osteoclastogenesis and induce osteoclast apoptosis by activating the AMPK/mTOR signaling pathway, while also downregulating IL-6 and TNF-α levels.

More importantly, our study reveals its direct target within the tumor cells themselves—YBX1. We found that ICA suppresses the production of pro-metastatic factors like IL-6 at the source by promoting the O-GlcNAcylation and subsequent degradation of YBX1. This provides an upstream mechanistic explanation for the decreased IL-6 levels observed by Zhao et al. Our research extends the understanding of ICA's mechanism of action from tumor microenvironment modulation to a fundamental reversal of the "metastatic reprogramming" within the tumor cells themselves. We have included relevant discussion on this in the Discussion section.

7. The (clinical) impact of the findings would be greatly strengthened by validation in PDX models or functional assays in human organoid models.

Response: Thank you for your valuable suggestion. Currently, regarding the lung cancer bone metastasis context involved in this study, we have not been able to identify suitable, established PDX or organoid models. We will continue to monitor advances in modeling within this field and endeavor to conduct validation using new models in subsequent work.

8. Is there a (inverse) correlation of OGT and YBX1 expression levels in metastatic/non-metastatic NSCLC patient samples?

Response: Thank you for your valuable suggestion. We analyzed the correlation between OGT and

YBX1 in clinical tissue samples. The results showed that the expression of OGT was significantly lower in metastatic tissues compared to non-metastatic tissues. Furthermore, in both metastatic and non-metastatic tissues, the expression of OGT was negatively correlated with the expression of YBX1. These related results are presented in **Figure S5C-E**.

9. Figure 5J: lower panel shows two times YBX1-T271A mutant instead of YBX1 wt and mutant.
Response: Thank you for your valuable suggestion. We sincerely apologize, and we have already conducted a thorough check of the manuscript.

10. While the methods are detailed, data presentation (e.g., Western blot band quantification, description of statistical tests, sample sizes, flow cytometry gating strategy, explanation of abbreviations in figures) is not always clear.
Response: Thank you for your valuable suggestions. We have thoroughly revised and elaborated on the Materials and Methods section.

11. No mention of raw data availability for proteomics and RNA-seq datasets.
Response: Thank you for your valuable suggestions. The relevant raw data has been submitted to the linked website (https://ngdc.cncb.ac.cn/).

Minor points:

Luci-Lewis cells: purchase information or description of how they were created is missing

Though the manuscript is well written, it contains countless mistakes (typos, missing/incorrect punctuation, grammatical errors, incorrect labelling…) and requires thorough proofreading. Below are some examples, but the list is by no means exhaustive.

• Line 48: (iac) is probably a typo?
• Line 79: superfluous comma after microenvironment.
• Lines 365/366: Unclear sentence "Through co-culturing different rounds of cells and RAW264.7 cells following osteoclast activity measurement."
• Line 421: incomplete and grammatically incorrect sentence
• Lines 431-433: grammatically incorrect sentence
• Lines 508 – 509: incomplete sentence
• Legend Figure 6 (lines 825-26): C. appears a second time with a caption that does not fit to the figure…
• Figure 1: "D" is missing in the figure…

Response: Thank you for your valuable suggestions. We sincerely apologize for the writing and grammatical errors in the manuscript. We have thoroughly reviewed and revised the entire manuscript. Once again, we greatly appreciate your insightful comments.

Reviewer #3 (Remarks to the Author):

This manuscript, YBX1 confers immunosuppressive bone metastatic traits in NSCLC, by Kai Zhang, et al. indicates that YBX1 is highly glycosylation and increases its degradation through mitophagy, thereby decreases the expression of IL6 and CCL5, leading immunosuppressive bone metastatic traits. In addition, they also identified a specific inhibitor, ICA, which can inhibit the glycosylation of YBX1 to reverse immune activity, resulting in inhibition of cancer metastasis. Understanding the mechanisms of how to induce resistance during immunotherapy is crucial and clinical unmet now. Several suggestion listed here hope to be beneficial for this study.

Comments

1. In Fig.3, although the authors used LLPS to study the nuclear localization, this results can't support that YBX1 can specific recruitment to the promoters of IL6 and CCL5. So, it will be better to use ChIP to support it.

Response: Thank you for your valuable suggestions. We performed ChIP-seq experiments using a YBX1 antibody. The sequencing results demonstrated that YBX1 enriches DNA fragments in the promoter regions of IL6 and CCL5 (**Figure S3A-C**). This finding was further validated by ChIP assays, which confirmed the binding of YBX1 to the promoter regions of IL6 and CCL5. The related experimental results have been provided in Supplementary **Figure S3D**.

2. In Fig.4B, the pattern of FOXP3 and CD25 is very similar, which needs to be confirmed.

Response: Thank you for your valuable suggestions. We have re-analyzed the expression of related molecules in the tissues, and the new results are presented in **Figure 4B**.

3. In Fig.4D, it will be better to show the real tumor, not show a cartoon figure.

Response: Thank you for your valuable suggestions. We have provided actual tumor tissue, and the related results are presented in **Figure 4D**.

4. In Fig.5D, the OGT input needs to be confirmed, why only found in GST-YBX1/His-OGT expression but not in His-OGT only?

Response: Thank you for your valuable suggestions. We have repeated the experiments, and the related results are presented in **Figure 5D.**

5. In Fig.5E, the level of YBX1 should be decreased under Myc-OGT expression condition. However, the level of YBX1 is not decreased but slightly increase in Myc-OGT overexpression condition.

Response: Thank you for your suggestion! You are absolutely right—the YBX1 level in the overexpression group should indeed be lower. We repeated the experiment, and the results confirmed that YBX1 is reduced following OGT overexpression. The related findings are presented in **Figure 5E**.

6. In Fig.5F, it will be better to also show the YBX1 input level.

Response: Thank you for your suggestion. We have repeated the Western blot and supplemented the YBX1 (HA) results in the Input section. The related results are presented in **Figure 5F**.

7. In Fig.5H, Flag-YBX1 and its mutant should be show in the INPUT as an internal control.

Response: Thank you for your suggestion. We have repeated the relevant experiments and supplemented the results of Flag-YBX1 in the Input section. The related results are presented in **Figure 5H**.

8. In the Fig.5, it will be better to run the samples in the same gel, not in the different gel.

Response: Thank you for your suggestion. We have repeated the Western blot to present the results on the same gel, as detailed in **Figure 5** and **Supplementary File 2**.

9. In Fig.5J, it will be better to compare the mitochondria colocalization in A549 high M and low M.

Response: Thank you for your suggestion. We have compared the co-localization of YBX1 with mitochondria in cells with high and low metastatic potential. The results show that the level of YBX1 co-localization with mitochondria is relatively higher in low-metastatic cells compared to high-metastatic cells. For details, please see **Figure 5K** and **Figure S7A**.

10. What is the inhibitory mechanism of ICA?

Response: Thank you for your suggestion! In our study, we found that ICA does not increase global O-GlcNAcylation levels and has no effect on the expression of OGT or OGA (**Figure 7B**). However, it significantly promotes the binding between YBX1 and OGT, while showing no significant impact on the O-GlcNAcylation of other known modified proteins (**Figure 7C-D**). This indicates that ICA primarily functions by specifically enhancing the interaction between OGT and YBX1, strengthening their binding affinity, and thereby promoting YBX1 O-GlcNAcylation. This modification subsequently induces its mitochondrial translocation, ultimately leading to its degradation via ICA-enhanced mitophagy. The detailed mechanistic insights underlying this process will be a key focus of our future research.

11. In Fig.6G, the ICA will inhibit YBX1 glycoylation, the YBX1 should be higher than NC control, but the level of YBX1 is lower than control, why?

Response: Thank you for your suggestion! Our study revealed that ICA promotes the O-GlcNAcylation of YBX1. The glycosylated YBX1 then translocate to mitochondria and could be degraded through mitophagy. ICA enhances mitophagy, thereby facilitating the degradation of YBX1. In our previously provided results, the samples were run on separate gels and without BafA1

treatment. To ensure greater rigor, we have now conducted the experiment on the same gel and included BafA1 treatment. The updated results are presented in **Figure 7E**.

12. In Fig.6J, so many similar colors were show here, which is difficult to review. In addition, why only used higher M cells to study this experiment? It will be better to also use low M to address this issue, thereby compare the difference between high M and low M. In addition, about the evidence to support ICA-induced mitophagy is not enough. More mitophagy-associated markers should be used to confirm this conclusion.

Response: Thank you for your suggestion! We have repeated the relevant experiments. With the addition of new experimental data, we have reorganized the research results. The new high-quality experimental results are presented in **Figure 7G**. Additionally, we have included supplementary experiments confirming that ICA can induce mitophagy, with the related results shown in **Figure 6I**.

13. In Fig.7, the dose and therapeutic protocol should be described in the figure legend. In addition, it will be better to show anti-PD-1 treated alone.

Response: Thank you for your suggestion! We have thoroughly revised and elaborated on the figure legends, and have added a separate anti-PD-1 treatment group. The related results are presented in **Figure 8**.

14. Some English spell is found in the text, like iac in the abstract.

Overall, the finding is novelty. However, many results need to be performed to improve the result's quality and to support the conclusion such as mitophagy and mitochondrial colocalization. Final, it will be better to show the clinical relevance among YBX1, OGT and survival rate.

Response: Thank you for your suggestion! We have supplemented the results related to mitophagy (**Figure 6I, 7G-H**). Additionally, we analyzed the correlation between YBX1 and OGT in clinical tissues, as well as the survival analysis of OGT. The results show that OGT expression is significantly lower in metastatic tissues compared to non-metastatic tissues. High OGT expression indicates a better survival prognosis. Furthermore, in both metastatic and non-metastatic tissues, OGT expression is negatively correlated with YBX1 expression. For details, please refer to **Figure S5C-E**.

Reviewer #4 (Remarks to the Author):

Zhang, Kai et al. YBX1 Confers Immunosuppressive Bone Metastatic Traits in Non-Small Cell Lung Cancer.

This manuscript shows that the transcription factor YBX1 drives both bone metastasis and the

development of an immunosuppressive microenvironment in Non-Small Cell Lung Cancer (NSCLC). YBX1 transcriptionally activates IL6 and CCL5, which promotes metastasis and immune evasion in highly bone-metastatic NSCLC cells. Furthermore, YBX1 is regulated by O-GlcNAcylation at T271, which redirects it to mitochondria for mitophagic degradation and, therefore, limits its nuclear transcriptional activity. In highly bone-metastatic cells, reduced O-GlcNAcylation allows YBX1 accumulation and enhanced IL6/CCL5 signaling. Through small-molecule screening, the authors identify Icaritin (ICA) as a compound that enhances YBX1 O-GlcNAcylation. Authors show that ICA treatment alone reduces bone metastasis, and sensitizes tumors to immunotherapy. Overall, these findings establish YBX1 as a dual-functional therapeutic target for preventing NSCLC bone metastasis and regulating immunotherapy response.
Overall, this paper provides novel mechanistic insight into YBX1 regulation and its role in NSCLC bone metastasis. However, several points require clarification to strengthen the functional connection to regulation by O-GlcNac related to bone metastasis.

Major Points:

1. The role of YBX1 O-GlcNAcylation is interesting. However, it is not well connected to role of YBX1 in bone metastasis. Authors should test whether re-expression of wildtype but not T271A mutant rescues bone metastatic effect shown in sgYBX1 cells in Fig. 1H. Also, not well connected to whether increased O-GlcNAcylation regulates YBX1 degradation (is mutant more stable?) Authors should test whether increasing O-GlcNAcylation (use OGA inhibitor) induces same effect on YBX1 levels and stability as seen with ICA.

Response: Thank you for your suggestion! We designed the following groups for the animal experiments: sgCtrl, sgYBX1, sgYBX1+YBX1^{-WT}, sgYBX1+YBX1$^{T271A\text{-}Mut}$, sgYBX1+YBX1^{-WT}+OGA-i, and sgYBX1+YBX1$^{T271A\text{-}Mut}$+OGA-i. The experimental results indicate that both the wild-type and mutant YBX1 can rescue the effects induced by sgYBX1. However, upon the addition of the OGA inhibitor, the rescue effect of the wild-type is attenuated, while the mutant type remains insensitive to the OGA inhibitor. For detailed results, please refer to **Figure S8**. Additionally, we supplemented the study with OGA inhibitor treatment at the cellular level to simulate the effect of ICA. The results show that OGA inhibitor treatment yields outcomes consistent with those of ICA, namely promoting the degradation of YBX1. These results are detailed in **Figure S9F**.

2. Not clear what cells are being used for Fig. 4C-F (not mentioned in text or Figure Legend). Are these mouse NSCLC cells? Is this a syngeneic model?

Response: Thank you for your comment! **Figure 4C** presents the quantitative analysis of immunofluorescence for the clinical tissue samples shown in **Figure 4B**, while **Figures 4E-G** are based on an orthotopic bone metastasis animal model established using Luci-LLC cells. We have carefully reviewed the manuscript and made the corresponding revisions.

3. Figure 5C IP should be done in one gel to compare High vs Low cells and allow authors to conclude that “OGT-YBX1 interaction is stronger in Low vs High cells”. Same for comparing levels of O-GlcNAc and YBX1 O-GlcNAcylation in Fig. 6F and 6G.

Response: Thank you for your suggestion! We have repeated the relevant experiments as advised, and the results are now presented in **Figure 5C** and **Figure 7E-F.**

4. Figure 5J staining is difficult to see. Since this is a major conclusion of manuscript, authors should provide more convincing data that O-GlcNAcylation at T271 redirects YBX1 to mitochondria (compare cytosol vs mitochondrial fractions).

Response: Thank you for your suggestion! We have performed additional experiments as recommended and analyzed the ratio of YBX1 distribution in the cytoplasmic and mitochondrial fractions. The detailed results are presented in **Figure 5J**, **5L**, and **Figure S7B**.

5. The conclusion that "ICA as an effective inhibitor targeting YBX1-T271 O-GlcNAcylation to remodel YBX1 localization and functional activity" needs further support including functional relevance of T271 (see point 1). More likely ICA either regulates broad pathways including global O-GlcNAcylation thus authors should clearly test whether ICA regulates total O-GlcNAcylation, OGT and OGA levels.

Response: Thank you for your suggestion. We have conducted supplementary experiments, and the results show that ICA has no effect on global O-GlcNAcylation levels or the expression of OGT and OGA (**Figure 7B**). However, it promotes the binding between YBX1 and OGT (**Figure 7C**). This preliminarily indicates that ICA may facilitate the O-GlcNAcylation of YBX1 by enhancing its interaction with OGT. The underlying mechanisms involved will be a key focus of our future research.

6. Fig. 7A its difficult to see any difference between control and ICA treated mice representative luciferase image. Many references not included: Pg. 9, "Prior studies suggest that YBX1 undergoes…" – Pg. 9, "Prior studies indicate that tumor-derived…" P 14, "Given the established link between protein glycosylation and membrane organellelocalization..".

Response: Thank you for your suggestion. We have repeated the relevant experiments, and the results are presented in **Figure 8**. Additionally, regarding the omission of references, we have thoroughly reviewed the manuscript and made the corresponding revisions.

7. Figure 5G – Distinction between red and black is difficult to interpret. Highlight T271 more clearly.

Response: Thank you for your suggestion. We have annotated the amino acids potentially subject to glycosylation modification in the figure. The results displayed in the figure (y7 +810.36) show a mass shift of 810.36 Da, indicating that the change in the molecular weight of the peptide precursor ion is consistent with the theoretical change for O-glycosylation. Furthermore, we have confirmed at the cellular level that T271 does indeed undergo glycosylation modification.

8. Figure 6D-E – Statistical significance is mentioned, but no quantification is provided.

Response: Thank you for your suggestion. As advised, we have performed quantitative analysis. Due to the addition of a substantial amount of new experimental data, we have reorganized the figure order accordingly. The relevant experimental results are now presented in **Figure 7A**.

Minor Points:

1. Abstract – Update Icaritin abbreviation from iac to ICA to match the rest of the paper

Response: Thank you for your suggestion! We have corrected "iac" to "ICA" and thoroughly reviewed the entire manuscript.

2. Graphical Abstract – Bone metastasis representation should be larger.

Response: Thank you for your suggestion! We have redrawn the Graphical Abstract to more clearly reflect our research.

3. Figure 1C – Clarify what bone is being shown (tibia?)

Response: Thank you for your suggestion! The "bone" indicated in Figure 1C (now adjusted to **Figure 1B**) refers to the tibia. We have clearly stated this in both the text and the figure legend.

4. Authors state "Western blot assay proved that the protein level of OGT is higher in A549Low-M cells that that in A549-High-M cells" but don't reference Figure (Fig. 5B). Also, Pg. 8, "Transcriptional reprogramming plays…" – Run-on sentence; split this into two. Add period after "bone metastasis" and begin new sentence with "Thus, we performed…" for clarity.

Response: Thank you for your suggestion! We have thoroughly reviewed the entire manuscript and made the corresponding revisions.

5. Figure 2E – Some numbers in chart on the bottom of the panel are cut off or unclear.

Response: Thank you for your suggestion. We have provided clear and legible figures.

6. Figure 2F – Clarify what channels the first merge represent.

Response: The "first merge" in **Figure 2F** indicates that all patients remained alive during this period, which is attributed to the fact that $EGFR^+$ patients can receive targeted therapy. As a result, these patients tend to have a longer survival period compared to $EGFR^-$ patients.

7. Pg. 12 – Delete "3" in front of subheading "YBX1 transcriptionally activates…" to match subheading format throughout results section.

Response: Thank you for your suggestion. We have thoroughly reviewed the entire manuscript and made the corresponding revisions.

8. Did Icaritin display any off-target effects or toxicities?

Response: Thank you for your suggestion! We have determined the IC50 of ICA in the relevant cells, and the results are shown in **Figure S9A**. The drug concentration used in the experiments was selected based on this IC50 value. Regarding the potential off-target effects of ICA, in the experiment presented in **Figure 7F**, when OGT was knocked down, the effect of ICA on promoting

YBX1 O-GlcNAcylation was attenuated. This result preliminarily suggests that the effect of ICA is specific and reduces the concern regarding significant off-target effects.

Reviewer #5 (Remarks to the Author):

I co-reviewed this manuscript with one of the reviewers who provided the listed reports. This is part of the Nature Communications initiative to facilitate training in peer review and to provide appropriate recognition for Early Career Researchers who co-review manuscripts.

Response: Thank you for your suggestion!

REVIEWER COMMENTS

Reviewer #1 (Remarks to the Author):

Thanks for the revision of the manuscript, the authors have fulfilled all the requests and I have no further comments.

Reviewer #2 (Remarks to the Author):

The authors have included several new experiments to support their conclusions. While some of the responses and added experiments are convincing, others are not. For example, the replacement experiment (new Figure S6A-D) wasn't done properly. Instead of replacing endogenous YBX1 with (equal levels of) the mutant, the authors vastly overexpressed the FLAG-tagged mutant. This set of experiment cannot be interpreted in a meaningful way and should be removed from the paper.

Response: Thank you for your valuable suggestion. We have deleted Figure S6A-D.

In Figure 8, the authors show new experiments addressing the therapeutic potential of ICA. They state that sgYBX1 potentiates the effect of a combined PD-1 inh./ ICA treatment (line 612). This is not the case – in none of the tested settings. Also, the YBX1 mutant, which cannot be O-GlcNAcylated and should be therefore more stable and thus more "metastasizing" as the wt, behaves exactly like YBX1 wt. Why is that? In addition, the authors need to show equal expression levels of endogenous YBX1, YBX1 wt and YBX1 mut. Otherwise the results are hard to interpret.

Response: Thank you for your valuable suggestion. We have provided the protein levels of endogenous YBX1, YBX1-wt, and YBX1-mut. The relevant results are presented in Figure S9A. As for why YBX1-mut did not exhibit stronger metastatic ability than the

wild-type, this may be due to the inherent high invasiveness of LLC cells and the fact that the basal expression of YBX1 has already saturated their metastatic potential. Thus, even the mutant form cannot infinitely amplify this function. Thank you once again for your feedback!

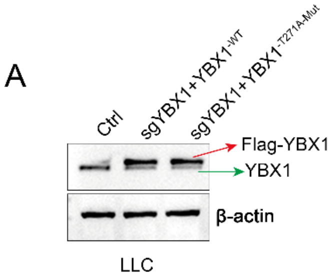

Moreover, the newly presented data in Figure 5 and 6 contain multiple errors (see below), raising general concerns about the overall scientific rigor within the study. So, while I do believe that the main message (role of O-GlcNAcylation for YBX1 stability

and effect of ICA on YBX1 localisation and stabilty) is probably correct and interesting, the revised manuscript is unfortunately not accepable in its current form.

Mistakes in data presentation:

Figure 5I: The figure is obviously mislabelled (YBX1 T271-Mut should be WT) and the WB incorrectly quantified:

Response: Thank you for your valuable suggestion! We sincerely apologize for this error and have corrected it accordingly. Thank you once again for your feedback!

Figure 6E-F: Description in the figure legend does not fit to the shown data. Moreover, in 6E the applied ICA dose is missing in left panel, while incubation time is missing in the right panel.

Figure 6G: figure legend is wrong.

Response: Thank you for your valuable suggestion! We apologize for this oversight and have made the necessary revisions. We appreciate your comments!

Minor:

Define abbreviation OGA

Response: Thank you for your valuable suggestion! We have now defined OGA.

Reviewer #3 (Remarks to the Author):

All the concerns in the previous version have been improved and response in the currently revised drift. I have no suggestion.

Reviewer #4 (Remarks to the Author):

Authors have addressed mall major and minor issues and thus acceptable for publication.

Reviewer #5 (Remarks to the Author):

I co-reviewed this manuscript with one of the reviewers who provided the listed reports. This is part of the Nature Communications initiative to facilitate training in peer review and to provide appropriate recognition for Early Career Researchers who co-review manuscripts.

** See Nature Portfolio’s author and referees' website at www.nature.com/authors for information about policies, services and author benefits.

All the concerns in the previous version have been improved and response in the currently revised drift. I have no suggestion.
